# Genetic Risk Factors Associated With Preeclampsia and Hypertensive Disorders of Pregnancy

**DOI:** 10.1001/jamacardio.2023.1312

**Published:** 2023-06-07

**Authors:** Jaakko S. Tyrmi, Tea Kaartokallio, A. Inkeri Lokki, Tiina Jääskeläinen, Eija Kortelainen, Sanni Ruotsalainen, Juha Karjalainen, Samuli Ripatti, Anna Kivioja, Triin Laisk, Johannes Kettunen, Anneli Pouta, Katja Kivinen, Eero Kajantie, Seppo Heinonen, Juha Kere, Hannele Laivuori

**Affiliations:** 1 Center for Child, Adolescent and Maternal Health Research, Faculty of Medicine and Health Technology, Tampere University, Tampere, Finland; 2 Center for Life Course Health Research, Faculty of Medicine, University of Oulu, Oulu, Finland; 3 Biocenter Oulu, University of Oulu, Oulu, Finland; 4 Department of Medical and Clinical Genetics, University of Helsinki and Helsinki University Hospital, Helsinki, Finland; 5 Department of Bacteriology and Immunology, University of Helsinki and Helsinki University Hospital, Helsinki, Finland; 6 Department of Food and Nutrition, University of Helsinki, Helsinki, Finland; 7 Institute for Molecular Medicine Finland, Helsinki Institute of Life Science, University of Helsinki, Helsinki, Finland; 8 Analytic and Translational Genetics Unit, Massachusetts General Hospital and Harvard Medical School, Boston; 9 Broad Institute of the Massachusetts Institute of Technology and Harvard University, Cambridge; 10 Department of Public Health, University of Helsinki, Helsinki, Finland; 11 Estonian Genome Centre, Institute of Genomics, University of Tartu, Tartu, Estonia; 12 Finnish Institute for Health and Welfare, Helsinki, Finland; 13 PEDEGO Research Unit (Research Unit for Pediatrics, Dermatology, Clinical Genetics, Obstetrics and Gynecology), Medical Research Center Oulu, Oulu University Hospital and University of Oulu, Oulu, Finland; 14 Population Health Unit, Finnish Institute for Health and Welfare, Helsinki and Oulu, Finland; 15 Children’s Hospital, University of Helsinki and Helsinki University Hospital, Helsinki, Finland; 16 Department of Clinical and Molecular Medicine, Norwegian University of Health and Technology, Trondheim, Norway; 17 Department of Obsterics and Gynaecology, University of Helsinki and Helsinki University Hospital, Helsinki, Finland; 18 Department of Biosciences and Nutrition, Karolinska Institutet, Huddinge, Sweden; 19 Folkhälsan Research Center and Stem Cells and Metabolism Research Program, University of Helsinki, Helsinki, Finland; 20 Department of Obstetrics and Gynecology, Tampere University Hospital, Tampere, Finland

## Abstract

**Importance:**

A genetic contribution to preeclampsia susceptibility has been established but is still incompletely understood.

**Objective:**

To disentangle the underlying genetic architecture of preeclampsia and preeclampsia or other maternal hypertension during pregnancy with a genome-wide association study (GWAS) of hypertensive disorders of pregnancy.

**Design, Setting, and Participants:**

This GWAS included meta-analyses in maternal preeclampsia and a combination phenotype encompassing maternal preeclampsia and preeclampsia or other maternal hypertensive disorders. Two overlapping phenotype groups were selected for examination, namely, preeclampsia and preeclampsia or other maternal hypertension during pregnancy. Data from the Finnish Genetics of Pre-eclampsia Consortium (FINNPEC, 1990-2011), Finnish FinnGen project (1964-2019), Estonian Biobank (1997-2019), and the previously published InterPregGen consortium GWAS were combined. Individuals with preeclampsia or other maternal hypertension during pregnancy and control individuals were selected from the cohorts based on relevant *International Classification of Diseases* codes. Data were analyzed from July 2020 to February 2023.

**Exposures:**

The association of a genome-wide set of genetic variants and clinical risk factors was analyzed for the 2 phenotypes.

**Results:**

A total of 16 743 women with prior preeclampsia and 15 200 with preeclampsia or other maternal hypertension during pregnancy were obtained from FINNPEC, FinnGen, Estonian Biobank, and the InterPregGen consortium study (respective mean [SD] ages at diagnosis: 30.3 [5.5], 28.7 [5.6], 29.7 [7.0], and 28 [not available] years). The analysis found 19 genome-wide significant associations, 13 of which were novel. Seven of the novel loci harbor genes previously associated with blood pressure traits (*NPPA*,* NPR3*,* PLCE1*,* TNS2*,* FURIN*,* RGL3*, and *PREX1*). In line with this, the 2 study phenotypes showed genetic correlation with blood pressure traits. In addition, novel risk loci were identified in the proximity of genes involved in the development of placenta (*PGR*,* TRPC6*,* ACTN4*, and *PZP*), remodeling of uterine spiral arteries (*NPPA*,* NPPB*,* NPR3*, and *ACTN4*), kidney function (*PLCE1*,* TNS2*,* ACTN4*, and *TRPC6*), and maintenance of proteostasis in pregnancy serum (*PZP*).

**Conclusions and Relevance:**

The findings indicate that genes related to blood pressure traits are associated with preeclampsia, but many of these genes have additional pleiotropic effects on cardiometabolic, endothelial, and placental function. Furthermore, several of the associated loci have no known connection with cardiovascular disease but instead harbor genes contributing to maintenance of successful pregnancy, with dysfunctions leading to preeclampsialike symptoms.

## Key Points

**Question:**

What are the genetic risk factors associated with preeclampsia and hypertensive disorders of pregnancy?

**Findings:**

In this genome-wide association study, 13 novel preeclampsia- or hypertensive pregnancy–associated genetic loci were discovered. Seven loci are located near genes previously associated with blood pressure traits, and several harbor genes involved in the development of placenta, remodeling of uterine spiral arteries, and kidney function.

**Meaning:**

The findings further strengthen the known association between cardiovascular health and preeclampsia and provide new targets for future research of preeclampsia pathophysiology, including genes involved in placental development and kidney function.

## Teaser

This genome-wide association study seeks to identify genetic risk factors associated with preeclampsia and hypertensive disorders in pregnancy.

## Introduction

Preeclampsia is a vascular pregnancy disorder that affects 3% to 5% of all pregnancies.^1,2^ The disorder develops only in the presence of a placenta, and especially early-onset preeclampsia is often accompanied by defects in placental development and function.^3,4^ Subcellular material and molecules released by the placenta, such as antiangiogenic factors, are thought to evoke the systemic endothelial dysfunction manifested as maternal preeclampsia symptoms, including hypertension and proteinuria.^5,6,7^ However, preeclampsia is both phenotypically and etiologically heterogeneous. The disorder often develops without any evident placental malfunction, and the predisposition to preeclampsia is likely affected by multiple underlying cardiometabolic factors that modify the response to the pregnancy-induced stress.^8^

A genetic contribution to preeclampsia susceptibility has been established,^9,10^ but the actual risk loci remain mostly unknown. The genome-wide association study (GWAS) of preeclampsia by Steinthorsdottir et al^11^ identified 5 risk loci, which have previously been connected to hypertension. Accordingly, epidemiological evidence shows that prior cardiovascular disease inflated the risk of preeclampsia, and individuals with previous preeclampsia were at increased risk of developing cardiovascular disease later in life.^12,13,14,15^

Preeclampsia is likely to consist of several subtypes with differing etiologies.^8^ The overlap in the genetic risk factors between preeclampsia and related disorders could be subtype specific, with some subtypes sharing more features with hypertensive diseases and others being more closely linked to disorders of placental development. Therefore, we have selected 2 phenotype groups for examination: preeclampsia and preeclampsia or other maternal hypertension during pregnancy.

The aim of our study was to identify genetic risk factors associated with preeclampsia and hypertensive disorders of pregnancy in a maternal genome-wide meta-analysis comprising samples from the closely related populations of Finland and Estonia. For the meta-analysis of the strict preeclampsia phenotype, we supplemented these data with a previously published GWAS on preeclampsia.^11^ In addition, we performed GWAS in smaller paternal and fetal sample sets from Finland to identify risk loci of preeclampsia conveyed via the fetus.

## Methods

### Study Phenotypes and Cohorts

For the maternal meta-analyses, genome-wide genotyped and imputed samples from the Finnish Genetics of Pre-eclampsia Consortium (FINNPEC), FinnGen, and Estonian Biobank were used. In addition, we included summary statistics from the earlier meta-analysis study of preeclampsia by Steinthorsdottir et al.^11^ For the fetal and paternal genome-wide association analysis, samples from the FINNPEC cohort were available. All required ethical approvals were obtained from international, national, and regional ethics committees as described in detail in the eAppendix in Supplement 1.

**Figure 1.**
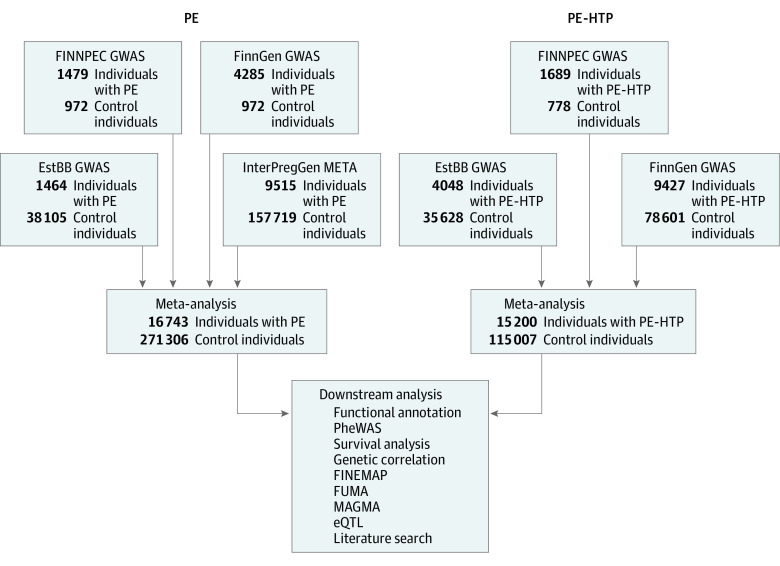
Flow Diagram of the Study Design GWAS indicates genome-wide association study; PE, individuals with preeclampsia; PE-HTP, individuals with preeclampsia or any other type of maternal hypertension during pregnancy.

The analyses were performed with 2 phenotypes: (1) preeclampsia, eclampsia, or preeclampsia superimposed on chronic hypertension and (2) preeclampsia or other maternal hypertensive disorders. We also examined a phenotype of preeclampsia or fetal growth restriction (as mother’s diagnosis), used as proxy for small for gestational age (FinnGen and Estonian Biobank) or diagnoses of placental insufficiency or an infant being small for gestational age (FINNPEC; results were comparatively minor and are presented in the eAppendix in Supplement 1). In the FINNPEC cohort, preeclampsia was defined according to the American College of Obstetricians and Gynecologists 2002 criteria as hypertension (systolic blood pressure ≥140 mm Hg or diastolic blood pressure ≥90 mm Hg) and proteinuria (≥0.3 g/24 hours, 0.3 g/L, or two ≥1+ dipstick readings) occurring after 20 weeks of gestation. Gestational hypertension was defined as hypertension occurring after 20 weeks of gestation in the absence of proteinuria. Birth weights below −2.0 SD units according to the Finnish standards^16^ were classified as small for gestational age. Placental insufficiency was defined as umbilical artery resistance index or pulsatility index of +2 or more SD. In FinnGen and Estonian Biobank, the phenotypes were based on *International Classification of Diseases* codes (*Revisions 8*, *9*, and* 10*, where available) as detailed in eTable 1 in Supplement 1. All parous women not fulfilling the case inclusion criteria were included in the control group. In addition to FINNPEC, FinnGen, and Estonian Biobank, we included the summary statistics from the previous largest genome-wide association meta-analysis for preeclampsia conducted by Steinthorsdottir et al^11^ to the meta-analysis of the preeclampsia phenotype. Further details of the phenotype definitions are presented in the eAppendix in Supplement 1. The sample sizes available in each phenotype are presented in Table 1. The study design is visualized in Figure 1.

**Table 1.** Sample Sizes in the Association Analyses in the FINNPEC, FinnGen, Estonian Biobank, and Steinthorsdottir et al^11^ Studies and in the Maternal Meta-Analyses for the 2 Phenotypes

	Analysis	Individuals with preeclampsia, No.	Control individuals, No.	Total, No.
Cohort				
FINNPEC	PE, maternal	1479	972	2451
Estonian Biobank	PE, maternal	1464	38 105	39 569
FinnGen	PE, maternal	4285	83 285	87 570
Steinthorsdottir et al^11^	PE, maternal	9515	157 719	167 234
Meta-analysis	PE, maternal	16 743	280 081	296 824
FINNPEC	HTP, maternal	1689	778	2467
Estonian Biobank	HTP, maternal	4084	35 628	39 712
FinnGen	HTP, maternal	9427	78 601	88 028
Meta-analysis	HTP, maternal	15 200	115 007	130 207
FINNPEC	PE, fetal	796	894	1690
FINNPEC	HTP, fetal	946	750	1696
FINNPEC	PE, paternal	595	654	1249
FINNPEC	HTP, paternal	697	557	1254

Abbreviations: PE, preeclampsia; PE-HTP, preeclampsia or other maternal hypertensive disorder.

Basic characteristics of all cohorts are described in eTable 2 in Supplement 1. Detailed description is provided in eTable 3 in Supplement 1 for FINNPEC, as this case-control cohort contains more extensive clinical information compared to the other cohorts. We also report survival analysis between preeclampsia and all other disease end points available in FinnGen. The specifics of this workflow are described in detail in the eAppendix in Supplement 1. The analyses of placental insufficiency or infants who were small for gestational age phenotype yielded limited findings, and the results are discussed in the eAppendix, eTables 11 and 12, and eFigures 6-9 in Supplement 1.

### Genotyping and Imputation

Genotyping was performed using Illumina and Affymetrix arrays (Illumina and Thermo Fisher Scientific) in FINNPEC, FinnGen, and Estonian Biobank. Only high-imputation quality markers (imputation information score >0.7) were used in the analysis. Exact genotyping workflows are detailed in eAppendix in Supplement 1.

### Association and Meta-Analyses

In brief, the association analyses were performed for all genotyped variants using generalized mixed model as implemented in SAIGE versions 0.39, 0.39.1, and 0.38 (Lee Lab for Statistical Genetics and Data Science^17^) for FINNPEC, FinnGen, and Estonian Biobank, respectively. Summary statistics obtained from these analyses in each cohort were then used to perform inverse variance–weighted meta-analysis with METAL software.^18^ Detailed associations and the meta-analysis workflow are presented in the eAppendix in Supplement 1.

### Annotation of Loci

To identify plausible candidate genes in each locus, we prioritized genes according to multiple layers of evidence: (1) statistical significance of variants in the GWAS meta-analysis, (2) functional evidence associated with the discovered variants, and (3) careful examination of literature regarding the identified variants and suspected causal genes. The detailed annotation workflow is described in the eAppendix in Supplement 1.

### Genetic Correlations

To further examine the correlation of our findings with other disorders, we first conducted a phenome-wide association study (PheWAS) analysis for the lead variants using all 2861 phenotypes provided in FinnGen Data Freeze 6. Second, we used Linkage Disequilibrium Score Regression software^19,20^ to evaluate genetic correlation of the studied phenotypes with other traits. Third, we calculated polygenic risk scores (PRS) for preeclampsia and preeclampsia or other maternal hypertensive disorder phenotypes and analyzed the results in the FINNPEC cohort to examine overall genetic contribution uncovered in our GWAS analysis. The methodology of these 3 analyses is explained in detail in the eAppendix in Supplement 1. Data were analyzed from July 2020 to February 2023. The genome-wide association data generated in this study have been deposited in the NHGRI-EBI GWAS Catalogue database.

## Results

A total of 16 743 women with prior preeclampsia and 15 200 with preeclampsia or other maternal hypertension during pregnancy were obtained from FINNPEC, FinnGen, Estonian Biobank, and the InterPregGen consortium study. The mean (SD) age in each cohort was 30.3 [5.5], 28.7 [5.6], 29.7 [7.0], and 28 [not available] years, respectively.

### Association Signals From the Maternal Meta-Analyses

**Figure 2.**
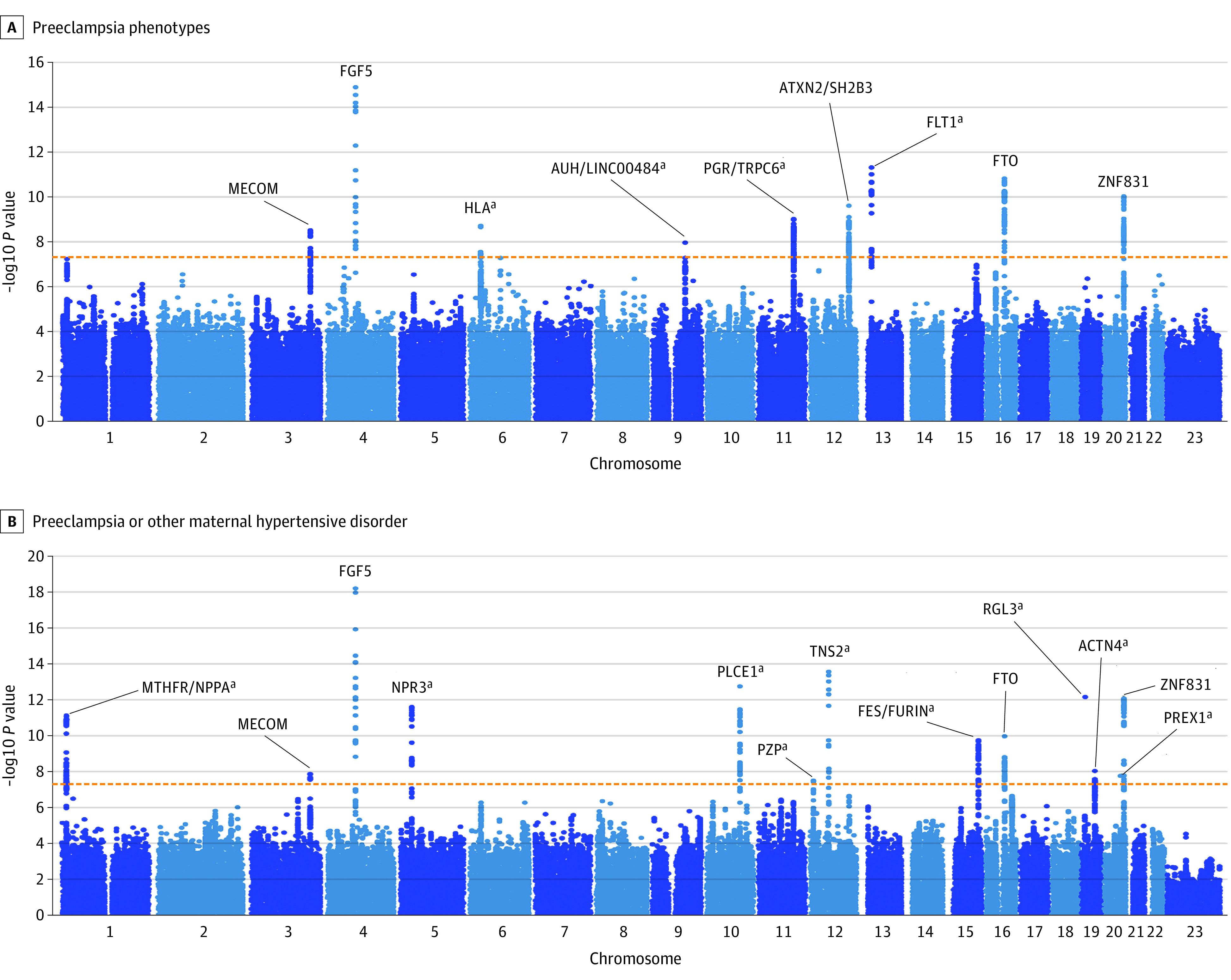
Manhattan Plot for the Meta-Analysis Results of Preeclampsia and Preeclampsia or Other Maternal Hypertensive Disorder Phenotypes With Genome-Wide Significant Loci Labeled With Most Likely Candidate Gene ^a^Novel loci not detected in previous genome-wide association studies.

Altogether, we identified 9 and 13 genome-wide significant loci for the preeclampsia and the preeclampsia or other maternal hypertensive disorder phenotypes, respectively (Table 2, Figures 2, and eFigures 1-5 in Supplement 1). Four preeclampsia loci in the meta-analysis and 9 preeclampsia or other maternal hypertension loci in the meta-analysis were not significantly associated with preeclampsia in the earlier maternal GWAS.

**Table 2.** Lead Variants of the Genome-Wide Significant Loci From the Maternal Meta-Analyses in the Preeclampsia (PE) and Preeclampsia or Other Maternal Hypertensive Disorder (PE-HTP) Phenotypes

Phenotype	rsID	Cytoband	Chromosome: position	Proposed candidate gene	Effect allele/other allele	Effect allele frequency	Odds ratio (95% CI)	*P* value	+/−^b^
PE	rs4245909	3q26	3:169172788	MECOM	G/A	0.51	0.92 (0.90-0.95)	3.19 × 10^−09^	−−−−
PE	rs16998073	4q21	4:81184341	FGF5	T/A	0.32	1.12 (1.09-1.15)	1.33 × 10^−15^	++++
PE	rs2596471	6p21	6:31428911	HLA/PSORS1C2^a^	G/A	0.81	1.11 (1.07-1.15)	1.98 × 10^−09^	++++
PE	rs7862828	9q22	9:93919803	AUH/LINC00484^a^	C/A	0.75	1.10 (1.06-1.13)	1.12 × 10^−08^	++++
PE	rs3018700	11q22	11:101266410	PGR/TRPC6^a^	C/T	0.85	1.13 (1.09-1.18)	9.98 × 10^−10^	++++
PE	rs10774624	12q24	12:111833788	ATXN2/SH2B3	A/G	0.59	0.92 (0.89-0.94)	2.52 × 10^−10^	−−+−
PE	rs7318880	13q12	13:29138285	FLT1^a^	T/C	0.48	1.10 (1.07-1.13)	5.04 × 10^−12^	++++
PE	rs1421085	16q12	16:53800954	FTO	C/T	0.41	1.10 (1.07-1.13)	1.55 × 10^−11^	++++
PE	rs6026744	20q13	20:57742388	ZNF831	T/A	0.15	1.13 (1.09-1.17)	9.72 × 10^−11^	++−+
PE-HTP	rs13306561	1p36	1:11865804	MTHFR/NPPA^a^	G/A	0.14	0.88 (0.84-0.91)	7.49 × 10^−12^	−−−
PE-HTP	rs1918969	3q26	3:169139890	MECOM	C/T	0.47	0.93 (0.90-0.95)	1.34 × 10^−08^	−−+
PE-HTP	rs16998073	4q21	4:81184341	FGF5	T/A	0.33	1.14 (1.11-1.17)	6.27 × 10^−19^	+++
PE-HTP	rs12656497	5p13	5:32831939	NPR3^a^	C/T	0.58	1.10 (1.07-1.13)	2.52 × 10^−12^	+++
PE-HTP	rs10882398	10q23	10:95892788	PLCE1^a^	A/T	0.59	1.11 (1.08-1.14)	1.77 × 10^−13^	+++
PE-HTP	rs10843404	12p13	12:9471215	PZP^a^	C/G	0.47	1.08 (1.05-1.11)	3.16 × 10^−08^	+++
PE-HTP	rs117928258	12q13	12:53457585	TNS2^a^	T/C	0.02	1.45 (1.32--1.60)	2.74 × 10^−14^	+++
PE-HTP	rs6224	15q26	15:91423543	FURIN/FES^a^	T/G	0.42	1.09 (1.06-1.12)	1.77 × 10^−10^	+++
PE-HTP	rs113935429	16q12	16:53822169	FTO	A/AT	0.38	1.10 (1.07-1.13)	1.07 × 10^−10^	+++
PE-HTP	rs167479	19p13	19:11526765	RGL3^a^	T/G	0.44	0.90 (0.88-0.93)	6.95 × 10^−13^	−−+
PE-HTP	rs979971	19q13	19:39144244	ACTN4^a^	T/C	0.47	0.92 (0.90-0.95)	9.10 × 10^−09^	−−−
PE-HTP	rs2208589	20q13.13	20:47408414	PREX1^a^	G/A	0.87	1.12 (1.08-1.17)	1.70 × 10^−08^	+++
PE-HTP	rs201454025	20q13.32	20:57757760	ZNF831	G/GTGTT	0.17	1.14 (1.10-1.18)	8.41 × 10^−13^	+++

^a^ Novel loci.

^b^ Direction of effect in FinnGen, Estonian Biobank, FINNPEC, and Steinthorsdottir et al^11^ in the PE phenotype as the fourth direction.

Preliminary meta-analysis results showed evidence of slight inflation in the meta-analysis test statistics (linkage disequilibrium score intercepts of 1.0291 [SE, 0.0076] and 1.0325 [SE, 0.0079] for preeclampsia and preeclampsia or other maternal hypertension during pregnancy, respectively) and were corrected by rerunning the meta-analysis with genomic correction. Most of the directions of the associations were concordant between the cohorts with the exception of 4 loci on 3q26, 12q24, 19p13, and 20q13 that showed discordant association of direction in the smallest cohort (FINNPEC) compared to the others (Table 2). We did not observe genetic heterogeneity except for the lead variant rs167479 at locus 19p13. Credible sets produced with SuSie version 0.11.92 are listed in eTable 4 in Supplement 2.

In the survival analysis conducted in FinnGen between preeclampsia and all other end points, preeclampsia was associated with an increase in risk of 146 disease end points (eTable 5 in Supplement 1). These included hypertensive diseases, such as gestational hypertension and cardiovascular disease, but also glomerular diseases and glomerulonephritis. Furthermore, increased risk was shown for placental abruption and numerous complications related to induced labor, such as failed induction of labor and infections of genitourinary tract in pregnancy.

In the genetic correlation analysis between our study phenotypes and 894 previously published phenotypes, the preeclampsia phenotypes were correlated most strongly with the phenotypes related to blood pressure or various cardiovascular disease (eTable 6 in Supplement 2). For both phenotypes, the correlations with blood pressure medication and high blood pressure were above 0.59 and 0.56, respectively, and correlations with coronary artery disease were above 0.4. In addition, several measures of body fat were associated with our phenotypes. These findings are also mirrored by the results of the PheWAS analysis conducted for all the lead variants in FinnGen Data Freeze 6 (eTable 7 in Supplement 2). In this test, 11 of 22 lead variants were significantly associated with essential hypertension or cardiovascular disease and 7 with other phenotypes, whereas for 4 variants, no significant associations were found. PheWAS also detected associations to immunology- and autoimmunity-related end points for many lead variants. For instance, various arthritis and rheumatic end points were associated with 4 loci (6p21, 12q13, 12q24, and 16q12).

### Several Novel Risk Loci for Preeclampsia and Hypertensive Disorders of Pregnancy Revealed in Meta-Analyses 

**Figure 3.**
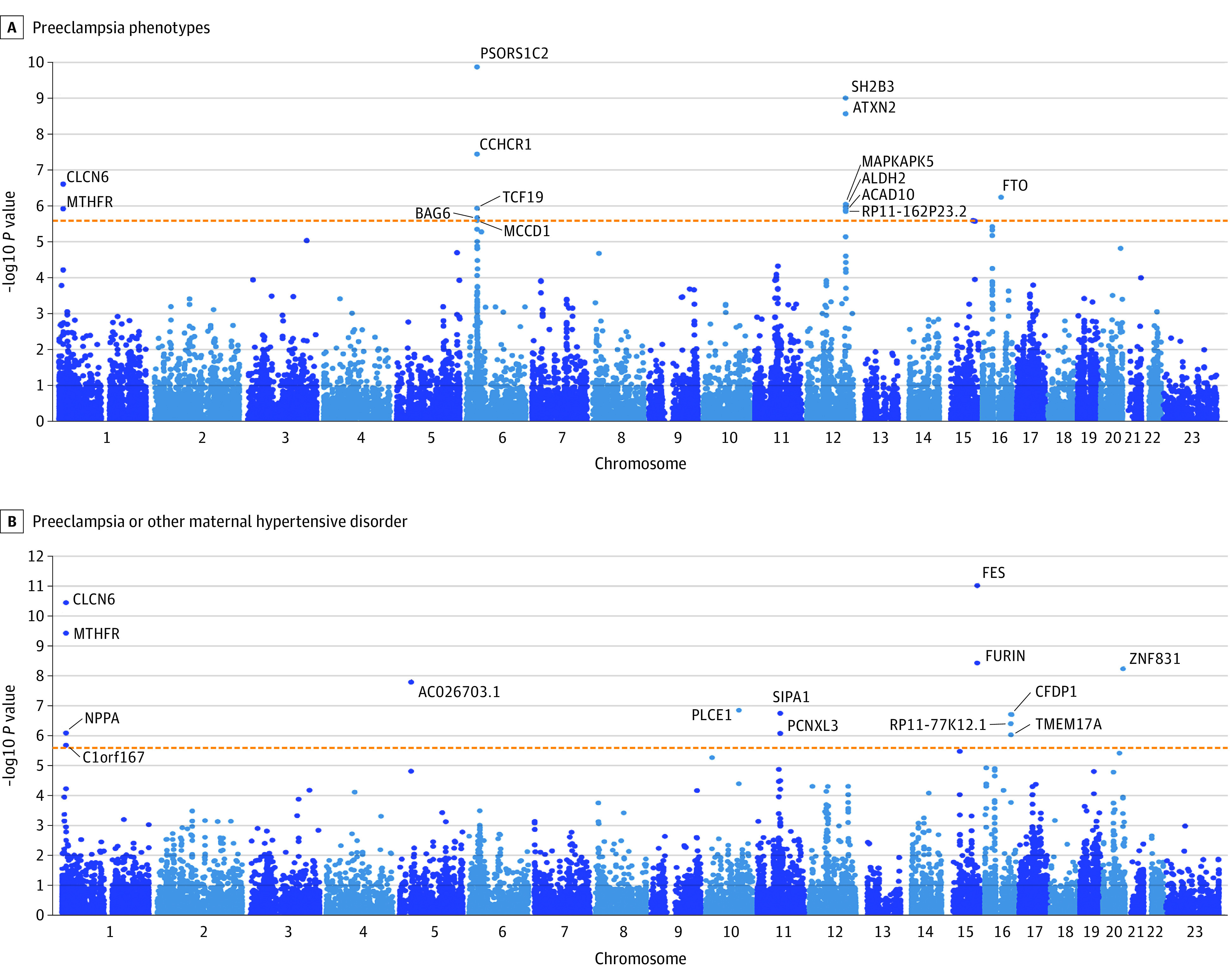
MAGMA Gene-Based Test Results for the Meta-Analysis Results of the Preeclampsia and Preeclampsia or Other Maternal Hypertensive Disorder Phenotypes

In the preeclampsia meta-analysis, we replicated all 5 loci reported by Steinthorsdottir et al^11^ and identified 4 novel genome-wide significant loci of maternal preeclampsia (Figure 2A). Plausible candidate genes were identified for all the novel loci, except for the association at 9q22, where the candidate gene remained elusive for the preeclampsia phenotype. Novel association peaks were detected at locus 13q12 close to *FLT1* and at 11q22, where the intergenic variant rs3018700 lies near genes *PGR* and *TRPC6.* We also found a genome-wide significant association within the human leukocyte antigen (HLA) region, which is known for its high density of genes involved in immune regulation and recognition. The association lies within an 89 kbp copy number variations region previously associated with preeclampsia^21^ and is located within the major susceptibility locus for psoriasis, *PSORS1*. In addition, MAGMA gene-based analysis yielded significant results for ‘*MTHFR* and *CLCN6* genes (Figure 3A).

The preeclampsia or other maternal hypertension during pregnancy meta-analysis revealed 9 novel loci not identified by Steinthorsdottir et al^11^ or in the preeclampsia phenotype studied here (Figure 2B). Four of the 5 associations identified in the GWAS of preeclampsia by Steinthorsdottir et al^11^ were replicated, with only the locus in 12q24 remaining slightly below genome-wide significance. Association in chromosome 1 locus 1p36 was identified by both the meta-analysis and the MAGMA analysis (Figure 3B), which implicated the *MTHFR*, *CLCN6*, and *NPPA* genes. Other discovered loci harbor genes *NPR3*, *PLCE1*, *PZP*, *TNS2*, *FURIN*, *FES*, *RGL3, ACTN4*, and *PREX1*. The variants near *PZP* on 12p13 and *ACTN4* on 19q13 did not appear to contain any genome-wide significant variants directly contributing to hypertensive diseases or genes associated with hypertensive disorders in close proximity, unlike the other discovered loci for preeclampsia or other maternal hypertension during pregnancy. Two additional loci residing in 11q13.1 and 16q23.1 were detected in MAGMA analysis (Figure 3B).

### Association of High PRS for Preeclampsia and Preeclampsia or Other Maternal Hypertension During Pregnancy With Risk of Preeclampsia

Our analyses in the FINNPEC cohort comparing individuals in the top 10% preeclampsia PRS compared to those in the bottom 90% showed an odds ratio (OR) of 2.21 (95% CI, 1.58-3.10; *P* < .001) for preeclampsia and 1.89 (95% CI, 1.42-2.52; *P* < .001) for preeclampsia with severe symptoms. When using the top and bottom percentiles of PRS for preeclampsia or other maternal hypertension during pregnancy, similar associations were shown for the preeclampsia or other maternal hypertension phenotype (OR, 3.66; 95% CI, 2.36-5.69; *P* < .001). The Nagelkerke *R*^2^ values calculated for models, including risk factors of age, parity, body mass index, and first trimester blood pressure, showed improvement when PRS information was added, as described in the eAppendix and eTables 8-10 in Supplement 1.

### Paternal and Fetal Association Analyses in the FINNPEC Cohort

The GWASes of paternal and fetal preeclampsia and preeclampsia or other maternal hypertension during pregnancy did not yield any genome-wide significant associations. Further details can be found in eFigure 5 in Supplement 1.

## Discussion

In this GWAS, we identified multiple novel risk loci for the preeclampsia only and preeclampsia or other maternal hypertensive disorder phenotypes. The discovered loci harbor genes affecting endothelial dysfunction, placental development, and immunology. Six of the 9 loci in the preeclampsia phenotype and 11 of 13 in the preeclampsia or other maternal hypertension loci reached genome-wide significant association in previous GWASes of cardiovascular diseases. This observation, along with our genetic correlation and PheWAS results, imply that the established blood pressure loci are associated with predisposition to hypertension during pregnancy, plausibly via the same mechanisms. The findings of our study, and the results of the survival analysis in particular, support the concept of pregnancy as a window to future cardiovascular health: the increased maternal genetic susceptibility to cardiovascular disease might become evident for the first time during pregnancy.

Systemic endothelial dysfunction characterized by impaired vasodilation, endothelial injury, and reduction in vascular integrity is central to the pathophysiology of preeclampsia.^22,23,24^ The best known biomarker of preeclampsia, sFlt-1, is a soluble antiangiogenic protein that reduces the availability of the proangiogenic proteins *VEGF* and *PlGF* to endothelial cells, thus impairing the maintenance of vascular integrity and cellular viability.^25,26^ The earlier largest GWAS^11^ found an association between maternal preeclampsia and the locus in the fetal genome.^27^ Our study provides evidence of the relevance of the *FLT1* gene in the genomes of women with preeclampsia. In addition, *NPPA* on 1p36, *FES* and *FURIN* on 15q26, *ACTN4* on 19q13, and *PREX1* on 20q13.13 encode for proteins that are involved in regulating endothelial permeability and leukocyte transmigration.^28,29,30,31,32^ These findings provide further support to the idea that preeclampsia liability might be modified by alterations in the integrity of the endothelium.

Abnormal leakage of protein to urine is another key feature of preeclampsia. Intriguingly, several of the genes proximal to the associating lead single-nucleotide variants in our study have been associated with kidney disease. Mutations in *PLCE1*, *TNS2*,* ACTN4*, and *TRPC6* are associated with nephrotic syndrome characterized by proteinuria.^33,34,35,36,37,38^ Products of these genes have important roles in podocyte function and integrity of the glomerular filtration barrier.^39,40,41,42^ The mechanisms of action of these genes in causing kidney damage are likely to be variable. It is plausible that such genetic predisposition and putative kidney injuries sustained during preeclampsia may contribute to later glomerular diseases, as suggested by our survival analyses and other literature.^43^

Examples of putative pleiotropic candidate genes of the associating loci in our study include the natriuretic peptide genes *NPPA* and *NPPB* on 1p36 and their clearance receptor *NPR3* on 5p13. Natriuretic peptide hormones regulate blood pressure and kidney function, among their several other effects.^44^ In addition, mice that lack the expression of atrial natriuretic peptide develop gestational hypertension and proteinuria and, similar to preeclampsia, exhibit impairment in trophoblast invasion and uterine spiral artery remodeling.^45,46^ Changes in the function or expression level of these genes with several effects in the key mechanisms of preeclampsia could contribute to the multiorgan dysfunction that is characteristic of this pregnancy-related disorder.

Many of the genes next to the lead single-nucleotide variants of our study are involved in placental development and function, which are often compromised in preeclampsia. Both genes closest to the lead variant in the locus 11q22, *PGR* and *TRPC6*, are known to affect placental functions and maintenance of pregnancy.^47,48^ *PGR* has been suggested to contribute to balanced hormonal signaling during pregnancy and subsequently aid the immune and endothelial cells in the cytotrophoblast invasion.^48^ Also, the 19q13-located *ACTN4* is known for regulating the trophoblast proliferation and differentiation during early pregnancy.^49^ Defects in these processes are well documented, especially in early-onset preeclampsia.^3,4^ Another pregnancy-related gene, *PZP* (9q13), is a protease inhibitor that prevents the activity of all 4 classes of proteases and stabilizes misfolded proteins, which have been shown to accumulate in preeclampsia.^50,51,52,53,54^ Additional relevant literature regarding these putative candidate genes is reviewed in more detail in the eAppendix in Supplement 1.

Immunological factors have been shown to contribute to the pathophysiology of preeclampsia.^55^ Examples of associating genes putatively modulating the immunological response to pregnancy include *PZP* (in the preeclampsia or other maternal hypertension phenotype), likely modulating T helper cell response and *PSORS1C2* (within the PSORS1 locus in the HLA region). Tissue compatibility, autoimmunity, regulation of inflammation, and cardiovascular diseases are processes with known susceptibility loci within the HLA and pathophysiological relevance in preeclampsia. Our discovered association largely reflects these functions, as discussed in more detail in the eAppendix in Supplement 1. In line with these observations, PheWAS analysis found several associations with immunology-related disorders (although not psoriasis), such as type 1 diabetes, spondylopathies, rheumatoid arthritis, and, to a lesser degree, hypertension. Immunological etiology is typically associated with severe or early-onset preeclampsia.^56^ Due to the high gene density, extreme polymorphism, and complex haplotype structure, identifying likely candidate genes, especially in the HLA region, is challenging. Until now, immunology-related findings have been absent in preeclampsia GWAS results.

When comparing the preeclampsia and preeclampsia or other maternal hypertension phenotypes explored in this study, we notice that the effect sizes of the uncovered loci were largely similar between them. The most likely candidate genes appear to be related to hypertensive diseases, implying that the genetic risk factors of preeclampsia are shared with other hypertensive disorders as well as risk of hypertension later in life. Furthermore, incorporation of PRS to already known risk factors of preeclampsia may yield improvement in prediction of this disorder.

The main strength of this study is the use of the 3 well-characterized cohorts FINNPEC, FinnGen, and Estonian Biobank, originating from 2 closely related populations of Finland and Estonia. Such homogenous populations might facilitate the discovery of rare variants with larger effects and characterization of the genetic basis of complex diseases such as preeclampsia.^57,58^ The associations previously reported by Steinthorsdottir et al^11^ are similar in effect directions to those now discovered in the preeclampsia phenotype in the Finnish and Estonian cohorts, suggesting that similar genetic background may contribute to preeclampsia in other populations as well.

### Limitations

This study has limitations. Generalizability of the results may be limited in more diverse populations. Lack of external validation is also a limitation, although the consistency in effect sizes between the large study cohorts does add to the reliability of the findings. The register-based approach used in this study provides limited phenotype information and can be seen as a limiting factor, although the quality of the Finnish Care Register for Health Care has previously been shown to be excellent.^59^ In support of the robustness of our approach, we replicated the findings of the largest previously published GWAS meta-analysis, both with our preeclampsia and preeclampsia or other maternal hypertension phenotypes and all the study cohorts involved provided uniform support for our findings with little evidence of heterogeneity. Uncovering the paternal and fetal associations may require considerably larger sample sizes than available in the current study. Our PRS analysis has 2 important limitations. First, as the FINNPEC cohort was collected from university hospitals, the included individuals may represent those with a more severe form of the disease. Second, due to its being a case-control cohort, FINNPEC has larger proportion of individuals with preeclampsia compared to other cohorts. Together these factors may lead to inflated estimates of the predictive ability of PRS reported in this work.

## Conclusions

Our study uncovered 13 novel loci with genome-wide significant association with preeclampsia or other hypertensive disorders of pregnancy. We found that cardiovascular disease–related genes were associated with preeclampsia, as previously suggested, but many of those genes have pleiotropic effects on cardiometabolic, endothelial, and placental function. In addition, we provide further evidence for an association of several loci not previously associated with cardiovascular disease but containing genes with apparent importance in the maintenance of pregnancy, with dysfunctions leading to preeclampsialike symptoms. Although further functional studies are required in the future, these results offer valuable insights into the genetic architecture and biology behind preeclampsia as well as into the connection between preeclampsia and other maternal hypertensive disorders.

## Notes

Supplement 1. eAppendix eTable 1. International Classification of Diseases and Related Health Problems (ICD) codes for the FinnGen and Estonian biobank eTable 2. Age and BMI of the cases of the three phenotypes in each study cohort eTable 3. Additional clinical variables available in FINNPEC eTable 5. Survival analyses between preeclampsia and all other FinnGen disease endpoints eTable 8. Odds ratios corresponding to the top 10 % versus bottom 90 % PRSs eTable 9. Case count in top PRS deciles eTable 10. Nagelkerkes’s R2 for models including clinical risk factors and the calculated PRSs eTable 11. Sample sizes in the association analyses in the FINNPEC, FinnGen and Estonian Biobank preeclampsia or fetal growth restriction phenotype eTable 12. Lead variants of the genome-wide significant loci from the maternal meta-analysis in the preeclampsia or fetal growth restriction phenotype. Novel loci are bolded eFigure 1. Forest plot of meta-analysis results for each different phenotype eFigure 2. Regional association plots for genome wide significant associations of preeclampsia meta-analysis eFigure 3. Regional association plots for genome wide significant associations of the preeclampsia or other maternal hypertensive disorder phenotype for chromosomes 1-15 eFigure 4. Regional association plots for genome wide significant associations of the preeclampsia or other maternal hypertensive disorder phenotype for chromosomes 16-20 eFigure 5. Manhattan plot for preeclampsia lead variants in paternal and child samples eFigure 6. Flow chart of the study design for the preeclampsia or fetal growth restriction phenotype eFigure 7. Manhattan plot for the meta-analysis results of the preeclampsia or fetal growth restriction phenotype eFigure 8. MAGMA gene-based test results for the meta-analysis results of the preeclampsia or fetal growth restriction phenotype eFigure 9. Regional association plot preeclampsia or fetal growth restriction lead variants

Supplement 2. eTable 4. Variants included in the credible sets of SuSie finemapping eTable 6. Pairwise genetic correlation results eTable 7. PheWAS results for preeclampsia in FinnGen

Supplement 3. Nonauthor collaborators

Supplement 4. Data sharing statement
