# Supplementary material for: Genetic Risk Factors Associated With Preeclampsia and Hypertensive Disorders of Pregnancy
Source: JAMA Cardiol. 2023 Jun 7;8(7):674–83. doi: 10.1001/jamacardio.2023.1312 (PMC10248811; doi:10.1001/jamacardio.2023.1312)
Supplement: Supplement 1. — eAppendix eTable 1. International Classification of Diseases and Related Health Problems (ICD) codes for the FinnGen and Estonian biobank eTable 2. Age and BMI of the cases of the three phenotypes in each study cohort eTable 3. Additional clinical variables available in FINNPEC eTable 5. Survival analyses between preeclampsia and all other FinnGen disease endpoints eTable 8. Odds ratios corresponding to the top 10 % versus bottom 90 % PRSs eTable 9. Case count in top PRS deciles eTable 10. Nagelkerkes’s R2 for models including clinical risk factors and the calculated PRSs eTable 11. Sample sizes in the association analyses in the FINNPEC, FinnGen and Estonian Biobank preeclampsia or fetal growth restriction phenotype eTable 12. Lead variants of the genome-wide significant loci from the maternal meta-analysis in the preeclampsia or fetal growth restriction phenotype. Novel loci are bolded eFigure 1. Forest plot of meta-analysis results for each different phenotype eFigure 2. Regional association plots for genome wide significant associations of preeclampsia meta-analysis eFigure 3. Regional association plots for genome wide significant associations of the preeclampsia or other maternal hypertensive disorder phenotype for chromosomes 1-15 eFigure 4. Regional association plots for genome wide significant associations of the preeclampsia or other maternal hypertensive disorder phenotype for chromosomes 16-20 eFigure 5. Manhattan plot for preeclampsia lead variants in paternal and child samples eFigure 6. Flow chart of the study design for the preeclampsia or fetal growth restriction phenotype eFigure 7. Manhattan plot for the meta-analysis results of the preeclampsia or fetal growth restriction phenotype eFigure 8. MAGMA gene-based test results for the meta-analysis results of the preeclampsia or fetal growth restriction phenotype eFigure 9. Regional association plot preeclampsia or fetal growth restriction lead variants [file jamacardiol-e231312-s001.pdf]

Supplemental Online Content

Tyrmi JS, Kaartokallio T, Lokki AI, et al. Genetic risk factors associated with preeclampsia and hypertensive disorders of pregnancy. *JAMA Cardiol*. Published online June 7, 2023. doi:10.1001/jamacardio.2023.1312

eAppendix

Note 1: Ethical considerations

Note 2: Study population and cohort descriptions

Note 3: Survival analyses between preeclampsia and other FinnGen endpoints

Note 4: Genotyping and imputation

Note 5: Association and meta-analysis

Note 6: Annotation of loci

Note 7: Genetic correlations

Note 8: Polygenic risk scores (PRS)

Note 9: Literature lookup of genes at novel identified risk loci

Note 10: Study of preeclampsia or indication of fetal growth restriction

eTable 1. International Classification of Diseases and Related Health Problems (ICD) codes for the FinnGen and Estonian biobank

eTable 2. Age and BMI of the cases of the three phenotypes in each study cohort

eTable 3. Additional clinical variables available in FINNPEC

eTable 5. Survival analyses between preeclampsia and all other FinnGen disease endpoints

eTable 8. Odds ratios corresponding to the top 10 % versus bottom 90 % PRSs

eTable 9. Case count in top PRS deciles

eTable 10. Nagelkerkes's R2 for models including clinical risk factors and the calculated PRSs

eTable 11. Sample sizes in the association analyses in the FINNPEC, FinnGen and Estonian Biobank preeclampsia or fetal growth restriction phenotype

eTable 12. Lead variants of the genome-wide significant loci from the maternal meta-analysis in the preeclampsia or fetal growth restriction phenotype. Novel loci are bolded

eFigure 1. Forest plot of meta-analysis results for each different phenotype

eFigure 2. Regional association plots for genome wide significant associations of preeclampsia meta-analysis

eFigure 3. Regional association plots for genome wide significant associations of the preeclampsia or other maternal hypertensive disorder phenotype for chromosomes 1-15

eFigure 4. Regional association plots for genome wide significant associations of the preeclampsia or other maternal hypertensive disorder phenotype for chromosomes 16-20

eFigure 5. Manhattan plot for preeclampsia lead variants in paternal and child samples

eFigure 6. Flow chart of the study design for the preeclampsia or fetal growth restriction phenotype

eFigure 7. Manhattan plot for the meta-analysis results of the preeclampsia or fetal growth restriction phenotype

eFigure 8. MAGMA gene-based test results for the meta-analysis results of the preeclampsia or fetal growth restriction phenotype

eFigure 9. Regional association plot preeclampsia or fetal growth restriction lead variants

This supplemental material has been provided by the authors to give readers additional information about their work.

© 2023 Tyrmi JS et al. *JAMA Cardiology.*

eAppendix

Note 1: Ethical considerations

The FINNPEC study was approved by the Coordinating Ethics Committee of the Hospital District of Helsinki and Uusimaa (149/EO/2007). All FINNPEC study participants and the participating parents of the neonates provided a written informed consent.

Patients and control subjects in the FinnGen study provided informed consent for biobank research, based on the Finnish Biobank Act. Alternatively, older research cohorts, collected prior the start of FinnGen (in August 2017), were collected based on study-specific consents and later transferred to the Finnish biobanks after approval by Fimea, the National Supervisory Authority for Welfare and Health. Recruitment protocols followed the biobank protocols approved by Fimea. The Coordinating Ethics Committee of the Hospital District of Helsinki and Uusimaa (HUS) approved the FinnGen study protocol Nr HUS/990/2017.

The FinnGen study is approved by the Finnish Institute for Health and Welfare (permit numbers: THL/2031/6.02.00/2017, THL/1101/5.05.00/2017, THL/341/6.02.00/2018, THL/2222/6.02.00/2018, THL/283/6.02.00/2019, THL/1721/5.05.00/2019, THL/1524/5.05.00/2020, and THL/2364/14.02/2020), the Digital and population data service agency (permit numbers: VRK43431/2017-3, VRK/6909/2018-3, VRK/4415/2019-3), the Social Insurance Institution (permit numbers: KELA 58/522/2017, KELA 131/522/2018, KELA 70/522/2019, KELA 98/522/2019, KELA 138/522/2019, KELA 2/522/2020, KELA 16/522/2020 and the Statistics Finland (permit numbers: TK-53-1041-17 and TK-53-90-20).

The Biobank Access Decisions for the FinnGen samples and the data utilized in the FinnGen Data Freeze 6 include: THL Biobank BB2017_55, BB2017_111, BB2018_19, BB_2018_34, BB_2018_67, BB2018_71, BB2019_7, BB2019_8, BB2019_26, BB2020_1, Finnish Red Cross Blood Service Biobank 7.12.2017, Helsinki Biobank HUS/359/2017, Auria Biobank AB17-5154, Biobank Borealis of Northern Finland_2017_1013, Biobank of Eastern Finland 1186/2018, Finnish Clinical Biobank Tampere MH0004, Central Finland Biobank 1-2017, and Terveystalo Biobank STB 2018001.

The Estonian Biobank (EstBB) is a population-based biobank that has obtained clinical data from the national registries and hospital databases. Analyses in the EstBB were carried out under ethical approval 1.1-12/624 from

© 2023 Tyrmi JS et al. *JAMA Cardiology.*

the Estonian Committee on Bioethics, and Human Research and data release N05 from the EstBB. All biobank participants have signed a broad informed consent form.

Note 2: Study population and cohort descriptions

The FINNPEC study is a nationwide preeclampsia case-control cohort recruited from five university hospitals in Finland. The cohort consist of both prospectively and retrospectively recruited women, as well as spouses and neonates of the participating women in the prospective arm of the cohort. All participants are of Finnish ancestry. Blood samples for DNA extraction were collected from all the participants, and first and third trimester serum samples as well as placental samples from a subset of the women. In addition, extensive clinical information collected from hospital records of the participants' obstetric histories, pregnancy complications and outcomes, and laboratory, blood pressure and proteinuria measurements during pregnancy, as well as information on delivery and the newborn have been obtained. Women with multiple or ovum donation pregnancy, age below 18 years, or an inability to provide informed consent based on information in Finnish or Swedish were excluded. Diagnoses were established based on medical records and confirmed independently by a research nurse and a study physician. A total of 1479, 1689 and 1564 FINNPEC cases of preeclampsia, preeclampsia or other maternal hypertensive disorder and preeclampsia or fetal growth restriction, respectively, were available for the maternal meta-analyses. A full list of the FINNPEC investigators can be found in Supplement 5.

In FinnGen the study phenotypes are based on ICD-10, ICD-9 and ICD-8 codes as described in eTable 1. The FinnGen project combines genotype data from Finnish nationwide biobanks that are linked with digital health records from the national hospital discharge (from 1968 onwards), cancer (1953-), death (1969-) and medication reimbursement (1995-) registers. All participants are of Finnish ancestry. The Data Freeze 6 used in this study contains the genomic and health record data for 6% of adult Finnish women. In FinnGen, 88028 women with offspring were included in the study. FinnGen contributed a total of 4285, 9427 and 6464 cases to the preeclampsia, preeclampsia or other maternal hypertensive disorder and preeclampsia or fetal growth restriction phenotypes, respectively. Steinthorsdottir et al.[2] data includes samples from Finnish FINRISK health examination surveys that are also part of FinnGen. Therefore, we did not include any samples originating from the FINRISK study into our FinnGen preeclampsia dataset. A full list of FinnGen investigators can be found in Supplement 5.

© 2023 Tyrmi JS et al. *JAMA Cardiology.*

In EstBB the study phenotypes are based on ICD-10, ICD-9 and ICD-8 codes as described in eTable 1. EstBB is a population-based biobank, which closely reflects the age, sex and geographical distribution of the Estonian population. ICD-code information is obtained from the Estonian Causes of Death, Estonian Cancer, Estonian Tuberculosis and Estonian Health Insurance Fund registries, and also from the databases of Tartu University Hospital and North Estonia Medical Centre. The case definition for the EstBB 200K Data Freeze was similar to FinnGen, but only the ICD-10 codes were used. Total case counts in EstBB for the preeclampsia, preeclampsia or other maternal hypertensive disorder and preeclampsia or fetal growth restriction phenotypes were 1464, 4084 and 2772, respectively.

Note 3: Survival analyses between preeclampsia and other FinnGen endpoints

The goal of the analysis is to study the association between an exposure endpoint and an outcome endpoint. Associations between endpoints are calculated using the default FinnGen survival analysis workflow described below, which follows the approach described in the NB-COMO study (https://plana-ripoll.github.io/NB-COMO/).

Start of follow-up begins from date 1998-01-01, which is chosen as this date there is complete coverage for all registries. End of follow-up is defined as 2021-12-31. Prevalent cases (i.e. individuals that have been diagnosed with the outcome endpoint before 1998-01-01) were removed from the study. Only incident cases are considered. If the date of diagnoses for the exposure endpoint happens before 1998-01-01, it is assumed that it happened on 1998-01-01. Only those endpoint pairs with at least 10 individuals for each cell of the 2x2 contingency table between endpoint pairs are considered. Also, at least 25 individuals having the outcome endpoint, and endpoints must not overlap so that endpoints are not descendants of one another endpoint in the tree hierarchy or have overlapping underlying ICD codes.

To improve computational speed, a case-cohort design is used. Briefly, from the original cohort, a subcohort at the start of follow-up is selected. The subcohort can include outcome endpoints. The size of the subcohort is always 10,000 individuals randomly selected for each analysis. The final population includes all the individuals in the subcohort and all the individuals that experience the outcome endpoints outside the subcohort.

© 2023 Tyrmi JS et al. *JAMA Cardiology.*

The analysis is performed using Cox regression with a time-varying covariate, weighted by the inverse of the sampling probability to account for the case-cohort design. Robust standard error was used. The model is defined as:

Surv(time,outcome_endpoint) ~ exposure_endpoint + birth_year + sex

Time is calculated as (date end of follow-up – date entry in the study) defined in data pre-processing (except for individuals diagnosed with the exposure endpoint where time is split from entry till diagnosis and from diagnosis till the end of follow up, see below). Exposure endpoint is treated as a time-varying covariate, so that an individual is unexposed (value of the variable is set to 0) from 1998-01-01 until the diagnoses of the exposure endpoint and exposed (value of the variable is set to 1) after that. Lagged hazard ratios are computed with the following follow-up time windows: < 1 year, between 1 and 5 years, between 5 and 15 years. If an outcome endpoint occurs outside the time-widow, the individual with the disease endpoint is included in the analysis, but the outcome endpoint is not considered (i.e. variable is set to 0). The Cox regression is implemented using the lifelines library.

Bonferroni corrected p-value threshold was 0.00001.

Note 4: Genotyping and imputation

FINNPEC: Genomic DNA was extracted from whole blood using the NucleoSpin Blood XL DNA extraction kit (Macherey-Nagel GmbH & Co.) or the Chemagic Magnetic Separation Module I –machine (Chemagen) and subsequently stored at −20°C. Genotyping of the FINNPEC samples was performed using Infinium Global Screening Array-24 v2.0 BeadChip (Illumina Inc., San Diego, CA, USA) at Molecular Medicine Finland FIMM Technology Centre, University of Helsinki. Pre-imputation quality control was carried out with Plink 1.07 and 1.9. Duplicated samples and members of triads and dyads that did not show expected genetic relationships based on Mendelian errors and the IBD analysis with the Plink's –genome option were excluded. In addition, samples with unresolved sex mismatch, missingness rate over 5%, heterozygosity rate ±4 SD or non-Finnish ancestry based on MDS analysis were excluded. In the variant-wise quality control, variants with missing call rate >2%, Hardy-Weinberg equilibrium (HWE) $p<1x10^{-6}$, or minor allele count <3 were removed. The genotyped samples

© 2023 Tyrmi JS et al. *JAMA Cardiology.*

were pre-phased with Eagle v2.3.5 and imputed with Beagle v4.1 using a population-specific reference panel SISu v3, which consists of 3775 whole genome sequenced individuals of Finnish ancestry.

FinnGen: DNA isolation was completed separately in each nine Finnish biobanks that provided the samples for FinnGen project. The following DNA extraction kits were used according to the manufacturers' instructions: Chemagic DNA Blood 250 and 400 (PerkinElmer), NucleoSpin 96 Tissue Core Kit (Macherey-Nagel), QIAsymphony DSP DNA Midi Kit (QIAGEN) and sbeadex Blood DNA Purification Kit (LGC, Biosearch Technologies). Sample genotyping in FinnGen was performed using Illumina and Affymetrix arrays (Illumina Inc., San Diego, and Thermo Fisher Scientific, Santa Clara, CA, USA) at Molecular Medicine Finland FIMM Technology Centre, University of Helsinki. Genotype calls were made using GenCall or zCall for Illumina and AxiomGT1 algorithm for Affymetrix data. Genotypes with HWE p-value $p<1x10^{-6}$, minor allele count <3 and genotyping success rate <98 % were removed. Samples with ambiguous sex, high genotype missingness > 5% and those that were outliers in population structure (> 4 SD from mean on first two dimensions) were omitted. Samples were pre-phased with Eagle 2.3.5 using 20,000 conditioning haplotypes. Genotypes were imputed with Beagle 4.1 using SiSu v3 imputation reference panel.

Estonian Biobank: All EstBB participants were genotyped at the Core Genotyping Lab of the Institute of Genomics, University of Tartu, using Illumina GSAv1.0, GSAv2.0, and GSAv2.0_EST arrays. Samples were genotyped and PLINK format files were created using Illumina GenomeStudio v2.0.4. Individuals were excluded from the analysis if their call-rate was < 95% or if sex defined based on heterozygosity of X chromosome did not match sex in phenotype data. Before imputation, variants were filtered by call-rate < 95%, HWE p-value < 1e-4 (autosomal variants only), and minor allele frequency < 1%. All variants were changed to be from TOP strand using GSAMD-24v1-0_20011747_A1-b37.strand.RefAlt.zip files from https://www.well.ox.ac.uk/~wrayner/strand/ webpage. Prephasing was done using the Eagle v2.3 software (number of conditioning haplotypes Eagle2 uses when phasing each sample was set to: --Kpbwt=20000) and imputation was done using Beagle v.28Sep18.793 with effective population size ne=20,000. Population specific imputation reference of 2297 WGS samples was used[3].

Note 5: Association and meta-analysis

© 2023 Tyrmi JS et al. *JAMA Cardiology.*

To minimize spurious associations due to population structure, principal component (PC) analysis was first conducted in each cohort to define ten most significant PCs. The null models of the GWAS analyses were adjusted for maternal age at birth and the first 10 PCs (FINNPEC), age, genotyping batches and the first 10 PCs (FinnGen), or year of birth and the first 10 PCs (EstBB). FINNPEC and EstBB genotypes were available in reference genome build hg37. FinnGen summary statistics were lifted over from hg38 to hg37 reference genome build using UCSC liftOver[4] to match the other cohorts.

Genomic inflation factor estimates were calculated with 'LD Score regression' (LDSC) software[5]. Final summary statistics were then created by rerunning the meta-analysis by providing the LD Score regression intercept values for the METAL "GENOMIC CONTROL" option. Genome-wide significance was set to p-value < 5×10^{-8}. The meta-analysis was conducted by two analysts independently and summary statistics were compared for consistency. Only variants available in at least two cohorts were used for downstream analysis. Formatting and preparation of the association summary statistics data for downstream analysis was managed with the workflow management software STAPLER[6].

Note 6: Annotation of loci

We defined annotated loci as the lead variants and the surrounding +-1 Mbp area. To further narrow down the set of plausible variants, we fine-mapped each locus discovered in the three meta-analyses. We first extracted the summary statistics of each locus, and then applied the FinnGen finemapping pipeline (available at https://github.com/FINNGEN/finemapping-pipeline, accessed during 2/2/2022) with default parameters. In brief, the pipeline calculates linkage disequilibrium within the regions of interest with LDstore2[7] using FinnGen samples and generates 99 % credible sets using SuSie[8].

To identify putative candidate genes we first used the web-based FUMA platform[9], which is intended for functionally annotating GWAS findings to prioritize most likely causal variants and genes via using information stored in biological data repositories and tools. Using the FUMA results, we examined whether the variants residing in the associating loci had any predicted functional consequences by identifying missense mutations or pathogenic variants, whether the variants were eQTLs based on the information provided in the Genotype-Tissue Expression (GTEx) Portal or had been previously associated with any preeclampsia related phenotype in

© 2023 Tyrmi JS et al. *JAMA Cardiology.*

the GWAS catalog[10]. In addition, we assessed if the variants had high CADD score[11]. ANNOVAR[12] and MAGMA[13] are integrated as part of FUMA, and were used for performing gene-based analysis and creating functional annotation for the meta-analysis results, respectively. In the MAGMA gene-based analysis multiple genetic markers are analyzed simultaneously to determine their joint effect, which is in contrast with GWAS analysis where the association of each individual variant with the studied phenotype is tested separately. In the absence of functional evidence, the genes most proximal to the lead variant in each locus were prioritized.

Central approach for identifying putative causal genes and obtaining novel understanding of underlying biology was our careful manual curation of the biological function of the functionally implicated or proximal genes in each associated loci via conducting literature and GWAS catalogue searches and examining GenBank[14] and UniProt[15] databases.

Note 7: Genetic correlations

To further describe the correlation of the findings with other disorders – possibly not described in earlier literature – we conducted a PheWAS analysis for the lead variants using 2,861 phenotypes provided in FinnGen Release 6. In this analysis significance threshold was adjusted via Bonferroni correction (0.05/2,861) to p-value $1.75*10^{-5}$.

We used the LDSC software[5,16] to estimate regression-derived SNP-based heritability and to evaluate genetic correlation (r_g) between preeclampsia, preeclampsia or other maternal hypertensive disorder and preeclampsia or fetal growth restriction and 894 other previously published phenotypes (eTable 6). The phenotypes were selected to closely match the (currently defunct) LDhub[17] dataset, combined with additional circulating metabolite data from Kettunen et al.[18]. Significance threshold was adjusted using Bonferroni correction (0.05/894) to p-value $< 5.6*10^{-5}$.

Finally, we calculated polygenic risk scores (PRS) by first running our meta-analyses of the two phenotypes without the FINNPEC cohort. The results were then used to generate PRS weights using PRS-CS[19] as implemented in the FinnGen PRS-pipeline (https://github.com/FINNGEN/CS-PRS-pipeline). We used default parameters of the pipeline and a European linkage disequilibrium reference panel with 1.1 million variants derived from samples from the 1000 Genomes Project[20]. The obtained weights were then applied to the variants

© 2023 Tyrmi JS et al. *JAMA Cardiology.*

of FINNPEC cohort samples. To assess the effect of PRS on the risk of preeclampsia, we defined two PRS groups: top decile and bottom 90 % group as a reference. We compared the women with high PRS with the reference group using logistic regression. The regression model included age, parity, BMI, and the blood pressure of the first trimester. We also studied the change of Nagelkerke's R^2 values to assess the change in goodness of fit of different models when PRS information was incorporated to known risk factors of preeclampsia: age, parity, BMI and blood pressure of the first trimester.

Note 8: Polygenic risk scores (PRS)

FINNPEC has extensive clinical information on studied pregnancies, and was therefore selected for the target cohort for calculating PRS. Regression model comparing the top 10 % preeclampsia-PRS compared to bottom 90 % showed statistically significant association with preeclampsia and with preeclampsia with severe symptoms as shown in eTables 8-10. Similar association was shown between PRS of preeclampsia or other maternal hypertensive and both preeclampsia, preeclampsia with severe features and the combinatory phenotype of preeclampsia or other maternal hypertensive disorder. In addition, we tested whether similar associations could be seen using PRS developed for systolic blood pressure, as in earlier studies[56,57]. Our results show no statistical association, possibly due to incorporation of first trimester systolic blood pressure measurement to the model. Adding the PRS information to the preeclampsia and to the preeclampsia with severe symptoms statistical models led to improvements of Nagelkerke's R^2 by 9.4 % and 6.1 %, respectively. Inclusion of preeclampsia or other maternal hypertensive PRS instead of preeclampsia PRS improved the R^2 of the preeclampsia model 10.2 %. The R^2 of preeclampsia or other maternal hypertensive disorder improved 8.2 % by including PRS for the preeclampsia or other maternal hypertensive disorder phenotype.

It should be noted that FINNPEC preeclampsia patients were recruited from Finnish University hospitals and may therefore include cases, which represent more severe symptoms than the general obstetric population. Due to being a case-control cohort, FINNPEC contains a larger proportion of cases compared to the other cohorts used in this work. Therefore the odds ratios and the improvement in Nagelkerke's R^2 values we report may be inflated, although similar associations to ours have been demonstrated in earlier studies of preeclampsia and various PRSs[56,57].

Note 9: Literature lookup of genes at novel identified risk loci

© 2023 Tyrmi JS et al. *JAMA Cardiology.*

Note 9.1. *PGR* mediates the effects of progesterone, an essential hormone in pregnancy that maintains pregnancy and promotes endometrial maturation, angiogenesis, vasodilation and placentation[34]. Transcriptomic profiling has revealed perturbed PGR signaling in the decidual endometrium of previously preeclamptic women[35]. Mutations in the other plausible candidate gene in the same locus, *TRPC6,* have impact on various biochemical pathways throughout the body. Variants in this gene have been shown to cause familial focal segmental glomerulosclerosis[36], and to affect vascular smooth muscle contractility[37], which may subsequently contribute to the risk of cardiac hypertrophy, heart failure[38] and idiopathic pulmonary arterial hypertension[39]. Also, the gene exhibits enhanced placental expression, and the *TRPC6* knock-out mice present with structural changes of the placenta and reduced litter sizes[40].

PZP is a protease inhibitor that prevents the activity of all four classes of proteases and it stabilises misfolded proteins, which have been shown to accumulate in preeclampsia and contribute to its pathophysiology[41–45]. For instance, *PZP* has been shown to inhibit the aggregation of amyloid beta peptide[46] – an important driver in plaque formation in several disorders, including preeclampsia, age-related macular degeneration (particularly in women), and Alzheimer disease. Also importantly, the protein has been suggested to clear out pro-inflammatory cytokines and inhibit the effects of T helper cells, thus preventing further inflammation, oxidative stress and placental dysfuntion[47]. *PZP* is also highly expressed in late-pregnancy serum and its upregulation during pregnancy represents a major maternal adaptation that helps to maintain extracellular proteostasis during gestation[46]. Furthermore, balanced expression of Glycodelin A (GdA) and its carrier protein *PZP* in the decidua seems crucial for a successful ongoing pregnancy[48].

The *ACTN4* gene encodes for an actin-binding protein with multiple roles in different cell types, and is well known for causing focal segmental glomerulosclerosis[49–51]. The gene product is involved in the maintenance of cytoskeletal structure, modulating cell motility and regulating endothelial cells[52,53], and may play a role in placentation[54]. *ACTN4* has been suspected to act as a regulator of trophoblast proliferation and differentiation during early pregnancy, and has been shown to have reduced expression in severe preeclampsia[54]. Specifically, the lack of expression appears to prevent cytotropboblast differentiation into interstitial extravillous cytotrophoblasts, possibly compromising the remodelling of the spiral arteries. In addition, the reduced *ACTN4* levels have been suggested to activate endothelial cell apoptosis[55]. In laboratory studies conducted by Kos et al.[51], *ACTN4*$^{-/-}$ mice developed a severe glomerular disease with no other histological abnormalities. However,

only 6 % of the offspring of mated *ACTN4*$^{+/-}$ mice were homozygous for the dysfunctional gene, instead of the expected 25 %. Whether the reduced litter size observed in this study was caused by dysfunctional placental development remains unclear since the placentas were unfortunately not examined. Further functional studies are needed to gain evidence of the potential causal role of *ACTN4* in preeclampsia and to uncover pathophysiological mechanisms behind the association of this locus.

Note 9.2. In pregnancy, HLA-G, -F, -E and -C are potentially directly involved in tolerance induction by recognition of fetal trophoblast cells by maternal immune cells, and the classical HLA genes likely also play a role in the maintenance of peripheral tolerance[21–23]. The association lies within the major susceptibility locus for psoriasis (PSORS1), known for accounting much of the genetic risk for psoriasis[24,25]. The two top outliers of the HLA-region in MAGMA test, 'Coiled-Coil Alpha-Helical Rod Protein' (*CCHCR1*) and 'Psoriasis Susceptibility 1 Candidate 2' (*PSORS1C2*), have indeed been previously suggested as risk genes for psoriasis[26–28]. Additionally, evidence shows *PSORS1C2* effects on apolipoprotein B and blood protein levels[29,30]. Psoriasis increases the risk of adverse pregnancy outcomes and specifically preeclampsia with an odds ratio of 1.5[30–33].

Note 10: Study of preeclampsia or indication of fetal growth restriction.

As fetal growth restriction is a common symptom of preeclampsia, we also studied the combinatory phenotype defined as preeclampsia or fetal growth restriction (as mother's diagnosis). The available sample sizes are shown in eTable S11. The analysis workflow was similar to the preeclampsia and preeclampsia or other maternal hypertensive disorder and preeclampsia phenotypes described in the main text and eAppendix Notes 1-2. Workflow of the preeclampsia or fetal growth restriction is visualized in eFigure 6.

Preliminary meta-analysis results showed evidence of slight inflation in the meta-analysis test statistics based on the calculated LD Score intercept 1.0147 (SE 0.0077). This inflation was then corrected by rerunning the meta-analysis with genomic correction**.** Meta-analysis uncovered two loci (eTable 12, eFigures 7-9). The novel locus at 9q22 detected in meta-analysis of the preeclampsia phenotype was also detected when analyzing preeclampsia or fetal growth restriction phenotype, although the lead variant here was rs7470773 instead of the rs7862828 in preeclampsia. Interestingly, the lead variant in the meta-analysis of preeclampsia or fetal growth restriction is in only modest LD (r^2 0.34) with the preeclampsia meta-analysis lead variant, suggesting that the mechanisms behind the association might differ between the two phenotypes. The preeclampsia or fetal growth

© 2023 Tyrmi JS et al. *JAMA Cardiology.*

restriction lead variant is in high LD (r^2 0.98) with rs7028982 (p-value 6.7e-09, beta 0.13), and the risk allele of rs7028982 reduces the expression of 'Nuclear Factor Interleukin 3 Regulated' (*NFIL3)* in whole blood (normalized effect size -0.14) according to the GTEx database. In mice studies, the dysfunction of *NFIL3* on 9q22 has been shown to cause absence of uterine natural killer cells[58], which have a central role in the placental vascular remodeling during pregnancy[59]. This absence results in incomplete remodeling of the uterine arteries and decidua, placental defects, and fetal growth restriction[58], which are all features associated with preeclampsia.

© 2023 Tyrmi JS et al. *JAMA Cardiology.*

References

1. Pihkala J, Hakala T, Voutilainen P, Raivio K. Uudet suomalaiset sikiön kasvukäyrät. *Duodecim*. 1989;105(18):1540-1546.
2. Steinthorsdottir V, McGinnis R, Williams NO, et al. Genetic predisposition to hypertension is associated with preeclampsia in European and Central Asian women. *Nat Commun*. 2020;11(1). doi:10.1038/s41467-020-19733-6
3. Mitt M, Kals M, Pärn K, et al. Improved imputation accuracy of rare and low-frequency variants using population-specific high-coverage WGS-based imputation reference panel. *Eur J Hum Genet*. 2017;25(7):869-876. doi:10.1038/ejhg.2017.51
4. Kent WJ, Sugnet CW, Furey TS, et al. The Human Genome Browser at UCSC. *Genome Res*. 2002;12(6):996-1006. doi:10.1101/gr.229102
5. Bulik-Sullivan B, Loh PR, Finucane HK, et al. LD score regression distinguishes confounding from polygenicity in genome-wide association studies. *Nat Genet*. 2015;47(3):291-295. doi:10.1038/ng.3211
6. Tyrmi JS. STAPLER: a simple tool for creating, managing and parallelizing common high-throughput sequencing workflows. *bioRxiv*. Published online January 1, 2018:445056. doi:10.1101/445056
7. Benner C, Havulinna AS, Järvelin MR, Salomaa V, Ripatti S, Pirinen M. Prospects of Fine-Mapping Trait-Associated Genomic Regions by Using Summary Statistics from Genome-wide Association Studies. *Am J Hum Genet*. 2017;101(4):539-551. doi:10.1016/j.ajhg.2017.08.012
8. Wang G, Sarkar A, Carbonetto P, Stephens M. A simple new approach to variable selection in regression, with application to genetic fine mapping. *J R Stat Soc Ser B Stat Methodol*. 2020;82(5):1273-1300. doi:10.1111/rssb.12388
9. Watanabe K, Taskesen E, Van Bochoven A, Posthuma D. Functional mapping and annotation of genetic associations with FUMA. *Nat Commun*. 2017;8(1):1-10. doi:10.1038/s41467-017-01261-5
10. Buniello A, Macarthur JAL, Cerezo M, et al. The NHGRI-EBI GWAS Catalog of published genome-wide association studies, targeted arrays and summary statistics 2019. *Nucleic Acids Res*. 2019;47(D1):D1005-D1012. doi:10.1093/nar/gky1120
11. Kircher M, Witten DM, Jain P, O'Roak BJ, Cooper GM, Shendure J. A general

© 2023 Tyrmi JS et al. *JAMA Cardiology.*

framework for estimating the relative pathogenicity of human genetic variants. *Nat Genet*. 2014;46(3):310-315. doi:10.1038/ng.2892
12. Wang K, Li M, Hakonarson H. ANNOVAR: Functional annotation of genetic variants from high-throughput sequencing data. *Nucleic Acids Res*. 2010;38(16):1-7. doi:10.1093/nar/gkq603
13. de Leeuw CA, Mooij JM, Heskes T, Posthuma D. MAGMA: Generalized Gene-Set Analysis of GWAS Data. *PLoS Comput Biol*. 2015;11(4):1-19. doi:10.1371/journal.pcbi.1004219
14. Sayers EW, Cavanaugh M, Clark K, et al. GenBank. *Nucleic Acids Res*. 2021;49(D1):D92-D96. doi:10.1093/nar/gkaa1023
15. Bateman A. UniProt: A worldwide hub of protein knowledge. *Nucleic Acids Res*. 2019;47(D1):D506-D515. doi:10.1093/nar/gky1049
16. Bulik-Sullivan B, Finucane HK, Anttila V, et al. An atlas of genetic correlations across human diseases and traits. *Nat Genet*. 2015;47(11):1236-1241. doi:10.1038/ng.3406
17. Zheng J, Erzurumluoglu AM, Elsworth BL, et al. LD Hub: A centralized database and web interface to perform LD score regression that maximizes the potential of summary level GWAS data for SNP heritability and genetic correlation analysis. *Bioinformatics*. 2017;33(2):272-279. doi:10.1093/bioinformatics/btw613
18. Kettunen J, Demirkan A, Würtz P, et al. Genome-wide study for circulating metabolites identifies 62 loci and reveals novel systemic effects of LPA. *Nat Commun*. 2016;7:1-9. doi:10.1038/ncomms11122
19. Ge T, Chen CY, Ni Y, Feng YCA, Smoller JW. Polygenic prediction via Bayesian regression and continuous shrinkage priors. *Nat Commun*. 2019;10(1):1-10. doi:10.1038/s41467-019-09718-5
20. Consortium 1000 Genomes Project, others. A global reference for human genetic variation. *Nature*. 2015;526(7571):68.
21. Lokki AI, Heikkinen-Eloranta JK, Laivuori H. The immunogenetic conundrum of preeclampsia. *Front Immunol*. 2018;9(NOV):1-8. doi:10.3389/fimmu.2018.02630
22. Ranella A, Vassiliadis S, Mastora C, Valentina M, Dionyssopoulou E, Athanassakis I. Constitutive intracellular expression of human leukocyte antigen (HLA)-DO and HLA-DR but not HLA-DM in trophoblast cells. *Hum*

© 2023 Tyrmi JS et al. *JAMA Cardiology.*

Immunol. 2005;66(1):43-55. doi:10.1016/j.humimm.2004.10.002
23. Wedenoja S, Yoshihara M, Teder H, et al. Fetal HLA-G mediated immune tolerance and interferon response in preeclampsia. *EBioMedicine*. 2020;59:102872. doi:10.1016/j.ebiom.2020.102872
24. Nair RP, Henseler T, Jenisch S, et al. Evidence for two psoriasis susceptibility loci (HLA and 17q) and two novel candidate regions (16q and 20p) by genome-wide scan. *Hum Mol Genet*. 1997;6(8):1349-1356. doi:10.1093/hmg/6.8.1349
25. Trembath RC, Clough RL, Rosbotham JL, et al. Identification of a major susceptibility locus on chromosome 6p and evidence for further disease loci revealed by a two stage genome-wide search in psoriasis. *Hum Mol Genet*. 1997;6(5):813-820. doi:10.1093/hmg/6.5.813
26. Holm SJ, Carlén LM, Mallbris L, Ståhle-Bäckdahl M, O'Brien KP. Polymorphisms in the SEEK1 and SPR1 genes on 6p21.3 associate with psoriasis in the Swedish population. *Exp Dermatol*. 2003;12(4):435-444. doi:10.1034/j.1600-0625.2003.00048.x
27. Asumalahti K, Laitinen T, Itkonen-Vatjus R, et al. A candidate gene for psoriasis near HLA-C, HCR (Pg8), is highly polymorphic with a disease-associated susceptibility allele. *Hum Mol Genet*. 2000;9(10):1533-1542. doi:10.1093/hmg/9.10.1533
28. Asumalahti K, Veal C, Laitinen T, et al. Coding haplotype analysis supports HCR as the putative susceptibility gene for psoriasis at the MHC PSORS1 locus. *Hum Mol Genet*. 2002;11(5):589-597. doi:10.1093/hmg/11.5.589
29. Sun W, Kechris K, Jacobson S, et al. Common Genetic Polymorphisms Influence Blood Biomarker Measurements in COPD. *PLoS Genet*. 2016;12(8):1-33. doi:10.1371/journal.pgen.1006011
30. Serrano NC, Guio-Mahecha E, Quintero-Lesmes DC, et al. Lipid profile, plasma apolipoproteins, and pre-eclampsia risk in the GenPE case-control study. *Atherosclerosis*. 2018;276:189-194. doi:10.1016/j.atherosclerosis.2018.05.051
31. Huang YH, Yee Ng C, Chiou MJ, Kuo CF. Fetal–neonatal and maternal outcomes in women with psoriasis vulgaris: A nationwide population-based registry linkage study in Taiwan. *J Dermatol*. 2021;48(2):184-189. doi:10.1111/1346-8138.15658

© 2023 Tyrmi JS et al. *JAMA Cardiology.*

32. Yang YW, Chen CS, Chen YH, Lin HC. Psoriasis and pregnancy outcomes: A nationwide population-based study. *J Am Acad Dermatol*. 2011;64(1):71-77. doi:10.1016/j.jaad.2010.02.005
33. Bröms G, Haerskjold A, Granath F, Kieler H, Pedersen L, Berglind IA. Effect of maternal psoriasis on pregnancy and birth outcomes: A population-based cohort study from Denmark and Sweden. *Acta Derm Venereol*. 2018;98(8):728-734. doi:10.2340/00015555-2923
34. Pereira MM, Mainigi M, Strauss JF. Secretory products of the corpus luteum and preeclampsia. *Hum Reprod Update*. 2021;27(4):651-672. doi:10.1093/humupd/dmab003
35. Garrido-Gomez T, Castillo-Marco N, Clemente-Ciscar M, et al. Disrupted pgr-b and esr1 signaling underlies defective decidualization linked to severe preeclampsia. *Elife*. 2021;10:1-20. doi:10.7554/eLife.70753
36. Winn MP, Conlon PJ, Lynn KL, et al. Medicine: A mutation in the TRPC6 cation channel causes familial focal segmental glomerulosclerosis. *Science (80-)*. 2005;308(5729):1801-1804. doi:10.1126/science.1106215
37. Dietrich A, Mederos y Schnitzler M, Gollasch M, et al. Increased Vascular Smooth Muscle Contractility in TRPC6 −/− Mice . *Mol Cell Biol*. 2005;25(24):11191-11191. doi:10.1128/mcb.25.24.11191.2005
38. Yamaguchi Y, Iribe G, Nishida M, Naruse K. Role of TRPC3 and TRPC6 channels in the myocardial response to stretch: Linking physiology and pathophysiology. *Prog Biophys Mol Biol*. 2017;130:264-272. doi:10.1016/j.pbiomolbio.2017.06.010
39. Yu Y, Keller SH, Remillard C V., et al. A functional single-nucleotide polymorphism in the TRPC6 gene promoter associated with idiopathic pulmonary arterial hypertension. *Circulation*. 2009;119(17):2313-2322. doi:10.1161/CIRCULATIONAHA.108.782458
40. Hasna J, Nahed RA, Sergent F, Alfaidy N, Bouron A. The deletion of TRPC6 channels perturbs iron and zinc homeostasis and pregnancy outcome in mice. *Cell Physiol Biochem*. 2019;52(3):455-467. doi:10.33594/000000033
41. Buhimschi IA, Nayeri UA, Zhao G, et al. Protein misfolding, congophilia, oligomerization, and defective amyloid processing in preeclampsia. *Sci Transl Med*. 2014;6(245):245ra92. doi:10.1126/scitranslmed.3008808
42. Buhimschi IA, Zhao G, Funai EF, et al. Proteomic profiling of urine identifies

© 2023 Tyrmi JS et al. *JAMA Cardiology.*

specific fragments of SERPINA1 and albumin as biomarkers of preeclampsia. *Am J Obstet Gynecol*. 2008;199(5):551.e1-551.e16. doi:10.1016/j.ajog.2008.07.006

43. Tong M, Cheng S Bin, Chen Q, et al. Aggregated transthyretin is specifically packaged into placental nano-vesicles in preeclampsia. *Sci Rep*. 2017;7(1):1-9. doi:10.1038/s41598-017-07017-x
44. Kalkunte SS, Neubeck S, Norris WE, et al. Transthyretin is dysregulated in preeclampsia, and its native form prevents the onset of disease in a preclinical mouse model. *Am J Pathol*. 2013;183(5):1425-1436. doi:10.1016/j.ajpath.2013.07.022
45. Millen KR, Buhimschi CS, Zhao G, Rood KM, Tabbah S, Buhimschi IA. Serum and Urine Thioflavin-T-Enhanced Fluorescence in Severe Preeclampsia. *Hypertens (Dallas, Tex 1979)*. 2018;71(6):1185-1192. doi:10.1161/HYPERTENSIONAHA.118.11034
46. Cater JH, Kumita JR, Abdallah RZ, et al. Human pregnancy zone protein stabilizes misfolded proteins including preeclampsia- and Alzheimer'sassociated amyloid beta peptide. *Proc Natl Acad Sci U S A*. 2019;116(13):6101-6110. doi:10.1073/pnas.1817298116
47. Wyatt AR, Cater JH, Ranson M. PZP and PAI-2: Structurally-diverse, functionally similar pregnancy proteins? *Int J Biochem Cell Biol*. 2016;79:113-117. doi:10.1016/j.biocel.2016.08.018
48. Löb S, Vattai A, Kuhn C, et al. Pregnancy Zone Protein (PZP) is significantly upregulated in the decidua of recurrent and spontaneous miscarriage and negatively correlated to Glycodelin A (GdA). *J Reprod Immunol*. 2021;143(August 2020). doi:10.1016/j.jri.2020.103267
49. Pollak MR. Inherited podocytopathies: FSGS and nephrotic syndrome from a genetic viewpoint. *J Am Soc Nephrol*. 2002;13(12):3016-3023. doi:10.1097/01.ASN.0000039569.34360.5E
50. Kaplan JM, Kim SH, North KN, et al. Mutations in ACTN4, encoding α-actinin-4, cause familial focal segmental glomerulosclerosis. *Nat Genet*. 2000;24(3):251-256. doi:10.1038/73456
51. Kos CH, Le TC, Sinha S, et al. Mice deficient in α-actinin-4 have severe glomerular disease. *J Clin Invest*. 2003;111(11):1683-1690. doi:10.1172/jci200317988

© 2023 Tyrmi JS et al. *JAMA Cardiology.*

52. Sjöblom B, Salmazo A, Djinović-Carugo K. α-Actinin structure and regulation. *Cell Mol Life Sci*. 2008;65(17):2688-2701. doi:10.1007/s00018-008-8080-8
53. Hiroi Y, Guo Z, Li Y, Beggs AH, Liao JK. Dynamic regulation of endothelial NOS mediated by competitive interaction with alpha-actinin-4 and calmodulin. *FASEB J Off Publ Fed Am Soc Exp Biol*. 2008;22(5):1450-1457. doi:10.1096/fj.07-9309com
54. Peng W, Liu Y, Qi H, Li Q. Alpha-actinin-4 is essential for maintaining normal trophoblast proliferation and differentiation during early pregnancy. *Reprod Biol Endocrinol*. 2021;19(1):1-12. doi:10.1186/s12958-021-00733-0
55. Zhao J, Peng W, Ran Y, et al. Dysregulated expression of ACTN4 contributes to endothelial cell injury via the activation of the p38-MAPK/p53 apoptosis pathway in preeclampsia. *J Physiol Biochem*. 2019;75(4):475-487. doi:10.1007/s13105-019-00700-9
56. Kivioja A, Toivonen E, Tyrmi J, et al. Increased Risk of Preeclampsia in Women With a Genetic Predisposition to Elevated Blood Pressure. *Hypertension*. 2022;79(9):2008-2015. doi:10.1161/HYPERTENSIONAHA.122.18996
57. Nurkkala J, Kauko A, FinnGen, et al. Associations of polygenic risk scores for preeclampsia and blood pressure with hypertensive disorders of pregnancy. *J Hypertens*. 2022;Publish Ah(3). doi:10.1097/hjh.0000000000003336
58. Boulenouar S, Doisne J-M, Sferruzzi-Perri A, et al. The Residual Innate Lymphoid Cells in NFIL3-Deficient Mice Support Suboptimal Maternal Adaptations to Pregnancy. *Front Immunol*. 2016;7:43. doi:10.3389/fimmu.2016.00043
59. Sojka DK, Yang L, Yokoyama WM. Uterine natural killer cells. *Front Immunol*. 2019;10(MAY):1-9. doi:10.3389/fimmu.2019.00960

© 2023 Tyrmi JS et al. *JAMA Cardiology.*

eTable 1. International Classification of Diseases and Related Health Problems (ICD) codes for the FinnGen and Estonian biobank (only ICD-10 in the case of Estonian biobank) phenotypes of hypertensive pregnancy.

Phenotype	ICD-10	ICD-9	ICD-8
PE	O11, O14.0, O14.1, O14.9, O15.0, O15.1, O15.2	6424, 6425, 6426, 6427	63703, 63704, 63709, 63710, 63799, 66120
PE-HTP	O10, O11, O13, O14, 015, O16	642	63701, 63703, 63704, 63709, 63710, 63799, 66120
PE-FGR	O11, O14.0, O14.1, O14.9, O15.0, O15.1, O15.2, O36.5	6424, 6425, 6426, 6427, 6565A, 6565B	63703, 63704, 63709, 63710, 63799, 66120

PE = preeclampsia, PE-HTP = preeclampsia or other maternal hypertensive disorder , PE-FGR = preeclampsia or fetal growth restriction. Control groups for each of the phenotypes consisted of parous women with no case ICD codes for the phenotype in question.

eTable 2. Age and BMI of the cases of the three phenotypes in each study cohort. In FinnGen and EstBB the age refers to the first diagnosis. In FINNPEC the age refers to the index pregnancy, which is not always the first occurrence of the study phenotype.

Cohort	Phenotype	Age at diagnosis (mean, SD)	BMI (median, IQR)
FinnGen	PE	28.7 (5.6)	27.9 (8.3)
	PE-HTP	29.5 (5.8)	28 (8.5)
	PE-FGR	29.1 (5.7)	27.1 (8.3)
EstBB	PE	29.7 (7.0)	25.1 (7.6)
	PE-HTP	30.2 (7.2)	26.0 (8.5)
	PE-FGR	29.4 (6.4)	23.4 (6.9)
FINNPEC	PE	30.3 (5.5)	24.0 (6.3)
	PE-HTP	30.3 (5.5)	24.0 (6.4)
	PE-FGR	30.3 (5.5)	24.0 (6.5)

PE = preeclampsia, PE-HTP = preeclampsia or other maternal hypertensive disorder, PE-FGR = preeclampsia or fetal growth restriction, SD = standard deviation, IQR = interquartile range.

© 2023 Tyrmi JS et al. *JAMA Cardiology.*

eTable 3. Additional clinical variables available in FINNPEC.

Phenotype		Age at diagnosis (mean, SD)	BMI (median,IQR)	Gestational diabetes (%)	Preterm birth (%)	1st trimester SBP mmHg (median,IQR)	1st trimester DBP mmHg (median,IQR)	Birth weight (median,IQR)	Parous (%)
PE	Cases	30.3 (5.5)	24.0 (6.3)	11.7	38.2	124 (16)	78 (14)	2770 (1235)	25.4
	Controls	29.7 (5.2)	23.1 (5.2)	9.0	6.4	119 (13)	73 (12)	3520 (750)	42.6
PE-HTP	Cases	30.3 (5.5)	24.0 (6.4)	12.1	35.5	124 (18)	78 (13)	2830 (1270)	27.0
	Controls	29.3 (5.1)	22.8 (4.9)	7.6	4.0	117 (15)	71 (11)	3580 (670)	45.1
PE-FGR	Cases	30.3 (5.5)	24.0 (6.5)	12.0	35.6	124 (17)	78 (13)	2815 (1270)	27.2
	Controls	29.4 (5.1)	22.9 (5.0)	7.7	2.6	117 (15)	71 (11)	2605 (629)	45.4

PE = preeclampsia, PE-HTP = preeclampsia or other maternal hypertensive disorder, PE-FGR = preeclampsia or fetal growth restriction, SBP = systolic blood pressure, mmHG = millimetres of mercury, SD = standard deviation, IQR = interquartile range.

© 2023 Tyrmi JS et al. *JAMA Cardiology*.

eTable 5. Survival analyses between preeclampsia and all other FinnGen disease endpoints. Only significant associations are shown. FinnGen disease endpoint catalog includes a large number of near-identical subtypes for some highly studied diseases. In cases where several near-identical subtypes reached statistical significance in our survival analysis results, we omitted the subtypes and only retained the most general endpoint available for clarity.

	After preeclampsia diagnosis		
**Endpoint**	**HR [95 % CI]**	**P-value**	**N**
**Gestational [pregnancy-induced] hypertension**	17.3[14.18, 21.10]	<1e-100	820
**Failed induction of labour**	14.68[11.56, 18.64]	<1e-100	177
**Gestational [pregnancy-induced] oedema and proteinuria without hypertension**	12.49[9.81, 15.91]	5.80E-93	149
**Premature separation of placenta [abruptio placentae]**	9.88[7.00, 13.96]	1.10E-38	47
**Pre-existing hypertension complicating pregnancy, childbirth and the puerperium**	8.65[7.03, 10.64]	3.40E-92	224
**Multiple delivery**	8.4[6.67, 10.58]	2.60E-73	144
**Poor fetal growth**	8.17[6.67, 10.02]	5.60E-91	300
**Complications of the puerperium, not elsewhere classified**	7.53[5.58, 10.15]	5.80E-40	68
**Postpartum haemorrhage**	7.29[5.98, 8.90]	2.40E-85	400
**Complications predominantly related to the puerperium**	7.04[5.73, 8.65]	2.00E-77	320
**Other puerperal infections**	7.04[5.58, 8.88]	3.00E-61	160
**Other assisted single delivery**	6.86[4.66, 10.12]	2.20E-22	34
**Labour and delivery complicated by fetal stress [distress]**	6.85[5.64, 8.31]	1.70E-84	606
**Single spontaneous delivery**	6.78[5.43, 8.45]	1.70E-64	1543
**Postpartum haemorrhage due to anatomy**	6.62[5.27, 8.33]	1.10E-58	172
**Maternal care for other known or suspected fetal problems**	6.48[5.34, 7.85]	5.40E-81	631
**Labour and delivery complicated by intrapartum haemorrhage, not elsewhere classified**	5.9[3.83, 9.10]	8.90E-16	26
**Other obstructed labour**	5.81[4.30, 7.85]	2.00E-30	66
**Long labour**	5.75[4.69, 7.04]	6.10E-64	334
**Infections of genitourinary tract in pregnancy**	5.73[4.28, 7.67]	1.10E-31	77
**Labour and delivery complicated by umbilical cord complications**	5.72[4.05, 8.08]	4.60E-23	45
**Postpartum haemorrhage due to retained placenta**	5.64[4.45, 7.16]	5.00E-46	137
**Other complications of labour and delivery, not elsewhere classified**	5.63[4.44, 7.14]	3.00E-46	155
**Retained placenta and membranes, without haemorrhage**	5.28[4.04, 6.90]	2.80E-34	91
**Other disorders of amniotic fluid and membranes**	5.16[4.17, 6.40]	7.90E-51	208
**Maternal care for other conditions predominantly related to pregnancy**	5.09[4.10, 6.32]	3.30E-49	241
**Intrahepatic Cholestasis of Pregnancy (ICP)**	5.07[3.88, 6.63]	1.90E-32	91
**Maternal care related to the fetus and amniotic cavity and possible delivery problems**	5.06[4.15, 6.16]	2.20E-58	1286
**Other obstetric trauma**	4.86[3.63, 6.50]	1.50E-26	69
**Perineal laceration during delivery**	4.77[3.84, 5.91]	7.70E-46	219
**Type 1 diabetes with renal complications**	4.64[3.48, 6.20]	2.10E-25	72

© 2023 Tyrmi JS et al. *JAMA Cardiology.*

Endpoint	After preeclampsia diagnosis HR [95 % CI]	P-value	N
**Gestational diabetes (for exclusion)**	4.52[3.75, 5.46]	1.10E-55	568
**Abnormal findings on antenatal screening of mother**	4.28[3.28, 5.58]	8.40E-27	101
**Polyhydramnios**	4.28[3.13, 5.86]	8.60E-20	57
**Puerperal sepsis**	4.2[3.08, 5.74]	1.70E-19	56
**Obstructed labour due to malposition and malpresentation of fetus**	4.15[3.37, 5.11]	2.20E-41	232
**Hyperkinetic disorders (more control exclusions)**	3.91[2.81, 5.44]	6.00E-16	57
**Disturbance of activity and attention**	3.88[2.80, 5.38]	3.30E-16	57
**Single delivery by forceps and vacuum extractor**	3.85[3.17, 4.67]	7.50E-43	361
**Other maternal disorders predominantly related to pregnancy**	3.72[3.06, 4.52]	6.60E-40	761
**Other noninflammatory disorders of uterus, except cervix**	3.72[2.97, 4.66]	3.60E-30	184
**Other maternal diseases classifiable elsewhere but complicating pregnancy, childbirth and the puerperium**	3.69[3.07, 4.44]	1.50E-43	527
**Type 1 diabetes with neurological complications**	3.69[2.61, 5.21]	1.50E-13	44
**Menorrhagia**	3.66[3.08, 4.35]	2.10E-49	655
**Antepartum haemorrhage, not elsewhere classified**	3.59[2.78, 4.65]	1.50E-22	102
**Excessive, frequent and irregular menstruation**	3.58[3.05, 4.19]	1.50E-55	1265
**Abnormalities of forces of labour**	3.48[2.61, 4.65]	2.50E-17	71
**Hypertrophy of breast**	3.43[2.80, 4.19]	3.10E-33	264
**Diabetic nephropathy (more control exclusions)**	3.4[2.63, 4.40]	1.30E-20	110
**Maternal care for known or suspected malpresentation of fetus**	3.37[2.74, 4.15]	3.00E-30	253
**Inflammatory disease of uterus**	3.31[2.64, 4.16]	4.00E-25	148
**Diabetic neuropathy**	3.31[2.48, 4.41]	3.90E-16	77
**Type 2 diabetes with ophthalmic complications**	3.29[2.54, 4.25]	9.70E-20	125
**Obstructed labour due to maternal pelvic abnormality**	3.25[2.33, 4.52]	2.80E-12	47
**Excessive vomiting in pregnancy**	3.05[2.20, 4.24]	3.00E-11	50
**Polycystic ovarian syndrome, consortium definition**	3.02[2.55, 3.59]	3.10E-37	686
**Irregular menses**	2.97[2.51, 3.51]	3.40E-37	690
**Premature rupture of membranes**	2.96[2.40, 3.65]	6.40E-24	218
**Type 1 diabetes with ophthalmic complications**	2.96[2.38, 3.69]	1.60E-22	171
**Pregnancy with abortive outcome**	2.93[2.40, 3.58]	3.70E-26	617
**Glomerular diseases**	2.93[2.31, 3.70]	5.10E-19	139
**Leiomyoma of uterus**	2.91[2.44, 3.48]	5.60E-32	773
**Maternal care for known or suspected fetal abnormality and damage**	2.89[2.32, 3.59]	1.70E-21	182
**Ovarian cyst**	2.88[2.42, 3.43]	3.60E-32	674
**Secondary hypertension**	2.85[2.19, 3.70]	5.80E-15	93
**Other abnormal uterine and caginal bleeding**	2.84[2.23, 3.62]	3.40E-17	111
**Noninflammatory disorders of female genital tract**	2.77[2.36, 3.25]	2.20E-35	2615
**Spontaneous abortion**	2.71[2.21, 3.33]	1.00E-21	275
**Maternal care for known or suspected disproportion**	2.7[1.94, 3.76]	4.20E-09	47
**Glomerulonephritis**	2.64[1.95, 3.58]	3.90E-10	61

© 2023 Tyrmi JS et al. *JAMA Cardiology.*

Endpoint	After preeclampsia diagnosis		
	HR [95 % CI]	P-value	N
Other disorders of breast	2.61[2.03, 3.36]	6.80E-14	112
Inflammatory diseases of female pelvic organs	2.55[2.12, 3.07]	2.40E-23	480
Other disorders of glucose regulation and pancreatic internal secretion	2.55[1.87, 3.48]	3.60E-09	60
False labour	2.54[2.10, 3.08]	7.40E-22	412
Unspecified/other endometriosis	2.52[1.92, 3.31]	2.30E-11	80
Ovarian dysfunction	2.49[1.84, 3.37]	3.00E-09	60
Disorders of breast	2.48[2.08, 2.97]	9.70E-24	538
Type 2 diabetes, wide definition	2.47[2.02, 3.03]	1.80E-18	895
Polyp of the female genital tract	2.46[2.04, 2.96]	1.70E-21	488
Vaginitis/vulvovaginitis/vulvitis/abscess of vulva	2.42[1.93, 3.03]	1.60E-14	147
Obesity due to excess calories	2.37[2.02, 2.79]	3.90E-25	770
Other abnormal products of conception	2.36[1.95, 2.85]	5.40E-19	325
Other female pelvic inflammatory diseases	2.36[1.83, 3.06]	5.00E-11	92
Carpal tunnel syndrome	2.31[1.92, 2.78]	1.50E-18	808
Carcinoma in situ of cervix uteri (controls excluding all cancers)	2.3[1.70, 3.11]	6.80E-08	60
Placenta praevia	2.26[1.58, 3.23]	7.00E-06	38
Other noninflammatory disorders of vagina	2.25[1.65, 3.07]	2.90E-07	55
Other noninflammatory disorders of vulva and perineum	2.24[1.72, 2.90]	1.30E-09	91
Medical abortion	2.22[1.81, 2.73]	2.90E-14	288
Benign leiomyoma with endometriosis (controls excluding all cancers)	2.22[1.72, 2.87]	1.20E-09	90
Ectopic pregnancy	2.22[1.68, 2.95]	2.90E-08	71
Twin gestation	2.2[1.62, 2.98]	4.20E-07	59
Type 2 diabetes, definitions combined	2.19[1.83, 2.63]	2.70E-17	1301
Hypertrophy of breast in women and men	2.16[1.75, 2.65]	3.90E-13	264
Haemorrhage in early pregnancy	2.14[1.72, 2.67]	1.30E-11	169
Post-traumatic stress disorder	2.12[1.62, 2.76]	3.00E-08	90
Diabetes, insulin treatment (Kela reimbursement) (more control exclusions)	2.1[1.75, 2.51]	7.50E-16	1179
Multiple gestation	2.08[1.54, 2.81]	2.00E-06	60
Pain and other conditions associated with female genital organs and menstrual cycle	2.07[1.65, 2.59]	2.90E-10	158
Benign neoplasm of ovary	2.07[1.61, 2.67]	1.90E-08	91
Prolonged pregnancy	2.07[1.59, 2.71]	9.10E-08	91
Other specified/unspecified inflammatory spondylopathies	2.07[1.54, 2.78]	1.70E-06	61
Nerve, nerve root and plexus disorders	2.02[1.69, 2.42]	8.10E-15	1295
Dental pulpitis 1, only avohilmo	2.02[1.68, 2.43]	7.20E-14	2174
Benign mammary dysplasia	2.01[1.52, 2.66]	1.10E-06	73
HSIL lesion of the cervix uteri	1.98[1.59, 2.48]	1.80E-09	149
Otitis externa, unspecified	1.98[1.49, 2.63]	2.10E-06	78
Endometriosis	1.97[1.61, 2.40]	3.70E-11	236
Brachial plexus disorders	1.96[1.53, 2.52]	9.80E-08	105

© 2023 Tyrmi JS et al. *JAMA Cardiology.*

Endpoint	After preeclampsia diagnosis		
	HR [95 % CI]	P-value	N
**Unspecified lump in breast**	1.95[1.54, 2.46]	2.20E-08	131
**Sleep apnoea**	1.93[1.63, 2.29]	3.80E-14	910
**Rotator cuff syndrome**	1.93[1.59, 2.33]	1.40E-11	596
**Psoriatic arthropathies**	1.93[1.48, 2.52]	1.50E-06	88
**Thyroiditis**	1.93[1.45, 2.56]	5.60E-06	71
**Sleep disorders (combined)**	1.92[1.61, 2.28]	1.30E-13	1014
**All dysplastic lesions of the cervix uteri**	1.85[1.51, 2.27]	2.60E-09	214
**Spondylopathies (FG)**	1.84[1.44, 2.35]	1.10E-06	111
**Shoulder lesions**	1.83[1.53, 2.20]	6.30E-11	883
**Cholecystectomy**	1.83[1.53, 2.19]	7.00E-11	746
**Parodontitis or operation codes**	1.8[1.47, 2.22]	2.20E-08	669
**Pure hypercholesterolaemia**	1.74[1.41, 2.14]	1.50E-07	553
**Infections of the skin and subcutaneous tissue**	1.73[1.43, 2.11]	3.40E-08	365
**Hernia of abdominal wall**	1.72[1.41, 2.09]	5.60E-08	311
**Cervicobrachial syndrome**	1.72[1.41, 2.11]	1.60E-07	233
**Iron deficiency anaemia**	1.71[1.39, 2.10]	5.10E-07	332
**Other and unspecified iron deficiency**	1.71[1.38, 2.11]	6.90E-07	289
**Cervical disc disorders**	1.68[1.36, 2.06]	9.50E-07	236
**Umbilical hernia**	1.68[1.33, 2.10]	9.20E-06	144
**Nontoxic goitre/thyroid nodule**	1.67[1.34, 2.08]	5.30E-06	240
**Other diseases of liver**	1.67[1.33, 2.10]	9.50E-06	144
**Cholelithiasis**	1.65[1.37, 1.98]	7.90E-08	821
**Disorders of lipoprotein metabolism and other lipidaemias**	1.65[1.36, 2.00]	5.40E-07	760
**Erysipelas**	1.64[1.32, 2.02]	5.80E-06	314
**Chronic sinusitis**	1.63[1.35, 1.98]	5.80E-07	336
**Cardiovascular diseases (excluding rheumatic etc)**	1.62[1.34, 1.95]	4.80E-07	2538
**Other diseases of hard tissues of teeth**	1.61[1.32, 1.96]	2.70E-06	584
**TMD muscular pain linked with fibromyalgia**	1.6[1.32, 1.95]	2.70E-06	308
**Acute upper respiratory infections of multiple and unspecified sites**	1.58[1.32, 1.88]	5.30E-07	583
**Meniscus derangement**	1.58[1.30, 1.91]	3.70E-06	426
**Lower back pain or/and sciatica**	1.56[1.31, 1.86]	4.70E-07	1097
**Pain in joint**	1.56[1.31, 1.86]	4.80E-07	791
**Gonarthrosis**	1.56[1.29, 1.89]	4.60E-06	1013
**Recurrent or chronic depression**	1.54[1.29, 1.83]	2.10E-06	530
**Pain in limb**	1.53[1.28, 1.82]	2.90E-06	884
**Sciatica+with lumbago**	1.52[1.27, 1.82]	5.20E-06	533

HR = Hazard ratio, CI = Confidence interval, N = Number of overlapping individuals.

© 2023 Tyrmi JS et al. *JAMA Cardiology.*

eTable 8. Odds ratios corresponding to the top 10 % versus bottom 90 % PRSs.

	**Preeclampsia**		**Preeclampsia with severe features**		**Preeclampsia or hypertensive pregnancy**	
	**OR (95 % CI)**	**p-value**	**OR (95 % CI)**	**p-value**	**OR (95 % CI)**	**p-value**
**PE-PRS**	2.209 (1.577-3.095)	<0.001	1.890 (1.417-2.520)	<0.001	2.275 (1.617-3.200)	<0.001
**PE-HTP-PRS**	2.275 (1.617-3.200)	<0.001	1.716 (1.276-2.307)	<0.001	3.663 (2.356-5.693)	<0.001
**BP-PRS**	1.668 (1.236-2.252)	<0.001	1.386 (1.030-1.866)	0.03	1.662 (1.142-2.419)	0.008

PRS = polygenic risk score, PE = preeclampsia, PE-HTP = preeclampsia or other maternal hypertensive disorder, OR = odds ratio, CI = confidence interval.

eTable 9. Case count in top PRS deciles.

	**N top 10 % PE-PRS**	**N top 10 % PE-HTP-PRS**	**N top 10 % BP-PRS**
**Preeclampsia**	190	194	166
**Preeclampsia with severe features**	142	142	132
**Preeclampsia or hypertensive pregnancy**	208	220	199

PRS = polygenic risk score, PE = preeclampsia, PE-HTP = preeclampsia or other maternal hypertensive disorder.

eTable 10. Nagelkerkes's R^2 for models including clinical risk factors and the calculated PRSs.

	**Nagelkerke R2**		
	**Preeclampsia**	**Preeclampsia with severe features**	**Preeclampsia or hypertensive pregnancy**
**Clinical risk factors**	0.127	0.131	0.244
**Clinical risk factors and PE-PRS**	0.139	0.139	0.254
**Clinical risk factors and HTP-PRS**	0.140	0.138	0.264
**Clinical risk factors and BP-PRS**	0.127	0.133	0.247

PRS = polygenic risk score, PE = preeclampsia, PE-HTP = preeclampsia or other maternal hypertensive disorder.

© 2023 Tyrmi JS et al. *JAMA Cardiology.*

eTable 11. Sample sizes in the association analyses in the FINNPEC, FinnGen and Estonian Biobank studies and in the maternal meta-analyses of preeclampsia or indication of fetal growth restriction (PE-FGR).

Cohort	Analysis	Case N	Control N	Total N
FINNPEC	PE-FGR, maternal	1564	890	2454
EstBB	PE-FGR, maternal	2772	36 797	39 569
FinnGen	PE-FGR, maternal	6464	81 538	88 002
**Meta-analysis**	**PE-FGR, maternal**	**10 800**	**119 225**	**130 025**
FINNPEC	PE-FGR, fetal	839	852	1691
FINNPEC	PE-FGR, paternal	633	618	1251

eTable 12. Lead variants of the genome-wide significant loci from the maternal meta-analysis in the preeclampsia or indication of fetal growth restriction phenotype. Novel loci are bolded.

Phenotype	rsID	Cytoband	Chr:pos	Proposed candidate gene	EA/OA	EAF	Beta	p	+/-*
**PE-FGR**	rs16998073	4q21	4:81184341	*FGF5*	T/A	0.33	0.09	9.08E-09	+++
**PE-FGR**	**rs7470773**	**9q22**	**9:93914316**	***AUH/NFIL3***	**T/C**	**0.86**	**0.13**	**7.92E-09**	**+++**

EA = effect allele; OA = other allele; PE-FGR = preeclampsia or indication of fetal growth restriction.
*Direction of effect: FinnGen-EstBB-FINNPEC

© 2023 Tyrmi JS et al. *JAMA Cardiology.*

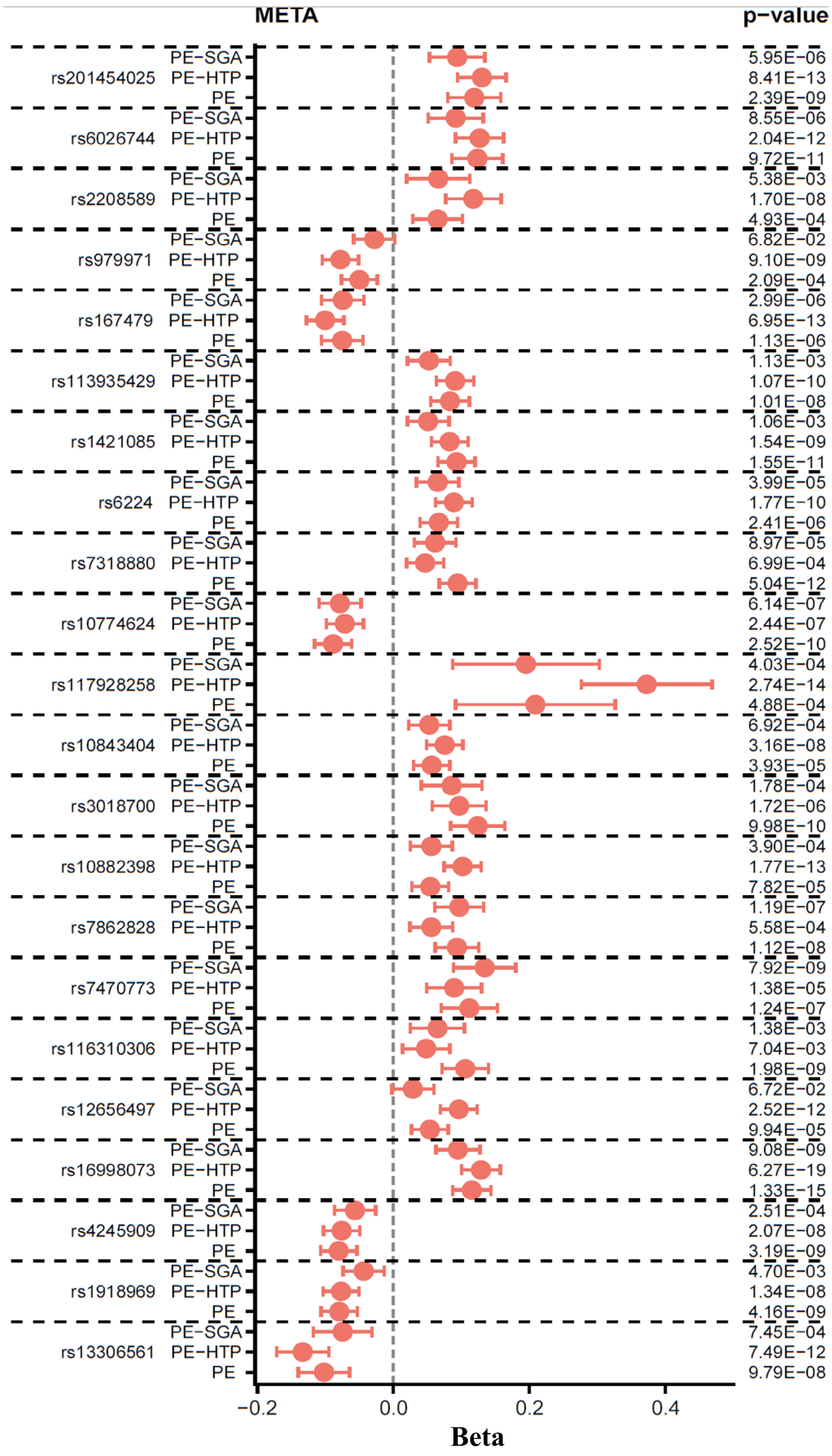

eFigure 1. Forest plot of meta-analysis results for each different phenotype. PE = preeclampsia; PE-HTP = preeclampsia or other maternal hypertensive disorder; PE-FGR = preeclampsia or fetal growth restriction.

© 2023 Tyrmi JS et al. *JAMA Cardiology.*

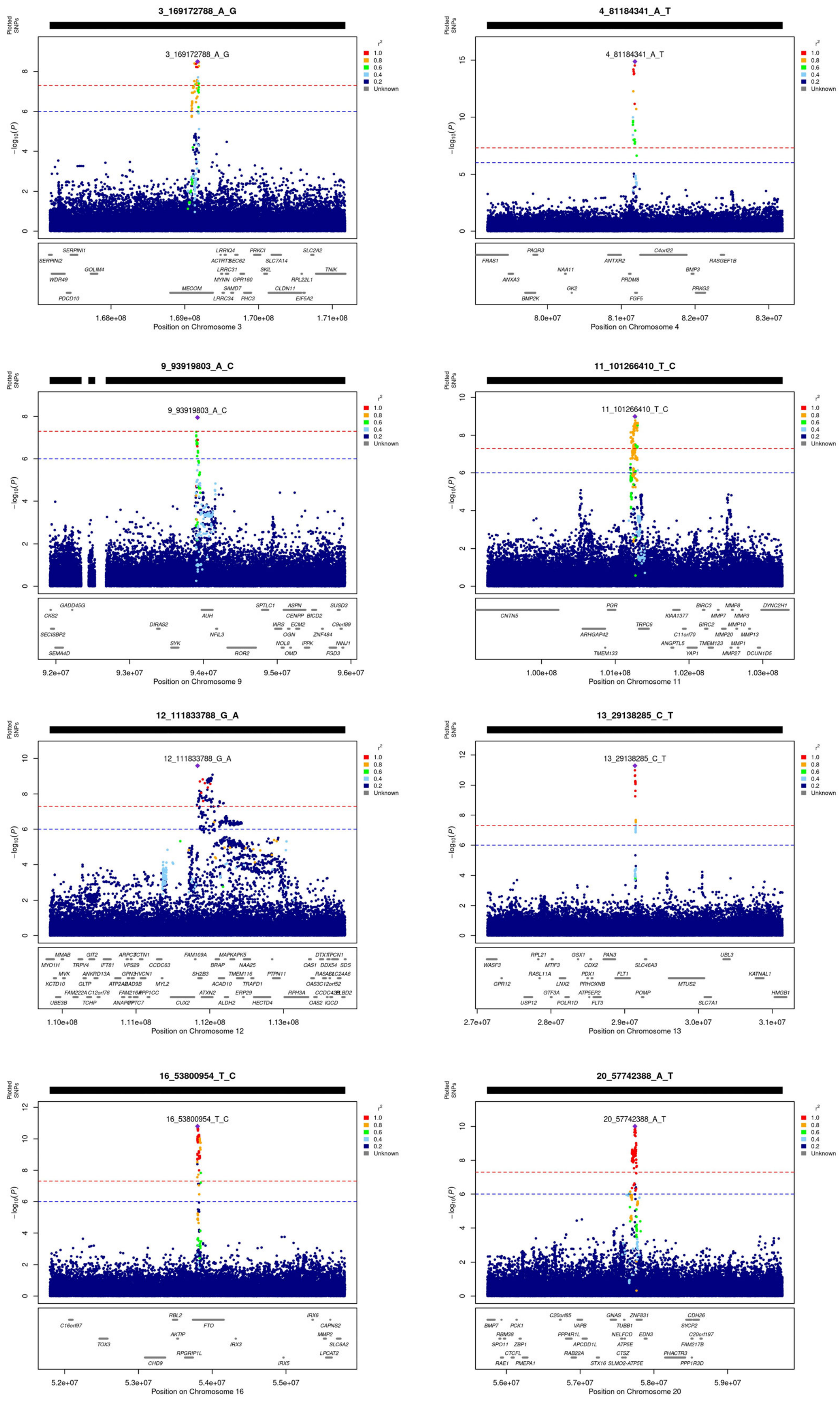

eFigure 2. Regional association plots for genome wide significant associations of preeclampsia meta-analysis. HLA-region figure could not be produced by the 'locuszooms' software due to high gene density.

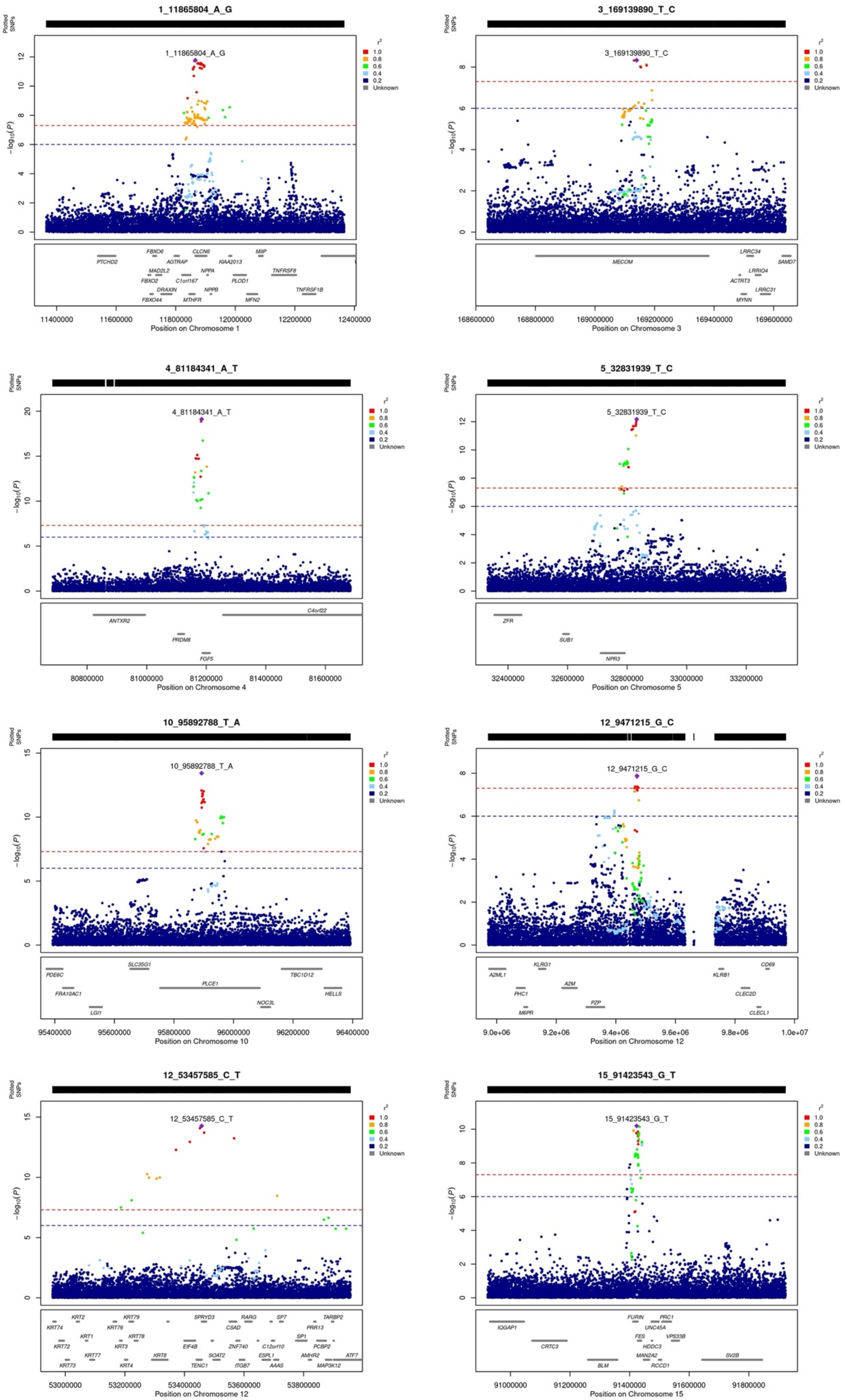

eFigure 3. Regional association plots for genome wide significant associations of preeclampsia or other maternal hypertensive disorder phenotype meta-anaylsis for chromosomes 1-15.

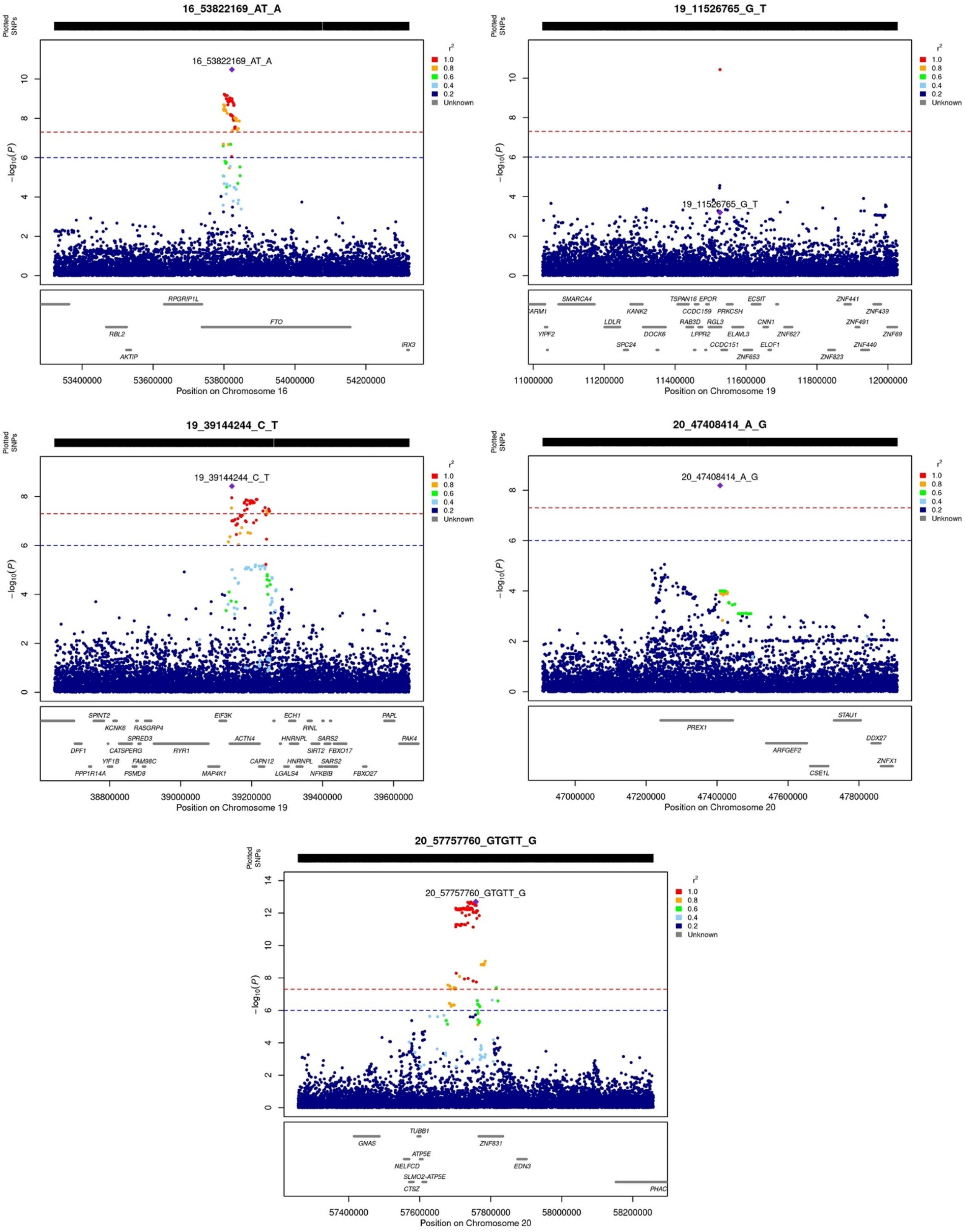

eFigure 4. Regional association plots for genome wide significant associations of preeclampsia or other maternal hypertensive phenotype for chromosomes 16-20.

© 2023 Tyrmi JS et al. *JAMA Cardiology.*

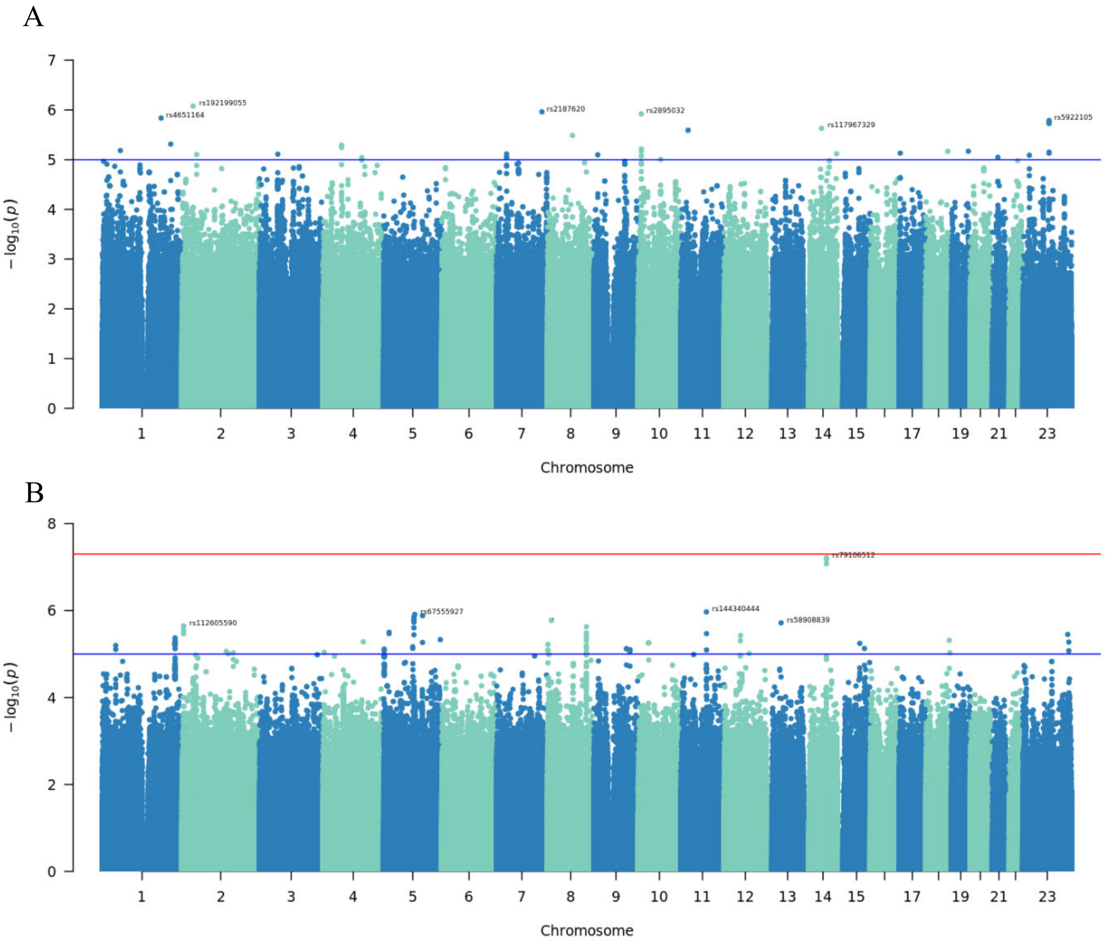

eFigure 5. Manhattan plot for preeclampsia GWAS of paternal (A) and child (B) samples.

© 2023 Tyrmi JS et al. *JAMA Cardiology.*

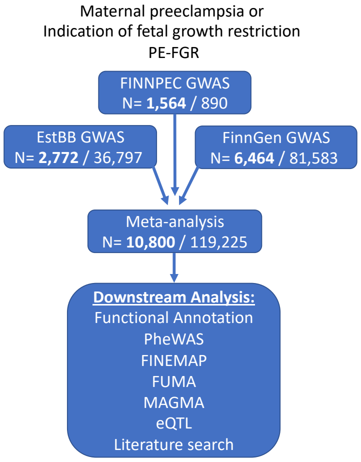

eFigure 6. Flow chart of the study design for the preeclampsia or fetal growth restriction phenotype.

© 2023 Tyrmi JS et al. *JAMA Cardiology*.

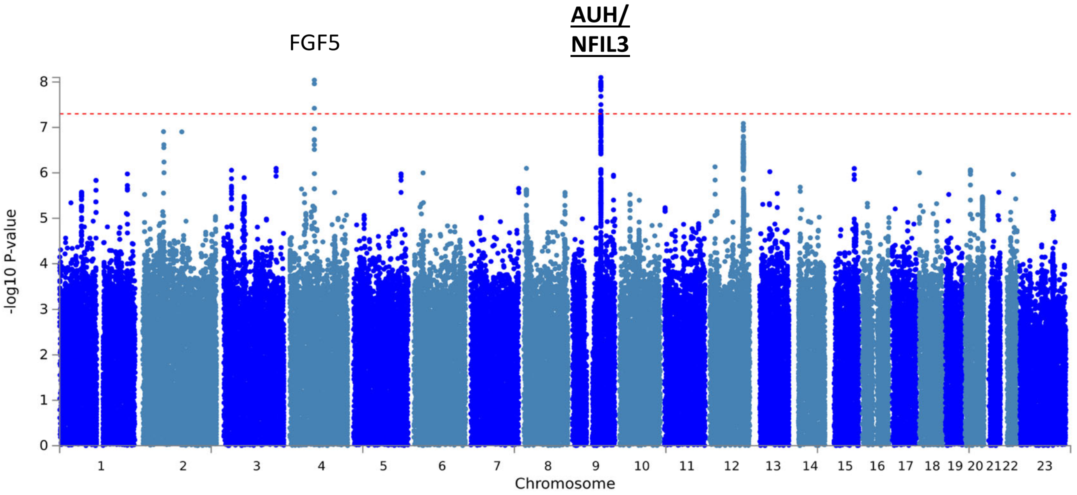

eFigure 7. Manhattan plot for the meta-analysis results of the preeclampsia or fetal growth restriction phenotype with genome-wide significant loci labeled with most likely candidate gene. Novel loci not detected in previous GWAS are bolded and underlined.

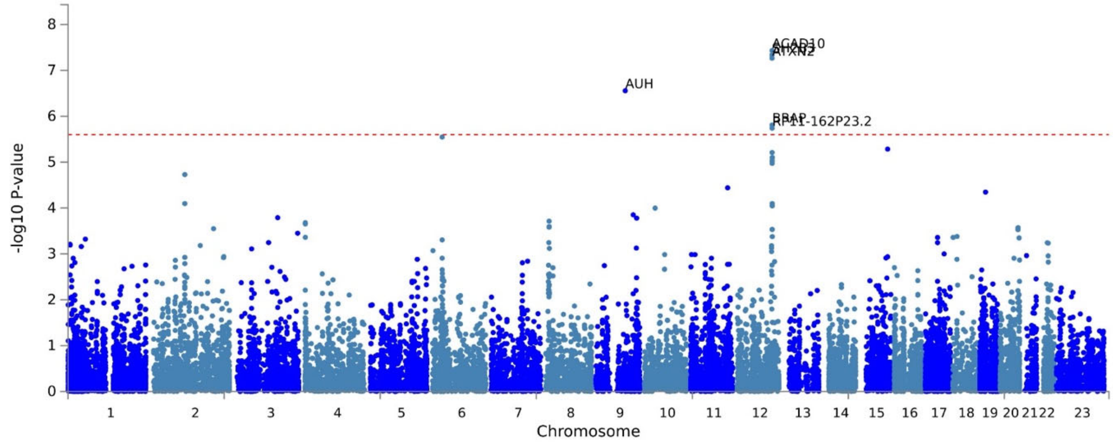

eFigure 8. MAGMA gene based test results for the meta-analysis results of the preeclampsia or fetal growth restriction phenotype.

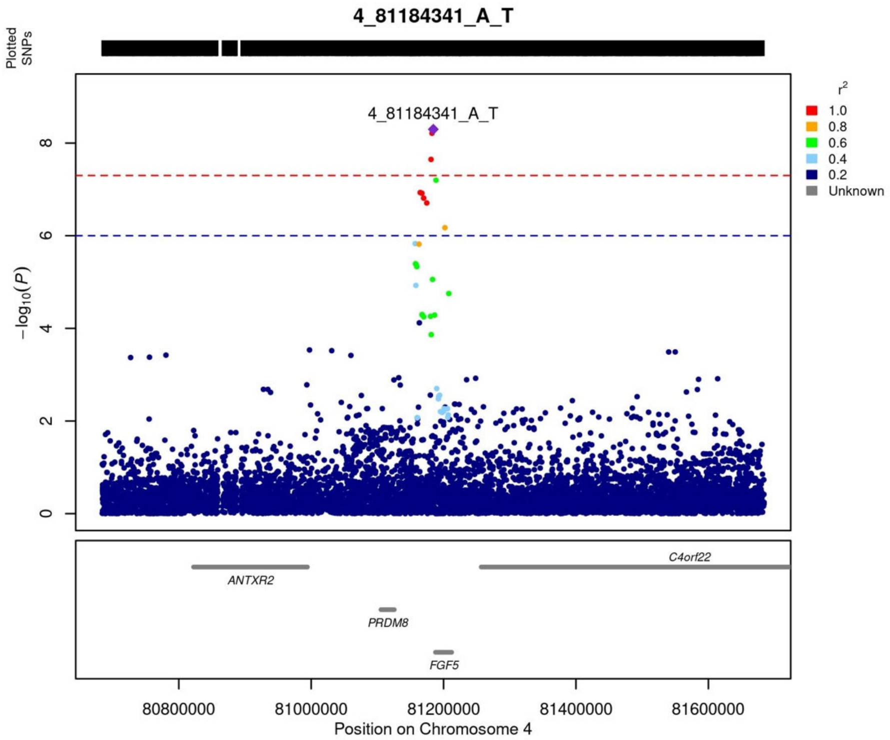

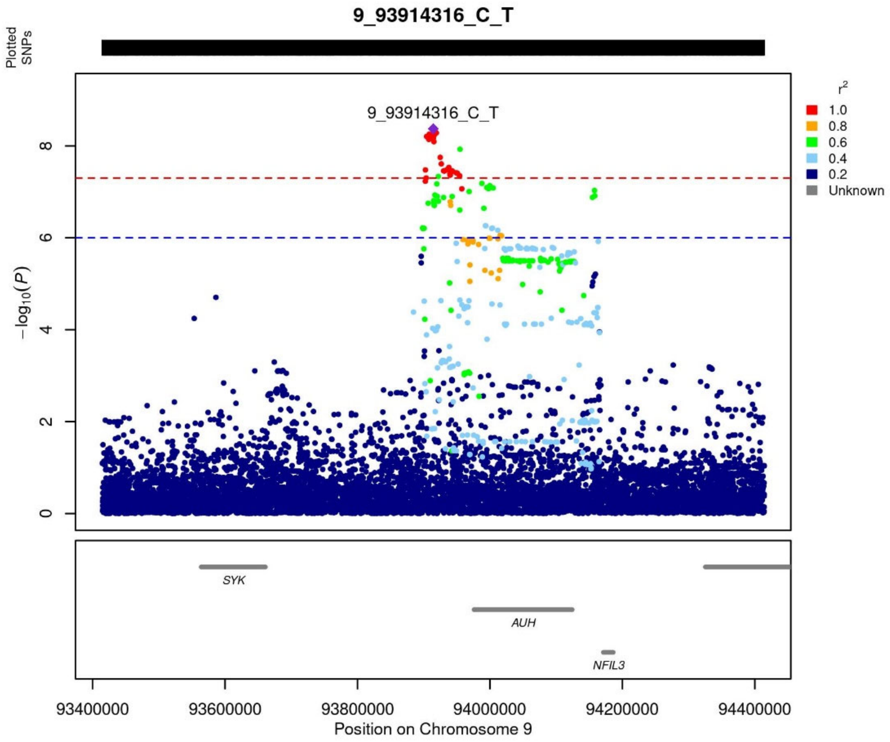

eFigure 9. Regional association plot for the preeclampsia or fetal growth restriction lead variants.

© 2023 Tyrmi JS et al. *JAMA Cardiology.*
