# Supplementary material for: Genetic Risk Factors Associated With Preeclampsia and Hypertensive Disorders of Pregnancy
Source: JAMA Cardiol. 2023 Jun 7;8(7):674–83. doi: 10.1001/jamacardio.2023.1312 (PMC10248811; doi:10.1001/jamacardio.2023.1312)
Supplement: Supplement 3. — Nonauthor collaborators [file jamacardiol-e231312-s003.pdf]

Supplemental Online Content: Nonauthor Collaborators

*Indicates required information. Only first name, last name, and suffix will appear in PubMed.

*Group Name(s): FINNPEC, FinnGen, Estonian Biobank Research Team							
***First Name and Middle Initial(s)**	***Last Name**	***Suffix (eg, Jr, III)**	Academic Degrees	Institution	Location (city, state/province, country)	Role or Contribution, eg, chair, principal investigator	Group (if more than 1 Group listed in the byline) and/or Subgroup (eg, Steering Committee)
Eeva	Ekholm		MD, PhD	Department of Obstetrics and Gynecology, Turku University Hospital,	Turku, Finland	Reseracher in charge, Turku University Hospital	FINNPEC
Reija	Hietala		MD	Department of Obstetrics and Gynecology, Helsinki University Hospital. and Helsinki University, Helsinki, Finland	Helsinki, Finland	Reseracher in charge, Helsinki University Hospital	FINNPEC
Leea	Keski-Nisula		MD, PhD	Department of Obstetrics and Gynecology, University of Eastern Finland and Kuopio University Hospital	Kuopio, Finland	Reseracher in charge, Kuopio University Hospital	FINNPEC
Kaarin	Mäkikallio		MD, PhD	Department of Obstetrics and Gynecology, Turku University Hospital	Turku, Finland	Reseracher in charge, Oulu University Hospital	FINNPEC
Jukka	Uotila		MD, PhD	Department of Obstetrics and Gynecology, Tampere University Hospital	Tampere, Finland	Reseracher in charge, Tampere University Hospital	FINNPEC
Susanna	Sainio		MD, PhD	Finnish Red Cross Blood Service	Helsinki, Finland	Reseracher in charge, Helsinki Maternity Hospital	FINNPEC
Terhi	Saisto		MD, PhD	Department of Obstetrics and Gynecology, Helsinki University Hospital. and Helsinki University	Helsinki, Finland	Reseracher in charge, Helsinki University Hospital	FINNPEC
Marja	Vääräsmäki		MD, PhD	Department of Obstetrics and Gynecology, Oulu University Hospital	Oulu, Finland	Reseracher in charge, Oulu University Hospital	FINNPEC
Tia	Aalto-Viljakainen		MD	Oncology, Helsinki University Hospital and University of Helsinki	Helsinki, Finland	clinical researcher	FINNPEC
Leena	Georgiadis		MD	Obstetrics and Gynecology, Helsinki University Hospital. and University of Eastern Finland	Kuopio, Finland	clinical researcher	FINNPEC

Supplemental Online Content: Nonauthor Collaborators

*Indicates required information. Only first name, last name, and suffix will appear in PubMed.

*First Name and Middle Initial(s)	*Last Name	*Suffix (eg, Jr, III)	Academic Degrees	Institution	Location (city, state/province, country)	Role or Contribution, eg, chair, principal investigator	Group (if more than 1 Group listed in the byline) and/or Subgroup (eg, Steering Committee)
Jenni	Heikkinen-Eloranta		MD, PhD	Obstetrics and Gynecology, Helsinki University Hospital. and University of Helsinki	Helsinki, Finland	clinical researcher	FINNPEC
Miira M.	Klemetti		MD, PhD	Obstetrics and Gynecology, Helsinki University Hospital. and University of Helsinki	Helsinki, Finland	clinical researcher	FINNPEC
Sanna	Suomalainen-König		MD	Obstetrics and Gynecology, Helsinki University Hospital. and University of Helsinki	Helsinki, Finland	clinical researcher	FINNPEC
Satu	Wedenoja		MD, PhD	Obstetrics and Gynecology, University of Helsinki	Helsinki, Finland	clinical researcher	FINNPEC
Satu	Leminen		RN	Obstetrics and Gynecology, Helsinki University Hospital and University of Helsinki	Helsinki, Finland	Research nurse	FINNPEC
Aija	Lähdesmäki		RN	Finnish Institute for Health and Welfare	Helsinki, Finland	Research nurse	FINNPEC
Susanna	Mehtälä		RN	Department of Obstetrics and Gynecology, Helsinki University Hospital and Helsinki University	Helsinki, Finland	Technician	FINNPEC
Christina	Salmén		RN	Cardiology, Helsinki University Hospital	Helsinki, Finland	Research nurse	FINNPEC
Aarno	Palotie			Institute for Molecular Medicine Finland (FIMM), HiLIFE, University of Helsinki	Helsinki, Finland	Steering Committee member	FinnGen
Mark	Daly			Institute for Molecular Medicine Finland (FIMM), HiLIFE, University of Helsinki	Helsinki, Finland	Steering Committee member	FinnGen
Bridget	Riley-Gills			Abbvie	Chicago, IL, United States	Steering Committee member	FinnGen
Howard	Jacob			Abbvie	Chicago, IL, United States	Steering Committee member	FinnGen
Dirk	Paul			Astra Zeneca	Cambridge, United Kingdom	Steering Committee member	FinnGen

*Indicates required information. Only first name, last name, and suffix will appear in PubMed.

*First Name and Middle Initial(s)	*Last Name	*Suffix (eg, Jr, III)	Academic Degrees	Institution	Location (city, state/province, country)	Role or Contribution, eg, chair, principal investigator	Group (if more than 1 Group listed in the byline) and/or Subgroup (eg, Steering Committee)
Athena	Matakidou			Astra Zeneca	Cambridge, United Kingdom	Steering Committee member	FinnGen
Adam	Platt			Astra Zeneca	Cambridge, United Kingdom	Steering Committee member	FinnGen
Heiko	Runz			Biogen	Cambridge, MA, United States	Steering Committee member	FinnGen
Sally	John			Biogen	Cambridge, MA, United States	Steering Committee member	FinnGen
George	Okafo			Boehringer Ingelheim	Ingelheim am Rhein, Germany	Steering Committee member	FinnGen
Nathan	Lawless			Boehringer Ingelheim	Ingelheim am Rhein, Germany	Steering Committee member	FinnGen
Robert	Plenge			Bristol Myers Squibb	New York, NY, United States	Steering Committee member	FinnGen
Joseph	Maranville			Bristol Myers Squibb	New York, NY, United States	Steering Committee member	FinnGen
Mark	McCarthy			Genentech	San Francisco, CA, United States	Steering Committee member	FinnGen
Julie	Hunkapiller			Genentech	San Francisco, CA, United States	Steering Committee member	FinnGen
Margaret G.	Ehm			GlaxoSmithKline	Collegeville, PA, United States	Steering Committee member	FinnGen
Kirsi	Auro			GlaxoSmithKline	Espoo, Finland	Steering Committee member	FinnGen
Simonne	Longerich			Merck	Kenilworth, NJ, United States	Steering Committee member	FinnGen
Caroline	Fox			Merck	Kenilworth, NJ, United States	Steering Committee member	FinnGen
Anders	Mälarstig			Pfizer	New York, NY, United States	Steering Committee member	FinnGen

*Indicates required information. Only first name, last name, and suffix will appear in PubMed.

***First Name and Middle Initial(s)**	***Last Name**	***Suffix (eg, Jr, III)**	Academic Degrees	Institution	Location (city, state/province, country)	Role or Contribution, eg, chair, principal investigator	Group (if more than 1 Group listed in the byline) and/or Subgroup (eg, Steering Committee)
Katherine	Klinger			Translational Sciences, Sanofi R&D	Framingham, MA, USA	Steering Committee member	FinnGen
Deepak	Raipal			Translational Sciences, Sanofi R&D	Framingham, MA, USA	Steering Committee member	FinnGen
Eric	Green			Maze Therapeutics	San Francisco, CA, United States	Steering Committee member	FinnGen
Robert	Graham			Maze Therapeutics	San Francisco, CA, United States	Steering Committee member	FinnGen
Robert	Yang			Janssen Biotech	Beerse, Belgium	Steering Committee member	FinnGen
Chris	O´Donnell			Novartis Institutes for BioMedical Research	Cambridge, MA, United States	Steering Committee member	FinnGen
Tomi P.	Mäkelä			HiLIFE, University of Helsinki	Finland, Finland	Steering Committee member	FinnGen
Jaakko	Kaprio			Institute for Molecular Medicine Finland (FIMM), HiLIFE, University of Helsinki	Helsinki, Finland	Steering Committee member	FinnGen
Petri	Virolainen			Auria Biobank, University of Turku, Hospital District of Southwest Finland	Turku, Finland	Steering Committee member	FinnGen
Antti	Hakanen			Auria Biobank, University of Turku, Hospital District of Southwest Finland	Turku, Finland	Steering Committee member	FinnGen
Terhi	Kilpi			THL Biobank, Finnish Institute for Health and Welfare (THL)	Helsinki, Finland	Steering Committee member	FinnGen
Markus	Perola			THL Biobank, Finnish Institute for Health and Welfare (THL)	Helsinki, Finland	Steering Committee member	FinnGen
Jukka	Partanen			Finnish Red Cross Blood Service, Finnish Hematology Registry and Clinical Biobank	Helsinki, Finland	Steering Committee member	FinnGen

*Indicates required information. Only first name, last name, and suffix will appear in PubMed.

***First Name and Middle Initial(s)**	***Last Name**	***Suffix (eg, Jr, III)**	Academic Degrees	Institution	Location (city, state/province, country)	Role or Contribution, eg, chair, principal investigator	Group (if more than 1 Group listed in the byline) and/or Subgroup (eg, Steering Committee)
Anne	Pitkäranta			Helsinki Biobank	Helsinki, Finland	Steering Committee member	FinnGen
Juhani	Junttila			Northern Finland Biobank Borealis, University of Oulu, Northern Ostrobothnia Hospital District	Oulu, Finland	Steering Committee member	FinnGen
Raisa	Serpi			Northern Finland Biobank Borealis, University of Oulu, Northern Ostrobothnia Hospital District	Oulu, Finland	Steering Committee member	FinnGen
Tarja	Laitinen			Finnish Clinical Biobank Tampere, University of Tampere, Pirkanmaa Hospital District	Tampere, Finland	Steering Committee member	FinnGen
Veli-Matti	Kosma			Biobank of Eastern Finland, University of Eastern Finland, Northern Savo Hospital District	Kuopio, Finland	Steering Committee member	FinnGen
Jari	Laukkanen			Central Finland Biobank, University of Jyväskylä, Central Finland Health Care District	Jyväskylä, Finland	Steering Committee member	FinnGen
Marco	Hautalahti			FINBB - Finnish biobank cooperative	Helsinki, Finland	Steering Committee member	FinnGen
Outi	Tuovila			Business Finland	Helsinki, Finland	Steering Committee member	FinnGen
Raimo	Pakkanen			Business Finland	Helsinki, Finland	Steering Committee member	FinnGen
Jeffrey	Waring			Abbvie	Chicago, IL, United States	Scientific Committee member	FinnGen
Bridget	Riley-Gillis			Abbvie	Chicago, IL, United States	Scientific Committee member	FinnGen
Fedik	Rahimov			Abbvie	Chicago, IL, United States	Scientific Committee member	FinnGen

*Indicates required information. Only first name, last name, and suffix will appear in PubMed.

***First Name and Middle Initial(s)**	***Last Name**	***Suffix (eg, Jr, III)**	Academic Degrees	Institution	Location (city, state/province, country)	Role or Contribution, eg, chair, principal investigator	Group (if more than 1 Group listed in the byline) and/or Subgroup (eg, Steering Committee)
Ioanna	Tachmazidou			Astra Zeneca	Cambridge, United Kingdom	Scientific Committee member	FinnGen
Chia-Yen	Chen			Biogen	Cambridge, MA, United States	Scientific Committee member	FinnGen
Heiko	Runz			Biogen	Cambridge, MA, United States	Scientific Committee member	FinnGen
Zhihao	Ding			Boehringer Ingelheim	Ingelheim am Rhein, Germany	Scientific Committee member	FinnGen
Marc	Jung			Boehringer Ingelheim	Ingelheim am Rhein, Germany	Scientific Committee member	FinnGen
Shameek	Biswas			Bristol Myers Squibb	New York, NY, United States	Scientific Committee member	FinnGen
Rion	Pendergrass			Genentech	San Francisco, CA, United States	Scientific Committee member	FinnGen
Julie	Hunkapiller			Genentech	San Francisco, CA, United States	Scientific Committee member	FinnGen
Margaret G.	Ehm			GlaxoSmithKline	Collegeville, PA, United States	Scientific Committee member	FinnGen
David	Pulford			GlaxoSmithKline	Stevenage, United Kingdom	Scientific Committee member	FinnGen
Neha	Raghavan			Merck	Kenilworth, NJ, United States	Scientific Committee member	FinnGen
Adriana	Huertas-Vazquez			Merck	Kenilworth, NJ, United States	Scientific Committee member	FinnGen
Jae-Hoon	Sul			Merck	Kenilworth, NJ, United States	Scientific Committee member	FinnGen
Anders	Mälarstig			Pfizer	New York, NY, United States	Scientific Committee member	FinnGen
Xinli	Hu			Pfizer	New York, NY, United States	Scientific Committee member	FinnGen

*Indicates required information. Only first name, last name, and suffix will appear in PubMed.

*First Name and Middle Initial(s)	*Last Name	*Suffix (eg, Jr, III)	Academic Degrees	Institution	Location (city, state/province, country)	Role or Contribution, eg, chair, principal investigator	Group (if more than 1 Group listed in the byline) and/or Subgroup (eg, Steering Committee)
Katherine	Klinger			Translational Sciences, Sanofi R&D	Framingham, MA, USA	Scientific Committee member	FinnGen
Robert	Graham			Maze Therapeutics	San Francisco, CA, United States	Scientific Committee member	FinnGen
Eric	Green			Maze Therapeutics	San Francisco, CA, United States	Scientific Committee member	FinnGen
Sahar	Mozaffari			Maze Therapeutics	San Francisco, CA, United States	Scientific Committee member	FinnGen
Dawn	Waterworth			Janssen Research & Development, LLC	Spring House, PA, United States	Scientific Committee member	FinnGen
Nicole	Renaud			Novartis Institutes for BioMedical Research	Cambridge, MA, United States	Scientific Committee member	FinnGen
Ma´en	Obeidat			Novartis Institutes for BioMedical Research	Cambridge, MA, United States	Scientific Committee member	FinnGen
Johanna	Schleutker			Auria Biobank, Univ. of Turku, Hospital District of Southwest Finland	Turku, Finland	Scientific Committee member	FinnGen
Markus	Perola			THL Biobank, Finnish Institute for Health and Welfare (THL)	Helsinki, Finland	Scientific Committee member	FinnGen
Mikko	Arvas			Finnish Red Cross Blood Service, Finnish Hematology Registry and Clinical Biobank	Helsinki, Finland	Scientific Committee member	FinnGen
Olli	Carpén			Helsinki Biobank	Helsinki, Finland	Scientific Committee member	FinnGen
Reetta	Hinttala			Northern Finland Biobank Borealis, University of Oulu, Northern Ostrobothnia Hospital District	Oulu, Finland	Scientific Committee member	FinnGen
Arto	Mannermaa			Biobank of Eastern Finland, University of Eastern Finland, Northern Savo Hospital District	Kuopio, Finland	Scientific Committee member	FinnGen

*Indicates required information. Only first name, last name, and suffix will appear in PubMed.

***First Name and Middle Initial(s)**	***Last Name**	***Suffix (eg, Jr, III)**	Academic Degrees	Institution	Location (city, state/province, country)	Role or Contribution, eg, chair, principal investigator	Group (if more than 1 Group listed in the byline) and/or Subgroup (eg, Steering Committee)
Katriina	Aalto-Setälä			Faculty of Medicine and Health Technology, Tampere University	Tampere, Finland	Scientific Committee member	FinnGen
Mika	Kähönen			Finnish Clinical Biobank Tampere, University of Tampere, Pirkanmaa Hospital District	Tampere, Finland	Scientific Committee member	FinnGen
Jari	Laukkanen			Central Finland Biobank, University of Jyväskylä, Central Finland Health Care District	Jyväskylä, Finland	Scientific Committee member	FinnGen
Johanna	Mäkelä			FINBB - Finnish biobank cooperative	Helsinki, Finland	Scientific Committee member	FinnGen
Reetta	Kälviäinen			Northern Savo Hospital District	Kuopio, Finland	Clinical Groups member	FinnGen
Valtteri	Julkunen			Northern Savo Hospital District	Kuopio, Finland	Clinical Groups member	FinnGen
Hilkka	Soininen			Northern Savo Hospital District	Kuopio, Finland	Clinical Groups member	FinnGen
Anne	Remes			Northern Ostrobothnia Hospital District	Oulu, Finland	Clinical Groups member	FinnGen
Mikko	Hiltunen			University of Eastern Finland	Kuopio, Finland	Clinical Groups member	FinnGen
Jukka	Peltola			Pirkanmaa Hospital District	Tampere, Finland	Clinical Groups member	FinnGen
Minna	Raivio			Hospital District of Helsinki and Uusimaa	Helsinki, Finland	Clinical Groups member	FinnGen
Pentti	Tienari			Hospital District of Helsinki and Uusimaa	Helsinki, Finland	Clinical Groups member	FinnGen
Juha	Rinne			Hospital District of Southwest Finland	Turku, Finland	Clinical Groups member	FinnGen
Roosa	Kallionpää			Hospital District of Southwest Finland	Turku, Finland	Clinical Groups member	FinnGen

*Indicates required information. Only first name, last name, and suffix will appear in PubMed.

*First Name and Middle Initial(s)	*Last Name	*Suffix (eg, Jr, III)	Academic Degrees	Institution	Location (city, state/province, country)	Role or Contribution, eg, chair, principal investigator	Group (if more than 1 Group listed in the byline) and/or Subgroup (eg, Steering Committee)
Juulia	Partanen			Institute for Molecular Medicine Finland, HiLIFE	Helsinki, Finland	Clinical Groups member	FinnGen
Ali	Abbasi			Abbvie	Chicago, IL, United States	Clinical Groups member	FinnGen
Adam	Ziemann			Abbvie	Chicago, IL, United States	Clinical Groups member	FinnGen
Nizar	Smaoui			Abbvie	Chicago, IL, United States	Clinical Groups member	FinnGen
Anne	Lehtonen			Abbvie	Chicago, IL, United States	Clinical Groups member	FinnGen
Susan	Eaton			Biogen	Cambridge, MA, United States	Clinical Groups member	FinnGen
Heiko	Runz			Biogen	Cambridge, MA, United States	Clinical Groups member	FinnGen
Sanni	Lahdenperä			Biogen	Cambridge, MA, United States	Clinical Groups member	FinnGen
Shameek	Biswas			Bristol Myers Squibb	New York, NY, United States	Clinical Groups member	FinnGen
Julie	Hunkapiller			Genentech	San Francisco, CA, United States	Clinical Groups member	FinnGen
Natalie	Bowers			Genentech	San Francisco, CA, United States	Clinical Groups member	FinnGen
Edmond	Teng			Genentech	San Francisco, CA, United States	Clinical Groups member	FinnGen
Rion	Pendergrass			Genentech	San Francisco, CA, United States	Clinical Groups member	FinnGen
Fanli	Xu			GlaxoSmithKline	Brentford, United Kingdom	Clinical Groups member	FinnGen
David	Pulford			GlaxoSmithKline	Stevenage, United Kingdom	Clinical Groups member	FinnGen

*Indicates required information. Only first name, last name, and suffix will appear in PubMed.

*First Name and Middle Initial(s)	*Last Name	*Suffix (eg, Jr, III)	Academic Degrees	Institution	Location (city, state/province, country)	Role or Contribution, eg, chair, principal investigator	Group (if more than 1 Group listed in the byline) and/or Subgroup (eg, Steering Committee)
Kirsi	Auro			GlaxoSmithKline	Espoo, Finland	Clinical Groups member	FinnGen
Laura	Addis			GlaxoSmithKline	Brentford, United Kingdom	Clinical Groups member	FinnGen
John	Eicher			GlaxoSmithKline	Brentford, United Kingdom	Clinical Groups member	FinnGen
Qingqin S	Li			Janssen Research & Development, LLC	Titusville, NJ 08560, United States	Clinical Groups member	FinnGen
Karen	He			Janssen Research & Development, LLC	Spring House, PA, United States	Clinical Groups member	FinnGen
Ekaterina	Khramtsova			Janssen Research & Development, LLC	Spring House, PA, United States	Clinical Groups member	FinnGen
Neha	Raghavan			Merck	Kenilworth, NJ, United States	Clinical Groups member	FinnGen
Martti	Färkkilä			Hospital District of Helsinki and Uusimaa	Helsinki, Finland	Clinical Groups member	FinnGen
Jukka	Koskela			Hospital District of Helsinki and Uusimaa	Helsinki, Finland	Clinical Groups member	FinnGen
Sampsa	Pikkarainen			Hospital District of Helsinki and Uusimaa	Helsinki, Finland	Clinical Groups member	FinnGen
Airi	Jussila			Pirkanmaa Hospital District	Tampere, Finland	Clinical Groups member	FinnGen
Katri	Kaukinen			Pirkanmaa Hospital District	Tampere, Finland	Clinical Groups member	FinnGen
Timo	Blomster			Northern Ostrobothnia Hospital District	Oulu, Finland	Clinical Groups member	FinnGen
Mikko	Kiviniemi			Northern Savo Hospital District	Kuopio, Finland	Clinical Groups member	FinnGen
Markku	Voutilainen			Hospital District of Southwest Finland	Turku, Finland	Clinical Groups member	FinnGen

*Indicates required information. Only first name, last name, and suffix will appear in PubMed.

*First Name and Middle Initial(s)	*Last Name	*Suffix (eg, Jr, III)	Academic Degrees	Institution	Location (city, state/province, country)	Role or Contribution, eg, chair, principal investigator	Group (if more than 1 Group listed in the byline) and/or Subgroup (eg, Steering Committee)
Mark	Daly			Institute for Molecular Medicine, Finland (FIMM), HiLIFE, University of Helsinki	Helsinki, Finland	Clinical Groups member	FinnGen
Ali	Abbasi			Abbvie	Chicago, IL, United States	Clinical Groups member	FinnGen
Jeffrey	Waring			Abbvie	Chicago, IL, United States	Clinical Groups member	FinnGen
Nizar	Smaoui			Abbvie	Chicago, IL, United States	Clinical Groups member	FinnGen
Fedik	Rahimov			Abbvie	Chicago, IL, United States	Clinical Groups member	FinnGen
Anne	Lehtonen			Abbvie	Chicago, IL, United States	Clinical Groups member	FinnGen
Tim	Lu			Genentech	San Francisco, CA, United States	Clinical Groups member	FinnGen
Natalie	Bowers			Genentech	San Francisco, CA, United States	Clinical Groups member	FinnGen
Rion	Pendergrass			Genentech	San Francisco, CA, United States	Clinical Groups member	FinnGen
Linda	McCarthy			GlaxoSmithKline	Brentford, United Kingdom	Clinical Groups member	FinnGen
Amy	Hart			Janssen Research & Development, LLC	Spring House, PA, United States	Clinical Groups member	FinnGen
Meijian	Guan			Janssen Research & Development, LLC	Spring House, PA, United States	Clinical Groups member	FinnGen
Jason	Miller			Merck	Kenilworth, NJ, United States	Clinical Groups member	FinnGen
Kirsi	Kalpala			Pfizer	New York, NY, United States	Clinical Groups member	FinnGen
Melissa	Miller			Pfizer	New York, NY, United States	Clinical Groups member	FinnGen

Supplemental Online Content: Nonauthor Collaborators

*Indicates required information. Only first name, last name, and suffix will appear in PubMed.

*First Name and Middle Initial(s)	*Last Name	*Suffix (eg, Jr, III)	Academic Degrees	Institution	Location (city, state/province, country)	Role or Contribution, eg, chair, principal investigator	Group (if more than 1 Group listed in the byline) and/or Subgroup (eg, Steering Committee)
Xinli	Hu			Pfizer	New York, NY, United States	Clinical Groups member	FinnGen
Kari	Eklund			Hospital District of Helsinki and Uusimaa	Helsinki, Finland	Clinical Groups member	FinnGen
Antti	Palomäki			Hospital District of Southwest Finland	Turku, Finland	Clinical Groups member	FinnGen
Pia	Isomäki			Pirkanmaa Hospital District	Tampere, Finland	Clinical Groups member	FinnGen
Laura	Pirilä			Hospital District of Southwest Finland	Turku, Finland	Clinical Groups member	FinnGen
Oili	Kaipiainen-Seppänen			Northern Savo Hospital District	Kuopio, Finland	Clinical Groups member	FinnGen
Johanna	Huhtakangas			Northern Ostrobothnia Hospital District	Oulu, Finland	Clinical Groups member	FinnGen
Nina	Mars			Institute for Molecular Medicine Finland (FIMM), HiLIFE, University of Helsinki	Helsinki, Finland	Clinical Groups member	FinnGen
Ali	Abbasi			Abbvie	Chicago, IL, United States	Clinical Groups member	FinnGen
Jeffrey	Waring			Abbvie	Chicago, IL, United States	Clinical Groups member	FinnGen
Fedik	Rahimov			Abbvie	Chicago, IL, United States	Clinical Groups member	FinnGen
Apinya	Lertratanakul			Abbvie	Chicago, IL, United States	Clinical Groups member	FinnGen
Nizar	Smaoui			Abbvie	Chicago, IL, United States	Clinical Groups member	FinnGen
Anne	Lehtonen			Abbvie	Chicago, IL, United States	Clinical Groups member	FinnGen
David	Close			Astra Zeneca	Cambridge, United Kingdom	Clinical Groups member	FinnGen

*Indicates required information. Only first name, last name, and suffix will appear in PubMed.

*First Name and Middle Initial(s)	*Last Name	*Suffix (eg, Jr, III)	Academic Degrees	Institution	Location (city, state/province, country)	Role or Contribution, eg, chair, principal investigator	Group (if more than 1 Group listed in the byline) and/or Subgroup (eg, Steering Committee)
Marla	Hochfeld			Bristol Myers Squibb	New York, NY, United States	Clinical Groups member	FinnGen
Natalie	Bowers			Genentech	San Francisco, CA, United States	Clinical Groups member	FinnGen
Rion	Pendergrass			Genentech	San Francisco, CA, United States	Clinical Groups member	FinnGen
Jorge Esparza	Gordillo			GlaxoSmithKline	Brentford, United Kingdom	Clinical Groups member	FinnGen
Kirsi	Auro			GlaxoSmithKline	Espoo, Finland	Clinical Groups member	FinnGen
Dawn	Waterworth			Janssen Research & Development, LLC	Spring House, PA, United States	Clinical Groups member	FinnGen
Fabiana	Farias			Merck	Kenilworth, NJ, United States	Clinical Groups member	FinnGen
Kirsi	Kalpala			Pfizer	New York, NY, United States	Clinical Groups member	FinnGen
Nan	Bing			Pfizer	New York, NY, United States	Clinical Groups member	FinnGen
Xinli	Hu			Pfizer	New York, NY, United States	Clinical Groups member	FinnGen
Tarja	Laitinen			Pirkanmaa Hospital District	Tampere, Finland	Clinical Groups member	FinnGen
Margit	Pelkonen			Northern Savo Hospital District	Kuopio, Finland	Clinical Groups member	FinnGen
Paula	Kauppi			Hospital District of Helsinki and Uusimaa	Helsinki, Finland	Clinical Groups member	FinnGen
Hannu	Kankaanranta			Tampere University	Tampere, Finland	Clinical Groups member	FinnGen
Terttu	Harju			Northern Ostrobothnia Hospital District	Oulu, Finland	Clinical Groups member	FinnGen

*Indicates required information. Only first name, last name, and suffix will appear in PubMed.

*First Name and Middle Initial(s)	*Last Name	*Suffix (eg, Jr, III)	Academic Degrees	Institution	Location (city, state/province, country)	Role or Contribution, eg, chair, principal investigator	Group (if more than 1 Group listed in the byline) and/or Subgroup (eg, Steering Committee)
Riitta	Lahesmaa			Hospital District of Southwest Finland	Turku, Finland	Clinical Groups member	FinnGen
Nizar	Smaoui			Abbvie	Chicago, IL, United States	Clinical Groups member	FinnGen
Alex	Mackay			Astra Zeneca	Cambridge, United Kingdom	Clinical Groups member	FinnGen
Glenda	Lassi			Astra Zeneca	Cambridge, United Kingdom	Clinical Groups member	FinnGen
Susan	Eaton			Biogen	Cambridge, MA, United States	Clinical Groups member	FinnGen
Hubert	Chen			Genentech	San Francisco, CA, United States	Clinical Groups member	FinnGen
Rion	Pendergrass			Genentech	San Francisco, CA, United States	Clinical Groups member	FinnGen
Natalie	Bowers			Genentech	San Francisco, CA, United States	Clinical Groups member	FinnGen
Joanna	Betts			GlaxoSmithKline	Brentford, United Kingdom	Clinical Groups member	FinnGen
Kirsi	Auro			GlaxoSmithKline	Espoo, Finland	Clinical Groups member	FinnGen
Rajashree	Mishra			GlaxoSmithKline	Brentford, United Kingdom	Clinical Groups member	FinnGen
Majd	Mouded			Novartis	Basel, Switzerland	Clinical Groups member	FinnGen
Debby	Ngo			Novartis	Basel, Switzerland	Clinical Groups member	FinnGen
Teemu	Niiranen			Finnish Institute for Health and Welfare (THL)	Helsinki, Finland	Clinical Groups member	FinnGen
Felix	Vaura			Finnish Institute for Health and Welfare (THL)	Helsinki, Finland	Clinical Groups member	FinnGen

*Indicates required information. Only first name, last name, and suffix will appear in PubMed.

*First Name and Middle Initial(s)	*Last Name	*Suffix (eg, Jr, III)	Academic Degrees	Institution	Location (city, state/province, country)	Role or Contribution, eg, chair, principal investigator	Group (if more than 1 Group listed in the byline) and/or Subgroup (eg, Steering Committee)
Veikko	Salomaa			Finnish Institute for Health and Welfare (THL)	Helsinki, Finland	Clinical Groups member	FinnGen
Kaj	Metsärinne			Hospital District of Southwest Finland	Turku, Finland	Clinical Groups member	FinnGen
Jenni	Aittokallio			Hospital District of Southwest Finland	Turku, Finland	Clinical Groups member	FinnGen
Mika	Kähönen			Pirkanmaa Hospital District	Tampere, Finland	Clinical Groups member	FinnGen
Jussi	Hernesniemi			Pirkanmaa Hospital District	Tampere, Finland	Clinical Groups member	FinnGen
Daniel	Gordin			Hospital District of Helsinki and Uusimaa	Helsinki, Finland	Clinical Groups member	FinnGen
Juha	Sinisalo			Hospital District of Helsinki and Uusimaa	Helsinki, Finland	Clinical Groups member	FinnGen
Marja-Riitta	Taskinen			Hospital District of Helsinki and Uusimaa	Helsinki, Finland	Clinical Groups member	FinnGen
Tiinamaija	Tuomi			Hospital District of Helsinki and Uusimaa	Helsinki, Finland	Clinical Groups member	FinnGen
Timo	Hiltunen			Hospital District of Helsinki and Uusimaa	Helsinki, Finland	Clinical Groups member	FinnGen
Jari	Laukkanen			Central Finland Health Care District	Jyväskylä, Finland	Clinical Groups member	FinnGen
Amanda	Elliott			Institute for Molecular Medicine Finland (FIMM), HiLIFE, University of Helsinki	Helsinki, Finland	Clinical Groups member	FinnGen
Mary Pat	Reeve			Institute for Molecular Medicine Finland (FIMM), HiLIFE, University of Helsinki	Helsinki, Finland	Clinical Groups member	FinnGen
Benjamin	Challis			Astra Zeneca	Cambridge, United Kingdom	Clinical Groups member	FinnGen

*Indicates required information. Only first name, last name, and suffix will appear in PubMed.

*First Name and Middle Initial(s)	*Last Name	*Suffix (eg, Jr, III)	Academic Degrees	Institution	Location (city, state/province, country)	Role or Contribution, eg, chair, principal investigator	Group (if more than 1 Group listed in the byline) and/or Subgroup (eg, Steering Committee)
Dirk	Paul			Astra Zeneca	Cambridge, United Kingdom	Clinical Groups member	FinnGen
Julie	Hunkapiller			Genentech	San Francisco, CA, United States	Clinical Groups member	FinnGen
Natalie	Bowers			Genentech	San Francisco, CA, United States	Clinical Groups member	FinnGen
Rion	Pendergrass			Genentech	San Francisco, CA, United States	Clinical Groups member	FinnGen
Audrey	Chu			GlaxoSmithKline	Brentford, United Kingdom	Clinical Groups member	FinnGen
Kirsi	Auro			GlaxoSmithKline	Espoo, Finland	Clinical Groups member	FinnGen
Dermot	Reilly			Janssen Research & Development, LLC	Boston, MA, United States	Clinical Groups member	FinnGen
Mike	Mendelson			Novartis	Boston, MA, United States	Clinical Groups member	FinnGen
Jaakko	Parkkinen			Pfizer	New York, NY, United States	Clinical Groups member	FinnGen
Melissa	Miller			Pfizer	New York, NY, United States	Clinical Groups member	FinnGen
Tuomo	Meretoja			Hospital District of Helsinki and Uusimaa	Helsinki, Finland	Clinical Groups member	FinnGen
Heikki	Joensuu			Hospital District of Helsinki and Uusimaa	Helsinki, Finland	Clinical Groups member	FinnGen
Olli	Carpén			Hospital District of Helsinki and Uusimaa	Helsinki, Finland	Clinical Groups member	FinnGen
Johanna	Mattson			Hospital District of Helsinki and Uusimaa	Helsinki, Finland	Clinical Groups member	FinnGen
Eveliina	Salminen			Hospital District of Helsinki and Uusimaa	Helsinki, Finland	Clinical Groups member	FinnGen

*Indicates required information. Only first name, last name, and suffix will appear in PubMed.

*First Name and Middle Initial(s)	*Last Name	*Suffix (eg, Jr, III)	Academic Degrees	Institution	Location (city, state/province, country)	Role or Contribution, eg, chair, principal investigator	Group (if more than 1 Group listed in the byline) and/or Subgroup (eg, Steering Committee)
Annika	Auranen			Pirkanmaa Hospital District	Tampere, Finland	Clinical Groups member	FinnGen
Peeter	Karihtala			Northern Ostrobothnia Hospital District	Oulu, Finland	Clinical Groups member	FinnGen
Päivi	Auvinen			Northern Savo Hospital District	Kuopio, Finland	Clinical Groups member	FinnGen
Klaus	Elenius			Hospital District of Southwest Finland	Turku, Finland	Clinical Groups member	FinnGen
Johanna	Schleutker			Hospital District of Southwest Finland	Turku, Finland	Clinical Groups member	FinnGen
Esa	Pitkänen			Institute for Molecular Medicine Finland (FIMM), HiLIFE, University of Helsinki	Helsinki, Finland	Clinical Groups member	FinnGen
Nina	Mars			Institute for Molecular Medicine Finland (FIMM), HiLIFE, University of Helsinki	Helsinki, Finland	Clinical Groups member	FinnGen
Mark	Daly			Institute for Molecular Medicine Finland (FIMM), HiLIFE, University of Helsinki	Helsinki, Finland	Clinical Groups member	FinnGen
Relja	Popovic			Abbvie	Chicago, IL, United States	Clinical Groups member	FinnGen
Jeffrey	Waring			Abbvie	Chicago, IL, United States	Clinical Groups member	FinnGen
Bridget	Riley-Gillis			Abbvie	Chicago, IL, United States	Clinical Groups member	FinnGen
Anne	Lehtonen			Abbvie	Chicago, IL, United States	Clinical Groups member	FinnGen
Jennifer	Schutzman			Genentech	San Francisco, CA, United States	Clinical Groups member	FinnGen
Julie	Hunkapiller			Genentech	San Francisco, CA, United States	Clinical Groups member	FinnGen

*Indicates required information. Only first name, last name, and suffix will appear in PubMed.

*First Name and Middle Initial(s)	*Last Name	*Suffix (eg, Jr, III)	Academic Degrees	Institution	Location (city, state/province, country)	Role or Contribution, eg, chair, principal investigator	Group (if more than 1 Group listed in the byline) and/or Subgroup (eg, Steering Committee)
Natalie	Bowers			Genentech	San Francisco, CA, United States	Clinical Groups member	FinnGen
Rion	Pendergrass			Genentech	San Francisco, CA, United States	Clinical Groups member	FinnGen
Diptee	Kulkarni			GlaxoSmithKline	Brentford, United Kingdom	Clinical Groups member	FinnGen
Kirsi	Auro			GlaxoSmithKline	Espoo, Finland	Clinical Groups member	FinnGen
Alessandro	Porello			Janssen Research & Development, LLC	Spring House, PA, United States	Clinical Groups member	FinnGen
Andrey	Loboda			Merck	Kenilworth, NJ, United States	Clinical Groups member	FinnGen
Heli	Lehtonen			Pfizer	New York, NY, United States	Clinical Groups member	FinnGen
Stefan	McDonough			Pfizer	New York, NY, United States	Clinical Groups member	FinnGen
Sauli	Vuoti			Janssen-Cilag Oy	Espoo, Finland	Clinical Groups member	FinnGen
Kai	Kaarniranta			Northern Savo Hospital District	Kuopio, Finland	Clinical Groups member	FinnGen
Joni A	Turunen			Helsinki University Hospital and University of Helsinki	Helsinki, Finland	Clinical Groups member	FinnGen
Terhi	Ollila			Hospital District of Helsinki and Uusimaa	Helsinki, Finland	Clinical Groups member	FinnGen
Hannu	Uusitalo			Pirkanmaa Hospital District	Tampere, Finland	Clinical Groups member	FinnGen
Esa	Pitkänen			Institute for Molecular Medicine Finland (FIMM), HiLIFE, University of Helsinki	Helsinki, Finland	Clinical Groups member	FinnGen
Mengzhen	Liu			Abbvie	Chicago, IL, United States	Clinical Groups member	FinnGen

*Indicates required information. Only first name, last name, and suffix will appear in PubMed.

*First Name and Middle Initial(s)	*Last Name	*Suffix (eg, Jr, III)	Academic Degrees	Institution	Location (city, state/province, country)	Role or Contribution, eg, chair, principal investigator	Group (if more than 1 Group listed in the byline) and/or Subgroup (eg, Steering Committee)
Heiko	Runz			Biogen	Cambridge, MA, United States	Clinical Groups member	FinnGen
Stephanie	Loomis			Biogen	Cambridge, MA, United States	Clinical Groups member	FinnGen
Erich	Strauss			Genentech	San Francisco, CA, United States	Clinical Groups member	FinnGen
Natalie	Bowers			Genentech	San Francisco, CA, United States	Clinical Groups member	FinnGen
Hao	Chen			Genentech	San Francisco, CA, United States	Clinical Groups member	FinnGen
Rion	Pendergrass			Genentech	San Francisco, CA, United States	Clinical Groups member	FinnGen
Kaisa	Tasanen			Northern Ostrobothnia Hospital District	Oulu, Finland	Clinical Groups member	FinnGen
Laura	Huilaja			Northern Ostrobothnia Hospital District	Oulu, Finland	Clinical Groups member	FinnGen
Katariina	Hannula-Jouppi			Hospital District of Helsinki and Uusimaa	Helsinki, Finland	Clinical Groups member	FinnGen
Teea	Salmi			Pirkanmaa Hospital District	Tampere, Finland	Clinical Groups member	FinnGen
Sirkku	Peltonen			Hospital District of Southwest Finland	Turku, Finland	Clinical Groups member	FinnGen
Leena	Koulu			Hospital District of Southwest Finland	Turku, Finland	Clinical Groups member	FinnGen
Nizar	Smaoui			Abbvie	Chicago, IL, United States	Clinical Groups member	FinnGen
Fedik	Rahimov			Abbvie	Chicago, IL, United States	Clinical Groups member	FinnGen
Anne	Lehtonen			Abbvie	Chicago, IL, United States	Clinical Groups member	FinnGen

Supplemental Online Content: Nonauthor Collaborators

*Indicates required information. Only first name, last name, and suffix will appear in PubMed.

*First Name and Middle Initial(s)	*Last Name	*Suffix (eg, Jr, III)	Academic Degrees	Institution	Location (city, state/province, country)	Role or Contribution, eg, chair, principal investigator	Group (if more than 1 Group listed in the byline) and/or Subgroup (eg, Steering Committee)
David	Choy			Genentech	San Francisco, CA, United States	Clinical Groups member	FinnGen
Rion	Pendergrass			Genentech	San Francisco, CA, United States	Clinical Groups member	FinnGen
Dawn	Waterworth			Janssen Research & Development, LLC	Spring House, PA, United States	Clinical Groups member	FinnGen
Kirsi	Kalpala			Pfizer	New York, NY, United States	Clinical Groups member	FinnGen
Ying	Wu			Pfizer	New York, NY, United States	Clinical Groups member	FinnGen
Pirkko	Pussinen			Hospital District of Helsinki and Uusimaa	Helsinki, Finland	Clinical Groups member	FinnGen
Aino	Salminen			Hospital District of Helsinki and Uusimaa	Helsinki, Finland	Clinical Groups member	FinnGen
Tuula	Salo			Hospital District of Helsinki and Uusimaa	Helsinki, Finland	Clinical Groups member	FinnGen
David	Rice			Hospital District of Helsinki and Uusimaa	Helsinki, Finland	Clinical Groups member	FinnGen
Pekka	Nieminen			Hospital District of Helsinki and Uusimaa	Helsinki, Finland	Clinical Groups member	FinnGen
Ulla	Palotie			Hospital District of Helsinki and Uusimaa	Helsinki, Finland	Clinical Groups member	FinnGen
Maria	Siponen			Northern Savo Hospital District	Kuopio, Finland	Clinical Groups member	FinnGen
Liisa	Suominen			Northern Savo Hospital District	Kuopio, Finland	Clinical Groups member	FinnGen
Päivi	Mäntylä			Northern Savo Hospital District	Kuopio, Finland	Clinical Groups member	FinnGen
Ulvi	Gursoy			Hospital District of Southwest Finland	Turku, Finland	Clinical Groups member	FinnGen

*Indicates required information. Only first name, last name, and suffix will appear in PubMed.

*First Name and Middle Initial(s)	*Last Name	*Suffix (eg, Jr, III)	Academic Degrees	Institution	Location (city, state/province, country)	Role or Contribution, eg, chair, principal investigator	Group (if more than 1 Group listed in the byline) and/or Subgroup (eg, Steering Committee)
Vuokko	Anttonen			Northern Ostrobothnia Hospital District	Oulu, Finland	Clinical Groups member	FinnGen
Kirsi	Sipilä			Research Unit of Oral Health Sciences Faculty of Medicine, University of Oulu	Oulu, Finland	Clinical Groups member	FinnGen
Rion	Pendergrass			Genentech	San Francisco, CA, United States	Clinical Groups member	FinnGen
Venla	Kurra			Pirkanmaa Hospital District	Tampere, Finland	Clinical Groups member	FinnGen
Laura	Kotaniemi-Talonen			Pirkanmaa Hospital District	Tampere, Finland	Clinical Groups member	FinnGen
Oskari	Heikinheimo			Hospital District of Helsinki and Uusimaa	Helsinki, Finland	Clinical Groups member	FinnGen
Ilkka	Kalliala			Hospital District of Helsinki and Uusimaa	Helsinki, Finland	Clinical Groups member	FinnGen
Lauri	Aaltonen			Hospital District of Helsinki and Uusimaa	Helsinki, Finland	Clinical Groups member	FinnGen
Varpu	Jokimaa			Hospital District of Southwest Finland	Turku, Finland	Clinical Groups member	FinnGen
Johannes	Kettunen			Northern Ostrobothnia Hospital District	Oulu, Finland	Clinical Groups member	FinnGen
Marja	Vääräsmäki			Northern Ostrobothnia Hospital District	Oulu, Finland	Clinical Groups member	FinnGen
Outi	Uimari			Northern Ostrobothnia Hospital District	Oulu, Finland	Clinical Groups member	FinnGen
Laure	Morin-Papunen			Northern Ostrobothnia Hospital District	Oulu, Finland	Clinical Groups member	FinnGen
Maarit	Niinimäki			Northern Ostrobothnia Hospital District	Oulu, Finland	Clinical Groups member	FinnGen
Terhi	Piltonen			Northern Ostrobothnia Hospital District	Oulu, Finland	Clinical Groups member	FinnGen

*Indicates required information. Only first name, last name, and suffix will appear in PubMed.

***First Name and Middle Initial(s)**	***Last Name**	***Suffix (eg, Jr, III)**	Academic Degrees	Institution	Location (city, state/province, country)	Role or Contribution, eg, chair, principal investigator	Group (if more than 1 Group listed in the byline) and/or Subgroup (eg, Steering Committee)
Elisabeth	Widen			Institute for Molecular Medicine Finland (FIMM), HiLIFE, University of Helsinki	Helsinki, Finland	Clinical Groups member	FinnGen
Taru	Tukiainen			Institute for Molecular Medicine Finland (FIMM), HiLIFE, University of Helsinki	Helsinki, Finland	Clinical Groups member	FinnGen
Mary Pat	Reeve			Institute for Molecular Medicine Finland (FIMM), HiLIFE, University of Helsinki	Helsinki, Finland	Clinical Groups member	FinnGen
Mark	Daly			Institute for Molecular Medicine Finland (FIMM), HiLIFE, University of Helsinki	Helsinki, Finland	Clinical Groups member	FinnGen
Niko	Välimäki			University of Helsinki	Helsinki, Finland	Clinical Groups member	FinnGen
Eija	Laakkonen			University of Jyväskylä	Jyväskylä, Finland	Clinical Groups member	FinnGen
Heidi	Silven			University of Oulu	Oulu, Finland	Clinical Groups member	FinnGen
Eeva	Sliz			University of Oulu	Oulu, Finland	Clinical Groups member	FinnGen
Riikka	Arffman			University of Oulu	Oulu, Finland	Clinical Groups member	FinnGen
Susanna	Savukoski			University of Oulu	Oulu, Finland	Clinical Groups member	FinnGen
Natalia	Pujol			Estonian biobank	Tartu, Estonia	Clinical Groups member	FinnGen
Mengzhen	Liu			Abbvie	Chicago, IL, United States	Clinical Groups member	FinnGen
Bridget	Riley-Gillis			Abbvie	Chicago, IL, United States	Clinical Groups member	FinnGen

*Indicates required information. Only first name, last name, and suffix will appear in PubMed.

*First Name and Middle Initial(s)	*Last Name	*Suffix (eg, Jr, III)	Academic Degrees	Institution	Location (city, state/province, country)	Role or Contribution, eg, chair, principal investigator	Group (if more than 1 Group listed in the byline) and/or Subgroup (eg, Steering Committee)
Rion	Pendergrass			Genentech	San Francisco, CA, United States	Clinical Groups member	FinnGen
Janet	Kumar			GlaxoSmithKline	Collegeville, PA, United States	Clinical Groups member	FinnGen
Kirsi	Auro			GlaxoSmithKline	Espoo, Finland	Clinical Groups member	FinnGen
Iiris	Hovatta			University of Helsinki	Helsinki, Finland	Clinical Groups member	FinnGen
Chia-Yen	Chen			Biogen	Cambridge, MA, United States	Clinical Groups member	FinnGen
Erkki	Isometsä			Hospital District of Helsinki and Uusimaa	Helsinki, Finland	Clinical Groups member	FinnGen
Kumar	Veerapen			Broad Institute	Cambridge, MA, United States	Clinical Groups member	FinnGen
Hanna	Ollila			Institute for Molecular Medicine Finland (FIMM), HiLIFE, University of Helsinki	Helsinki, Finland	Clinical Groups member	FinnGen
Jaana	Suvisaari			Finnish Institute for Health and Welfare (THL)	Helsinki, Finland	Clinical Groups member	FinnGen
Thomas D.	Als			Aarhus University	Aarhus, Denmark	Clinical Groups member	FinnGen
Antti	Mäkitie			Department of Otorhinolaryngology - Head and Neck Surgery, University of Helsinki and Helsinki University Hospital	Helsinki, Finland	Clinical Groups member	FinnGen
Argyro	Bizaki-Vallaskangas			Pirkanmaa Hospital District	Tampere, Finland	Clinical Groups member	FinnGen
Sanna	Toppila-Salmi			University of Helsinki	Helsinki, Finland	Clinical Groups member	FinnGen

*Indicates required information. Only first name, last name, and suffix will appear in PubMed.

*First Name and Middle Initial(s)	*Last Name	*Suffix (eg, Jr, III)	Academic Degrees	Institution	Location (city, state/province, country)	Role or Contribution, eg, chair, principal investigator	Group (if more than 1 Group listed in the byline) and/or Subgroup (eg, Steering Committee)
Tytti	Willberg			Hospital District of Southwest Finland	Turku, Finland	Clinical Groups member	FinnGen
Elmo	Saarentaus			Institute for Molecular Medicine Finland (FIMM), HiLIFE, University of Helsinki	Helsinki, Finland	Clinical Groups member	FinnGen
Antti	Aarnisalo			Hospital District of Helsinki and Uusimaa	Helsinki, Finland	Clinical Groups member	FinnGen
Eveliina	Salminen			Hospital District of Helsinki and Uusimaa	Helsinki, Finland	Clinical Groups member	FinnGen
Elisa	Rahikkala			Northern Ostrobothnia Hospital District	Oulu, Finland	Clinical Groups member	FinnGen
Johannes	Kettunen			Northern Ostrobothnia Hospital District	Oulu, Finland	Clinical Groups member	FinnGen
Kristiina	Aittomäki			Department of Medical Genetics, Helsinki University Central Hospital	Helsinki, Finland	Clinical Groups member	FinnGen
Fredrik	Åberg			Transplantation and Liver Surgery Clinic, Helsinki University Hospital, Helsinki University	Helsinki, Finland	Clinical Groups member	FinnGen
Mitja	Kurki			Institute for Molecular Medicine Finland (FIMM), HiLIFE, University of Helsinki	Helsinki, Finland	FinnGen Analysis working group member	FinnGen
Samuli	Ripatti			Institute for Molecular Medicine Finland (FIMM), HiLIFE, University of Helsinki	Helsinki, Finland	FinnGen Analysis working group member	FinnGen
Mark	Daly			Institute for Molecular Medicine, Finland (FIMM), HiLIFE, University of Helsinki	Helsinki, Finland	FinnGen Analysis working group member	FinnGen
Juha	Karjalainen			Institute for Molecular Medicine Finland (FIMM), HiLIFE, University of Helsinki	Helsinki, Finland	FinnGen Analysis working group member	FinnGen

*Indicates required information. Only first name, last name, and suffix will appear in PubMed.

*First Name and Middle Initial(s)	*Last Name	*Suffix (eg, Jr, III)	Academic Degrees	Institution	Location (city, state/province, country)	Role or Contribution, eg, chair, principal investigator	Group (if more than 1 Group listed in the byline) and/or Subgroup (eg, Steering Committee)
Aki	Havulinna			Institute for Molecular Medicine Finland (FIMM), HiLIFE, University of Helsinki	Helsinki, Finland	FinnGen Analysis working group member	FinnGen
Juha	Mehtonen			Institute for Molecular Medicine Finland (FIMM), HiLIFE, University of Helsinki	Helsinki, Finland	FinnGen Analysis working group member	FinnGen
Priit	Palta			Institute for Molecular Medicine Finland (FIMM), HiLIFE, University of Helsinki	Helsinki, Finland	FinnGen Analysis working group member	FinnGen
Shabbeer	Hassan			Institute for Molecular Medicine Finland (FIMM), HiLIFE, University of Helsinki	Helsinki, Finland	FinnGen Analysis working group member	FinnGen
Pietro Della Briotta	Parolo			Institute for Molecular Medicine Finland (FIMM), HiLIFE, University of Helsinki	Helsinki, Finland	FinnGen Analysis working group member	FinnGen
Wei	Zhou			Broad Institute	Cambridge, MA, United States	FinnGen Analysis working group member	FinnGen
Mutaamba	Maasha			Broad Institute	Cambridge, MA, United States	FinnGen Analysis working group member	FinnGen
Kumar	Veerapen			Broad Institute	Cambridge, MA, United States	FinnGen Analysis working group member	FinnGen
Shabbeer	Hassan			Institute for Molecular Medicine Finland (FIMM), HiLIFE, University of Helsinki	Helsinki, Finland	FinnGen Analysis working group member	FinnGen
Susanna	Lemmelä			Institute for Molecular Medicine Finland (FIMM), HiLIFE, University of Helsinki	Helsinki, Finland	FinnGen Analysis working group member	FinnGen

*Indicates required information. Only first name, last name, and suffix will appear in PubMed.

***First Name and Middle Initial(s)**	***Last Name**	***Suffix (eg, Jr, III)**	Academic Degrees	Institution	Location (city, state/province, country)	Role or Contribution, eg, chair, principal investigator	Group (if more than 1 Group listed in the byline) and/or Subgroup (eg, Steering Committee)
Manuel	Rivas			University of Stanford	Stanford, CA, United States	FinnGen Analysis working group member	FinnGen
Mari E.	Niemi			Institute for Molecular Medicine Finland (FIMM), HiLIFE, University of Helsinki	Helsinki, Finland	FinnGen Analysis working group member	FinnGen
Aarno	Palotie			Institute for Molecular Medicine Finland (FIMM), HiLIFE, University of Helsinki	Helsinki, Finland	FinnGen Analysis working group member	FinnGen
Aoxing	Liu			Institute for Molecular Medicine Finland (FIMM), HiLIFE, University of Helsinki	Helsinki, Finland	FinnGen Analysis working group member	FinnGen
Arto	Lehisto			Institute for Molecular Medicine Finland (FIMM), HiLIFE, University of Helsinki	Helsinki, Finland	FinnGen Analysis working group member	FinnGen
Andrea	Ganna			Institute for Molecular Medicine Finland (FIMM), HiLIFE, University of Helsinki	Helsinki, Finland	FinnGen Analysis working group member	FinnGen
Vincent	Llorens			Institute for Molecular Medicine Finland (FIMM), HiLIFE, University of Helsinki	Helsinki, Finland	FinnGen Analysis working group member	FinnGen
Taru	Tukiainen			Institute for Molecular Medicine Finland (FIMM), HiLIFE, University of Helsinki	Helsinki, Finland	FinnGen Analysis working group member	FinnGen
Mary Pat	Reeve			Institute for Molecular Medicine Finland (FIMM), HiLIFE, University of Helsinki	Helsinki, Finland	FinnGen Analysis working group member	FinnGen
Henrike	Heyne			Institute for Molecular Medicine Finland (FIMM), HiLIFE, University of Helsinki	Helsinki, Finland	FinnGen Analysis working group member	FinnGen

*Indicates required information. Only first name, last name, and suffix will appear in PubMed.

*First Name and Middle Initial(s)	*Last Name	*Suffix (eg, Jr, III)	Academic Degrees	Institution	Location (city, state/province, country)	Role or Contribution, eg, chair, principal investigator	Group (if more than 1 Group listed in the byline) and/or Subgroup (eg, Steering Committee)
Nina	Mars			Institute for Molecular Medicine Finland (FIMM), HiLIFE, University of Helsinki	Helsinki, Finland	FinnGen Analysis working group member	FinnGen
Joel	Rämö			Institute for Molecular Medicine Finland (FIMM), HiLIFE, University of Helsinki	Helsinki, Finland	FinnGen Analysis working group member	FinnGen
Elmo	Saarentaus			Institute for Molecular Medicine Finland (FIMM), HiLIFE, University of Helsinki	Helsinki, Finland	FinnGen Analysis working group member	FinnGen
Hanna	Ollila			Institute for Molecular Medicine Finland (FIMM), HiLIFE, University of Helsinki	Helsinki, Finland	FinnGen Analysis working group member	FinnGen
Rodos	Rodosthenous			Institute for Molecular Medicine Finland (FIMM), HiLIFE, University of Helsinki	Helsinki, Finland	FinnGen Analysis working group member	FinnGen
Satu	Strausz			Institute for Molecular Medicine Finland (FIMM), HiLIFE, University of Helsinki	Helsinki, Finland	FinnGen Analysis working group member	FinnGen
Tuula	Palotie			University of Helsinki and Hospital District of Helsinki and Uusimaa	Helsinki, Finland	FinnGen Analysis working group member	FinnGen
Kimmo	Palin			University of Helsinki	Helsinki, Finland	FinnGen Analysis working group member	FinnGen
Javier	Garcia-Tabuenca			University of Tampere	Tampere, Finland	FinnGen Analysis working group member	FinnGen
Harri	Siirtola			University of Tampere	Tampere, Finland	FinnGen Analysis working group member	FinnGen

*Indicates required information. Only first name, last name, and suffix will appear in PubMed.

***First Name and Middle Initial(s)**	***Last Name**	***Suffix (eg, Jr, III)**	Academic Degrees	Institution	Location (city, state/province, country)	Role or Contribution, eg, chair, principal investigator	Group (if more than 1 Group listed in the byline) and/or Subgroup (eg, Steering Committee)
Tuomo	Kiiskinen			Institute for Molecular Medicine Finland (FIMM), HiLIFE, University of Helsinki	Helsinki, Finland	FinnGen Analysis working group member	FinnGen
Jiwoo	Lee			Institute for Molecular Medicine Finland (FIMM), HiLIFE, University of Helsinki	Helsinki, Finland	FinnGen Analysis working group member	FinnGen
Kristin	Tsuo			Institute for Molecular Medicine Finland (FIMM), HiLIFE, University of Helsinki	Helsinki, Finland	FinnGen Analysis working group member	FinnGen
Amanda	Elliott			Institute for Molecular Medicine Finland (FIMM), HiLIFE, University of Helsinki	Helsinki, Finland	FinnGen Analysis working group member	FinnGen
Kati	Kristiansson			THL Biobank, Finnish Institute for Health and Welfare (THL)	Helsinki, Finland	FinnGen Analysis working group member	FinnGen
Mikko	Arvas			Finnish Red Cross Blood Service, Finnish Hematology Registry and Clinical Biobank	Helsinki, Finland	FinnGen Analysis working group member	FinnGen
Kati	Hyvärinen			Finnish Red Cross Blood Service	Helsinki, Finland	FinnGen Analysis working group member	FinnGen
Jarmo	Ritari			Finnish Red Cross Blood Service	Helsinki, Finland	FinnGen Analysis working group member	FinnGen
Olli	Carpén			Helsinki Biobank	Helsinki, Finland	FinnGen Analysis working group member	FinnGen
Johannes	Kettunen			Northern Finland Biobank Borealis, University of Oulu, Northern Ostrobothnia Hospital District	Oulu, Finland	FinnGen Analysis working group member	FinnGen

*Indicates required information. Only first name, last name, and suffix will appear in PubMed.

***First Name and Middle Initial(s)**	***Last Name**	***Suffix (eg, Jr, III)**	Academic Degrees	Institution	Location (city, state/province, country)	Role or Contribution, eg, chair, principal investigator	Group (if more than 1 Group listed in the byline) and/or Subgroup (eg, Steering Committee)
Katri	Pylkäs			University of Oulu	Oulu, Finland	FinnGen Analysis working group member	FinnGen
Eeva	Sliz			University of Oulu	Oulu, Finland	FinnGen Analysis working group member	FinnGen
Minna	Karjalainen			University of Oulu	Oulu, Finland	FinnGen Analysis working group member	FinnGen
Tuomo	Mantere			Northern Finland Biobank Borealis, University of Oulu, Northern Ostrobothnia Hospital District	Oulu, Finland	FinnGen Analysis working group member	FinnGen
Eeva	Kangasniemi			Finnish Clinical Biobank Tampere, University of Tampere, Pirkanmaa Hospital District	Tampere, Finland	FinnGen Analysis working group member	FinnGen
Sami	Heikkinen			University of Eastern Finland	Kuopio, Finland	FinnGen Analysis working group member	FinnGen
Arto	Mannermaa			Biobank of Eastern Finland, University of Eastern Finland, Northern Savo Hospital District	Kuopio, Finland	FinnGen Analysis working group member	FinnGen
Eija	Laakkonen			University of Jyväskylä	Jyväskylä, Finland	FinnGen Analysis working group member	FinnGen
Nina	Pitkänen			Auria Biobank, University of Turku, Hospital District of Southwest Finland	Turku, Finland	FinnGen Analysis working group member	FinnGen
Samuel	Lessard			Translational Sciences, Sanofi R&D	Framingham, MA, USA	FinnGen Analysis working group member	FinnGen

*Indicates required information. Only first name, last name, and suffix will appear in PubMed.

***First Name and Middle Initial(s)**	***Last Name**	***Suffix (eg, Jr, III)**	Academic Degrees	Institution	Location (city, state/province, country)	Role or Contribution, eg, chair, principal investigator	Group (if more than 1 Group listed in the byline) and/or Subgroup (eg, Steering Committee)
Clément	Chatelain			Translational Sciences, Sanofi R&D	Framingham, MA, USA	FinnGen Analysis working group member	FinnGen
Perttu	Terho			Auria Biobank, University of Turku, Hospital District of Southwest Finland	Turku, Finland	Biobank directors member	FinnGen
Sirpa	Soini			THL Biobank, Finnish Institute for Health and Welfare (THL)	Helsinki, Finland	Biobank directors member	FinnGen
Jukka	Partanen			Finnish Red Cross Blood Service, Finnish Hematology Registry and Clinical Biobank	Helsinki, Finland	Biobank directors member	FinnGen
Eero	Punkka			Helsinki Biobank	Helsinki, Finland	Biobank directors member	FinnGen
Raisa	Serpi			Northern Finland Biobank Borealis, University of Oulu, Northern Ostrobothnia Hospital District	Oulu, Finland	Biobank directors member	FinnGen
Sanna	Siltanen			Finnish Clinical Biobank Tampere, University of Tampere, Pirkanmaa Hospital District	Tampere, Finland	Biobank directors member	FinnGen
Veli-Matti	Kosma			Biobank of Eastern Finland, University of Eastern Finland, Northern Savo Hospital District	Kuopio, Finland	Biobank directors member	FinnGen
Teijo	Kuopio			Central Finland Biobank, University of Jyväskylä, Central Finland Health Care District	Jyväskylä, Finland	Biobank directors member	FinnGen
Anu	Jalanko			Institute for Molecular Medicine Finland (FIMM), HiLIFE, University of Helsinki	Helsinki, Finland	FinnGen Teams member	FinnGen

*Indicates required information. Only first name, last name, and suffix will appear in PubMed.

*First Name and Middle Initial(s)	*Last Name	*Suffix (eg, Jr, III)	Academic Degrees	Institution	Location (city, state/province, country)	Role or Contribution, eg, chair, principal investigator	Group (if more than 1 Group listed in the byline) and/or Subgroup (eg, Steering Committee)
Huei-Yi	Shen			Institute for Molecular Medicine Finland (FIMM), HiLIFE, University of Helsinki	Helsinki, Finland	FinnGen Teams member	FinnGen
Risto	Kajanne			Institute for Molecular Medicine Finland (FIMM), HiLIFE, University of Helsinki	Helsinki, Finland	FinnGen Teams member	FinnGen
Mervi	Aavikko			Institute for Molecular Medicine Finland (FIMM), HiLIFE, University of Helsinki	Helsinki, Finland	FinnGen Teams member	FinnGen
Mitja	Kurki			Institute for Molecular Medicine Finland (FIMM), HiLIFE, University of Helsinki	Helsinki, Finland	FinnGen Teams member	FinnGen
Juha	Karjalainen			Institute for Molecular Medicine Finland (FIMM), HiLIFE, University of Helsinki	Helsinki, Finland	FinnGen Teams member	FinnGen
Pietro Della Briotta	Parolo			Institute for Molecular Medicine Finland (FIMM), HiLIFE, University of Helsinki	Helsinki, Finland	FinnGen Teams member	FinnGen
Arto	Lehisto			Institute for Molecular Medicine Finland (FIMM), HiLIFE, University of Helsinki	Helsinki, Finland	FinnGen Teams member	FinnGen
Juha	Mehtonen			Institute for Molecular Medicine Finland (FIMM), HiLIFE, University of Helsinki	Helsinki, Finland	FinnGen Teams member	FinnGen
Wei	Zhou			Broad Institute	Cambridge, MA, United States	FinnGen Teams member	FinnGen
Masahiro	Kanai			Broad Institute	Cambridge, MA, United States	FinnGen Teams member	FinnGen
Mutaamba	Maasha			Broad Institute	Cambridge, MA, United States	FinnGen Teams member	FinnGen

*Indicates required information. Only first name, last name, and suffix will appear in PubMed.

*First Name and Middle Initial(s)	*Last Name	*Suffix (eg, Jr, III)	Academic Degrees	Institution	Location (city, state/province, country)	Role or Contribution, eg, chair, principal investigator	Group (if more than 1 Group listed in the byline) and/or Subgroup (eg, Steering Committee)
Kumar	Veerapen			Broad Institute	Cambridge, MA, United States	FinnGen Teams member	FinnGen
Hannele	Laivuori			Institute for Molecular Medicine Finland (FIMM), HiLIFE, University of Helsinki	Helsinki, Finland	FinnGen Teams member	FinnGen
Aki	Havulinna			Institute for Molecular Medicine Finland (FIMM), HiLIFE, University of Helsinki	Helsinki, Finland	FinnGen Teams member	FinnGen
Susanna	Lemmelä			Institute for Molecular Medicine Finland (FIMM), HiLIFE, University of Helsinki	Helsinki, Finland	FinnGen Teams member	FinnGen
Tuomo	Kiiskinen			Institute for Molecular Medicine Finland (FIMM), HiLIFE, University of Helsinki	Helsinki, Finland	FinnGen Teams member	FinnGen
L. Elisa	Lahtela			Institute for Molecular Medicine Finland (FIMM), HiLIFE, University of Helsinki	Helsinki, Finland	FinnGen Teams member	FinnGen
Mari	Kaunisto			Institute for Molecular Medicine Finland (FIMM), HiLIFE, University of Helsinki	Helsinki, Finland	FinnGen Teams member	FinnGen
Elina	Kilpeläinen			Institute for Molecular Medicine Finland (FIMM), HiLIFE, University of Helsinki	Helsinki, Finland	FinnGen Teams member	FinnGen
Timo P.	Sipilä			Institute for Molecular Medicine Finland (FIMM), HiLIFE, University of Helsinki	Helsinki, Finland	FinnGen Teams member	FinnGen
Oluwaseun Alexander	Dada			Institute for Molecular Medicine Finland (FIMM), HiLIFE, University of Helsinki	Helsinki, Finland	FinnGen Teams member	FinnGen

*Indicates required information. Only first name, last name, and suffix will appear in PubMed.

***First Name and Middle Initial(s)**	***Last Name**	***Suffix (eg, Jr, III)**	Academic Degrees	Institution	Location (city, state/province, country)	Role or Contribution, eg, chair, principal investigator	Group (if more than 1 Group listed in the byline) and/or Subgroup (eg, Steering Committee)
Awaisa	Ghazal			Institute for Molecular Medicine Finland (FIMM), HiLIFE, University of Helsinki	Helsinki, Finland	FinnGen Teams member	FinnGen
Anastasia	Kytölä			Institute for Molecular Medicine Finland (FIMM), HiLIFE, University of Helsinki	Helsinki, Finland	FinnGen Teams member	FinnGen
Rigbe	Weldatsadik			Institute for Molecular Medicine Finland (FIMM), HiLIFE, University of Helsinki	Helsinki, Finland	FinnGen Teams member	FinnGen
Kati	Donner			Institute for Molecular Medicine Finland (FIMM), HiLIFE, University of Helsinki	Helsinki, Finland	FinnGen Teams member	FinnGen
Timo P.	Sipilä			Institute for Molecular Medicine Finland (FIMM), HiLIFE, University of Helsinki	Helsinki, Finland	FinnGen Teams member	FinnGen
Anu	Loukola			Helsinki Biobank	Helsinki, Finland	FinnGen Teams member	FinnGen
Päivi	Laiho			THL Biobank, Finnish Institute for Health and Welfare (THL)	Helsinki, Finland	FinnGen Teams member	FinnGen
Tuuli	Sistonen			THL Biobank, Finnish Institute for Health and Welfare (THL)	Helsinki, Finland	FinnGen Teams member	FinnGen
Essi	Kaiharju			THL Biobank, Finnish Institute for Health and Welfare (THL)	Helsinki, Finland	FinnGen Teams member	FinnGen
Markku	Laukkanen			THL Biobank, Finnish Institute for Health and Welfare (THL)	Helsinki, Finland	FinnGen Teams member	FinnGen
Elina	Järvensivu			THL Biobank, Finnish Institute for Health and Welfare (THL)	Helsinki, Finland	FinnGen Teams member	FinnGen
Sini	Lähteenmäki			THL Biobank, Finnish Institute for Health and Welfare (THL)	Helsinki, Finland	FinnGen Teams member	FinnGen
Lotta	Männikkö			THL Biobank, Finnish Institute for Health and Welfare (THL)	Helsinki, Finland	FinnGen Teams member	FinnGen

*Indicates required information. Only first name, last name, and suffix will appear in PubMed.

*First Name and Middle Initial(s)	*Last Name	*Suffix (eg, Jr, III)	Academic Degrees	Institution	Location (city, state/province, country)	Role or Contribution, eg, chair, principal investigator	Group (if more than 1 Group listed in the byline) and/or Subgroup (eg, Steering Committee)
Regis	Wong			THL Biobank, Finnish Institute for Health and Welfare (THL)	Helsinki, Finland	FinnGen Teams member	FinnGen
Auli	Toivola			THL Biobank, Finnish Institute for Health and Welfare (THL)	Helsinki, Finland	FinnGen Teams member	FinnGen
Minna	Brunfeldt			THL Biobank, Finnish Institute for Health and Welfare (THL)	Helsinki, Finland	FinnGen Teams member	FinnGen
Hannele	Mattsson			THL Biobank, Finnish Institute for Health and Welfare (THL)	Helsinki, Finland	FinnGen Teams member	FinnGen
Kati	Kristiansson			THL Biobank, Finnish Institute for Health and Welfare (THL)	Helsinki, Finland	FinnGen Teams member	FinnGen
Susanna	Lemmelä			Institute for Molecular Medicine Finland (FIMM), HiLIFE, University of Helsinki	Helsinki, Finland	FinnGen Teams member	FinnGen
Sami	Koskelainen			THL Biobank, Finnish Institute for Health and Welfare (THL)	Helsinki, Finland	FinnGen Teams member	FinnGen
Tero	Hiekkalinna			THL Biobank, Finnish Institute for Health and Welfare (THL)	Helsinki, Finland	FinnGen Teams member	FinnGen
Teemu	Paajanen			THL Biobank, Finnish Institute for Health and Welfare (THL)	Helsinki, Finland	FinnGen Teams member	FinnGen
Priit	Palta			Institute for Molecular Medicine Finland (FIMM), HiLIFE, University of Helsinki	Helsinki, Finland	FinnGen Teams member	FinnGen
Kalle	Pärn			Institute for Molecular Medicine Finland (FIMM), HiLIFE, University of Helsinki	Helsinki, Finland	FinnGen Teams member	FinnGen
Mart	Kals			Institute for Molecular Medicine Finland (FIMM), HiLIFE, University of Helsinki	Helsinki, Finland	FinnGen Teams member	FinnGen
Shuang	Luo			Institute for Molecular Medicine Finland (FIMM), HiLIFE, University of Helsinki	Helsinki, Finland	FinnGen Teams member	FinnGen

*Indicates required information. Only first name, last name, and suffix will appear in PubMed.

***First Name and Middle Initial(s)**	***Last Name**	***Suffix (eg, Jr, III)**	Academic Degrees	Institution	Location (city, state/province, country)	Role or Contribution, eg, chair, principal investigator	Group (if more than 1 Group listed in the byline) and/or Subgroup (eg, Steering Committee)
Vishal	Sinha			Institute for Molecular Medicine Finland (FIMM), HiLIFE, University of Helsinki	Helsinki, Finland	FinnGen Teams member	FinnGen
Tarja	Laitinen			Pirkanmaa Hospital District	Tampere, Finland	FinnGen Teams member	FinnGen
Mary Pat	Reeve			Institute for Molecular Medicine Finland (FIMM), HiLIFE, University of Helsinki	Helsinki, Finland	FinnGen Teams member	FinnGen
Marianna	Niemi			University of Tampere	Tampere, Finland	FinnGen Teams member	FinnGen
Kumar	Veerapen			Broad Institute	Cambridge, MA, United States	FinnGen Teams member	FinnGen
Harri	Siirtola			University of Tampere	Tampere, Finland	FinnGen Teams member	FinnGen
Javier	Gracia-Tabuenca			University of Tampere	Tampere, Finland	FinnGen Teams member	FinnGen
Mika	Helminen			University of Tampere	Tampere, Finland	FinnGen Teams member	FinnGen
Tiina	Luukkaala			University of Tampere	Tampere, Finland	FinnGen Teams member	FinnGen
Iida	Vähätalo			University of Tampere	Tampere, Finland	FinnGen Teams member	FinnGen
Jyrki	Pitkänen			Institute for Molecular Medicine Finland (FIMM), HiLIFE, University of Helsinki	Helsinki, Finland	FinnGen Teams member	FinnGen
Marco	Hautalahti			Finnish Biobank Cooperative - FINBB	Helsinki, Finland	FinnGen Teams member	FinnGen
Johanna	Mäkelä			Finnish Biobank Cooperative - FINBB	Helsinki, Finland	FinnGen Teams member	FinnGen
Sarah	Smith			Finnish Biobank Cooperative - FINBB	Helsinki, Finland	FinnGen Teams member	FinnGen

*Indicates required information. Only first name, last name, and suffix will appear in PubMed.

***First Name and Middle Initial(s)**	***Last Name**	***Suffix (eg, Jr, III)**	Academic Degrees	Institution	Location (city, state/province, country)	Role or Contribution, eg, chair, principal investigator	Group (if more than 1 Group listed in the byline) and/or Subgroup (eg, Steering Committee)
Tom	Southerington			Finnish Biobank Cooperative - FINBB	Helsinki, Finland	FinnGen Teams member	FinnGen
Andres	Metspalu						Estonian Biobank Research Team
Tõnu	Esko						Estonian Biobank Research Team
Mari	Nelis						Estonian Biobank Research Team
Lili	Milani						Estonian Biobank Research Team
Reedik	Mägi						Estonian Biobank
Georgi	Hudjashov						Estonian Biobank
