# Supplementary material for: Genetic Risk Factors Associated With Preeclampsia and Hypertensive Disorders of Pregnancy
Source: JAMA Cardiol. 2023 Jun 7;8(7):674–83. doi: 10.1001/jamacardio.2023.1312 (PMC10248811; doi:10.1001/jamacardio.2023.1312)
Supplement: Supplement 4. — Data sharing statement [file jamacardiol-e231312-s004.pdf]

Data Sharing Statement

Tyrmi. Genetic Risk Factors Associated With Preeclampsia and Hypertensive Disorders of Pregnancy. *JAMA Cardiol*. Published June 07, 2023. doi:10.1001/jamacardio.2023.1312

Data
Data available: Yes
Data types: Data (not involving human participants)
How to access data: Summary statistics of the meta analysis are accessible via GWAS Catalog (https://www.ebi.ac.uk/gwas/). Access to the cohort-wise summary information can be applied via contacting FINNPEC, FinnGen and EstBB customer service.
When available: With publication

Supporting Documents
Document types: None

Additional Information
Who can access the data: Meta analysis summary data will be available to all interested parties. Cohort-wise data is accessible to researchers whose proposed use of the data has been approved in the cohort in question.
Types of analyses: Summary statistics are freely available for any purpose.
Mechanisms of data availability: Summary data in GWAS Catalog without investigator support.
